# Strong exciton coupling: a practical toolbox for computing interaction energies, wavefunctions, and optical spectra

**DOI:** 10.1039/d6cs00157b

**Published:** 2026-05-19

**Authors:** Rasmus Ringström, S. Rasoul Hashemi, Yuanxin Liang, Nicholas J. Hestand, Karl Börjesson

**Affiliations:** a Department of Chemistry and Molecular Biology, University of Gothenburg, Box 462 405 30, Gothenburg Sweden karl.borjesson@gu.se; b Department of Natural and Applied Science, Evangel University 1111 N. Glenstone Ave Springfield MO 65802 USA

## Abstract

The perceived colour and photophysical properties of organic dyes depend not only on their chemical structure, but also how they pack together. When two dyes approach one another, the transition dipole moments associated with their molecular states begin to interact. If this interaction is sufficiently strong, it can lead to hybridization of the excited states, forming new hybrid states with altered photophysical properties. This phenomenon, formally denoted strong exciton coupling, is commonly associated with the terms J- and H-aggregates. Examples are abundant in nature, but less common in technology, though their number may increase as understanding and methods become more developed. Representative examples include light-harvesting complexes in cyanobacteria, which contain strongly coupled bacteriochlorophyll units, and the autumn colours of leaves, which are a function of the packing of carotenoids. In this review, a brief historical outlook is given, followed by a more in-depth discussion of various levels of theories used to model the properties of the hybrid states, ranging from Coulombic exciton models to extensions that incorporate vibronic coupling and charge-transfer interactions. Worked examples are included as tutorials to connect the theoretical frameworks to experimental observables. Finally, a personal reflection on directions where strong exciton coupling can have scientific and technological impact is given.

## Abstract 2

This review presents a practical toolbox for modelling exciton coupling in molecular aggregates, from Coulombic interactions and vibronic coupling to charge-transfer effects.

## Graphical abstract

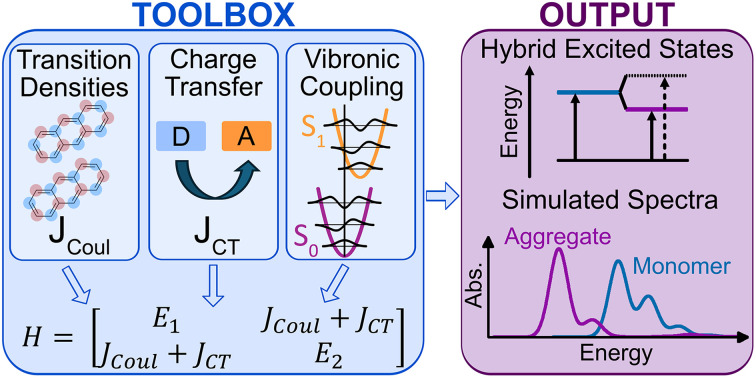

**Key learning points**

(1) Understand what strong exciton coupling is

(2) Compare the main approaches for calculating Coulombic exciton coupling

(3) Compute energies and oscillator strengths for hybrid states arising from exciton coupling

(4) Include vibronic coupling for improved prediction of energies, intensities, and spectral line shapes

(5) Account for short-range charge-transfer interactions alongside Coulombic coupling, and evaluate when each dominates.

## 1. Introduction

The interaction between light and matter is all around us. Leaves are green and jeans are blue because the dyes chlorophyll and indigo selectively absorb different parts of the visible spectrum. The selective absorption is a result of the dyes absorbing light quanta, photons, only when the energy separation between the ground and an electronically excited state equals the photon energy. However, to describe the light–matter interaction, it is better to consider light as an oscillating electromagnetic field. When a ray of light approaches a dye, the electric component of the field interacts with the dye through electric-dipole coupling. The oscillating electric field causes the dye to flicker (induces a coherent superposition) between its ground and excited electronic states. These two states have a different distribution of their electron densities, and the flickering can therefore be described as a transient electric oscillation. This oscillation is represented by the transition dipole moment, which is a complex vector whose magnitude and direction describe the strength and orientation of the electronic transition. The interaction between a dye and light is thus based on the electrostatic interaction between the oscillating electric fields of the transition dipole moment and light.^1,2^

The transition dipole moment is not restricted to interactions with light. Any oscillating electric field can interact with it, given that the frequency is matched. Therefore, when two dyes approach each other, their transition dipole moments will interact and give rise to a Coulombic interaction energy (or coupling). This interaction energy will depend on the size, distance, and orientation between their transition dipole moments in accordance with Coulomb's law. If the interaction energy is sufficiently large, the individual excited states hybridize to form two new hybrid states. The two-dye system is then in the so-called strong exciton coupling regime. The word exciton originates from the framework of solid-state physics and denotes a bound electron–hole pair. In molecular photophysics, and for this review, the word exciton is used interchangeably with the molecule's electronic excited state. Because the coupling is Coulombic, it is long-range and does not require orbital overlap. The phenomenon therefore appears in both non-covalent and covalent chromophore assemblies. When the coupling is appreciable, the electronic excitation becomes delocalized over the participating chromophores, forming excited states that extend across the assembly. The delocalization results in altered photophysics on several levels, which will be gone through in depth herein.

Our interest in this subject is currently two-fold. First, the aforementioned delocalization leads to a reduction in the reorganization energy of the excited state. A reduced reorganization energy leads to a decreased non-radiative rate constant, in accordance with the energy gap law.^3,4^ It has been suggested that strong exciton coupling therefore can be used as a design strategy to increase the emission yield in the near-infrared.^5,6^ We have shown that this is indeed possible using J-aggregates of quaterrylene.^7^ As making highly emissive near infrared dyes has proven immensely difficult, and having such is of technological and biomedical importance, strong exciton coupling can provide a design opening. Second, only states having an associated transition dipole moment of significant magnitude will be coupled together. Because the T_1_ ↔ S_0_ transitions are formally forbidden in purely organic chromophores, with a resulting close to zero transition dipole moment magnitude, triplet states are largely unaffected by this coupling. We have therefore proposed that strong coupling can be used as a tuning knob for singlet–triplet energetics^8,9^ and shown that intramolecular strong exciton coupling in BODIPY oligomers selectively lowers the singlet state without affecting the triplet state.^10^ The technological relevance is for organic light emitting diodes, where a large fraction of the electrical excitation results in the population of triplet states from which photons are difficult to extract.^11^ More generally, understanding and accurately modelling exciton coupling is essential for interpreting and engineering the photophysics of molecular aggregates. The coupling dictates the linewidth and spectral shifts of the absorption and emission spectra. This, together with delocalization and modified radiative rates arising due to exciton coupling, governs energy and charge transport in organic semiconductors. Predictive models are therefore central to designing aggregates for light harvesting, photodetection, lasing, and emissive devices.

This review begins with a brief historical overview of the development of exciton coupling theory and molecular aggregation. The initial discovery of the phenomena will be covered, as well as important landmarks including both fundamental understanding and technological applications. Readers interested in a more detailed historical account are referred to ref. 12 and 13. The next four chapters, which constitute the bulk of the review, develop a practical pathway for modelling strong exciton coupling. We begin by outlining how Coulombic couplings can be evaluated from transition densities. We then show how these couplings are used in a purely electronic exciton Hamiltonian to predict the photophysics of aggregates from monomer properties. Next, vibronic coupling is introduced to account for vibrational structure and line shapes. Finally, we discuss how charge-transfer interactions can mix with excitonic states, particularly at short separations, and how this modifies spectra and dynamics. To aid the newcomer in this field, examples are used to exemplify calculations, and derivations of equations are given in the SI. The quaterrylene J-aggregate and BODIPY oligomers discussed above represent cases of inter- and intra-molecular strong exciton coupling, and data from these two studies will be used in examples throughout this review. Developed scripts used in this review are deposited at Github††The code used to calculate TrC(ESP), TDC, and TrC(Mulliken) presented in this paper is available at https://github.com/srhashemi/EECC. and large output files from calculations are deposited at the Swedish National Data Service.‡‡All raw data used in this review is available at the Swedish National Data Service at https://doi.org/10.5878/pyja-rd94. Furthermore, nomenclature when modelling strong exciton coupling often originates from solid state physics. Care is taken to either explain the meaning of terms or use nomenclature from molecular photophysics. The penultimate chapter will deal with the properties, spectroscopic signatures and the excited state dynamics of the hybrid states. Lastly, an outlook that features our views on future developments and applications within this field is given.

## 2. Historical perspective

**Fig. 1:**
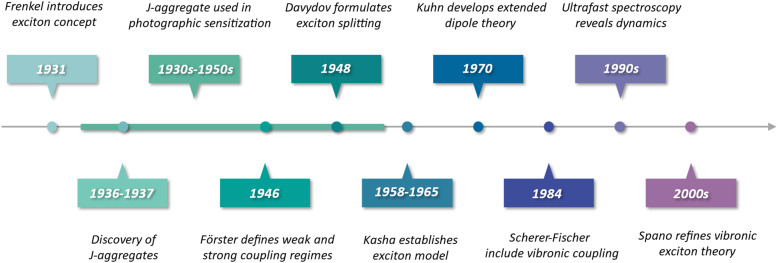
Summary of selected milestones in the development of exciton coupling theory and experiment, from early work on molecular crystals to modern vibronic exciton models for aggregates and covalently linked chromophore arrays.

Jelley and Scheibe independently discovered that cyanine dyes exhibit a remarkably sharp and intense red-shifted absorption band in some solvents, publishing their works within one month of each other in 1936–1937 (Fig. 1).^14,15^ This feature, now recognized as the J-band (named after Jelley), was found when deliberately inducing aggregation, and it revealed that non-covalent aggregates of dye molecules can form collective excited states, distinct from those of the isolated chromophores. In contrast, aggregates displaying blue-shifted absorption accompanied by quenched or depleted fluorescence were later designated H-aggregates (from hypsochromic shift). These fundamental distinctions in spectral response provided the earliest experimental evidence that interactions between molecular transition dipole moments can alter the photophysics in molecular suspensions at room temperature.

At the time of these discoveries, the conceptual idea that intermolecular interactions potentially could affect the photophysics was circulating. Jelley therefore attributed the red-shifted absorption to a resonance effect.^14^ Frenkel had introduced the exciton model in 1931,^16^ where he treated excitations in a crystal of atoms as a coherent superposition of states, describing how electronic excitations can be delocalized over the lattice of atoms. In the 1940s, theorists expanded the work of Frenkel to molecules, formalising how electronic coupling in molecular crystals gives rise to new collective excited states. The transition from atoms to molecules should be viewed from the perspective of symmetry. Atoms have a spherical symmetry, and when expanding the theory to encompass the less symmetrical molecules, the directionality of the molecules (or more precise, their transition dipole moments) relative to each other's needed to be included. Förster described the limiting cases when coupling is weak, resulting in fast energy transfer, and strong, resulting in collective excited states.^17,18^ By doing so he could rationalize the optical observations made on Scheibe's aggregates to internal packing. Davydov used anthracene crystals to introduce the notion of exciton splitting (Davydov splitting) of energy levels in solids.^19,20^

By the late 1950s, Kasha and co-workers further developed the molecular exciton model for aggregates, which treated interacting transition dipoles as point oscillators and successfully rationalized the spectral shifts in dimers and linear chains.^21–23^ Kasha's exciton theory showed that the relative orientation of molecular transition dipoles dictates whether the lowest-energy excited state is optically allowed or forbidden. Head-to-tail arrangements were found to produce the red-shifted, strongly emissive J-type behaviour, while side-by-side arrangements generated blue-shifted, weakly emissive H-type spectra. This simple two-level coupled-oscillator model by Kasha established the fundamental interpretation of J- *versus* H-aggregate spectra and firmly implicated exciton delocalization as the origin of their optical properties. Around the same period, Merrifield extended Frenkel's picture by explicitly including charge-transfer configurations in molecular crystals and linear chains, showing how these effects also can lead to spectral shifts.^24,25^

The earliest technological impact of strong exciton coupling was in silver-halide photography, where cyanine dyes had long been used as optical sensitizers.^26^ It was later recognized that their exceptional sensitizing efficiency arises from the formation of J-aggregates within the emulsion layer. The narrow and intense J-band enabled efficient absorption across the visible and near-infrared regions, while exciton delocalization promoted rapid energy transfer to silver-halide grains. Although this practical application predates the discovery of strong exciton interactions, it illustrates how collective excited states directly enhanced photochemical device performance.

As research progressed, it became evident that Kasha's point-dipole approximation was insufficient for extended dye arrays. In 1970, Kuhn and co-workers introduced the “extended dipole” approximation, recognizing that the transition charge density of a polymethine dye is distributed along its molecular length rather than concentrated at a point.^27^ By representing each chromophore as a pair of oscillating charges separated by approximately the molecular length, Kuhn's extended dipole model significantly improved the agreement between calculated and observed spectral shifts compared to the point-dipole approach. Around the same time, careful experiments on oriented dye monolayers (Langmuir–Blodgett films) by Kuhn and Möbius provided direct support for these theoretical advances.^28^ They observed collective optical effects, including superradiance and exciton migration quenching, which were consistent with J-aggregates behaving as domains of coherently coupled chromophores rather than as isolated molecules. These studies introduced the idea of a finite exciton coherence length (or exciton domain) in molecular aggregates, meaning that the delocalized excited state spans a specific number of molecules (often on the order of 10–100 under ambient conditions) rather than the entire aggregate.

By the 1980s, it became apparent that electronic coupling alone could not account for the narrow vibronic structure of J-aggregate spectra. As observed in the earliest cyanine aggregates, aggregation led to pronounced linewidth narrowing and a strong suppression of vibrational progression, features incompatible with a purely electronic picture.^14,29^ The foundation for including electron–phonon interactions had been laid in solid-state physics by Holstein's small-polaron model in 1959, Gouterman in 1961 and by Philpott in 1971.^30–33^ In 1984, Scherer and Fischer used a variational Holstein Hamiltonian approach to incorporate the dominant intramolecular vibrational mode into the exciton Hamiltonian and showed that this treatment can quantitatively reproduce the absorption spectrum of a pseudo isocyanine J-aggregate.^29^ This result not only validated the earlier assignment of the J-band as the delocalized 0-0 vibronic transition, but also demonstrated how vibronic sidebands (the 0-1, 0-2 transitions) are suppressed or enhanced depending on excitonic coupling strength. Scherer and Fischer's study thus firmly established that exciton-vibrational interactions are essential to fully describe the optical line shapes of aggregates under strong coupling conditions.

In the ensuing decades, the excitonic coupling framework matured through extensive theoretical and spectroscopic efforts. Wiersma and Knoester applied ultrafast spectroscopy and refined modelling to probe exciton dynamics, disorder, and relaxation in J-aggregates, providing deeper insight into how static and dynamic disorder limit exciton coherence lengths.^34–39^ On the theoretical front, Spano, Soos, Scholz and co-workers spearheaded the development of comprehensive vibronic exciton and charge transfer models,^40–45^ which is the current state-of-the-art framework for strong exciton coupling in molecular assemblies.

By now, the theoretical toolkit for describing J- and H-aggregates has evolved from Kasha's simple dimer model into a sophisticated vibronic exciton theory capable of treating arbitrary aggregate sizes, incorporating vibrational fine structure, and predicting phenomena such as polarization-dependent spectra, superradiance, and exciton self-trapping. This evolution, driven by the unique properties of J- and H-aggregates, has greatly enriched our understanding of excitonic materials and continues to guide the design of new molecular aggregates with tuneable optical functions. The following sections build on this historical framework to examine how these concepts are implemented and exploited in both noncovalent aggregates and covalently bound chromophore arrays.

## 3. Methods for evaluating coulombic coupling

The electronic charge distribution of a molecule differs from one electronic state to another. For a transition between two states, such as for example the ground state and first excited state, the redistribution of electronic density associated with the transition is described by the transition density. In the simplest approximation, this transition density is represented by its transition dipole moment, ***μ***, which is the first moment of the full transition density. When two molecules are brought into proximity, their transition densities interact. The magnitude of this interaction, the Coulombic coupling, *J*^Coul^, determines the extent to which the excitation energies and excited-state character of the molecules are perturbed.

This section introduces several different methods with varying degree of complexity to calculate the Coulombic coupling, *J*^Coul^, between transition densities within a purely Coulombic framework. Section 3.1 presents the methods from a mathematical and conceptual perspective, whereas Section 3.2 illustrates their practical application through selected example calculations.

### 3.1 Coulombic interaction energy

Exciton–exciton coupling is the Coulombic interaction between the transition densities associated with two electronic excitations. At its most fundamental level, Coulomb's law gives the interaction between two point charges, *Q*_1_, and *Q*_2_, separated by a distance, *R*_12_, and with the vacuum permittivity, *ε*_0_:1
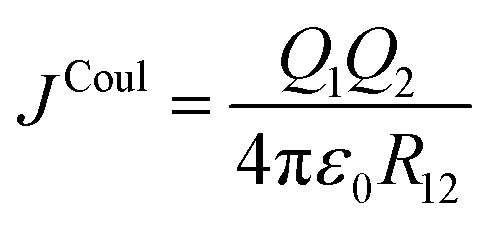


Equivalently, the interaction may be expressed in terms of the coordinates of the charges, ***r***_***i***_ = (*x*_*i*_,*y*_*i*_,*z*_*i*_), as2
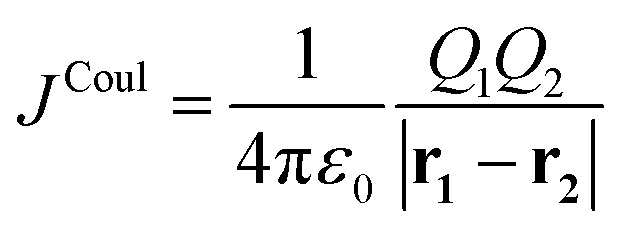


Throughout this review, *J*^Coul^ is used to denote the Coulombic exciton–exciton coupling energy, though in the literature alternative symbols such as *H* and *V* are also commonly encountered.

#### 3.1.1 From point charges to continuous distributions

Electronic excitations are described by continuous charge distributions rather than point charges. Consequently, the charge density *ρ*(**r**) is introduced. It is defined as charge per unit volume at position r in three-dimensional space. A small element of charge is d*Q* = *ρ*(**r**)d**r**, so the incremental interaction between two such elements located at **r**_**1**_ and **r**_**2**_ is3
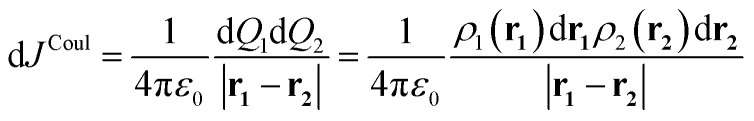


Integrating over both distributions yields the exact Coulomb interaction. In the context of exciton coupling, the relevant densities are transition densities, denoted *ρ*^tr^(**r**):4
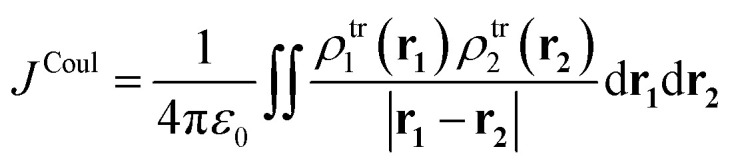


The transition density *ρ*^tr^(**r**) may be viewed as the ground-excited-state overlap density. It encodes how the electronic charge distribution oscillates during an optical transition, and it is the fundamental quantity that forms the interaction energy in Coulombic exciton coupling. In practice, *ρ*^tr^(**r**) is obtained from linear-response electronic-structure methods, such as time-dependent density functional theory (TD-DFT) or time-dependent Hartree–Fock/configuration interaction singles (TD-HF/CIS). These methods determine how the ground-state electron density responds to a weak time-dependent perturbation. The output of the calculations is the excited state together with a transition density matrix that represents the charge-redistribution pattern associated with that excitation.

#### 3.1.2 Direct evaluation of Coulombic coupling

Eqn (4) provides the exact Coulombic interaction between two transition densities. In practice, this expression must be evaluated in a numerical representation of the transition density. In the direct approach, the transition density is not replaced by a simplified model as will be seen for some other methods presented later. Instead, it is retained in its native quantum-chemical representation, most conveniently in an atomic-orbital (AO) basis.^46,47^ In this representation, the transition density is expanded as5
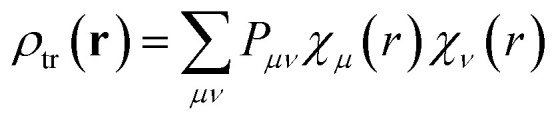


Here, *μ* and *ν* are indices running over all atomic-orbital basis functions of the molecule. Thus, *χ*_*μ*_(*r*) and *χ*_*ν*_(*r*) denote two specific AO basis functions and *P*_*μν*_ gives the contribution of their product to the transition density. The basis functions are defined by the chosen basis set, while the matrix elements *P*_*μν*_ are obtained from the excited-state calculation. Each product *χ*_*μ*_(*r*)*χ*_*ν*_(*r*) may be regarded as one finite, continuous “density piece”, and the total transition density is the weighted sum of all such contributions. Substituting eqn (5) into eqn (4) converts the continuous double integral into a double-sum expression over interactions between AO-pair density contributions on molecules 1 and 2:6
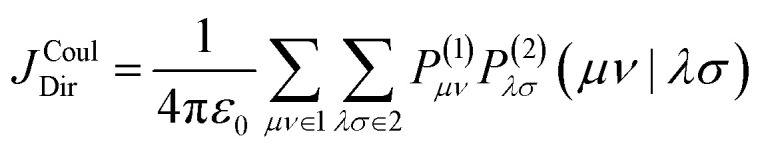


Here, *μ* and *ν* run over the AO basis functions of molecule 1, whereas *λ* and *σ* run over the AO basis functions of molecule 2. (*μν*|*λσ*) is a two-electron Coulomb integral,7
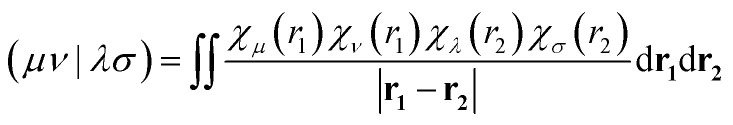
which represents the Coulomb interaction between one continuous AO-pair density contribution on molecule 1 and one on molecule 2. In this way, the total Coulombic coupling is obtained by summing all such pairwise interactions.

The direct method exhibits the potential to achieve one of the highest accuracies among the approaches considered herein because it avoids introducing an additional approximation in the representation of the transition density. The main practical drawback is that direct AO evaluation requires access to the transition density matrix and to routines that can evaluate and contract AO Coulomb integrals. These ingredients are available in many modern quantum-chemistry packages, although the degree to which the full workflow is directly accessible varies between programs. The matrop routine in the MOLPRO package is one example of such an implementation.^48^ Furthermore, the computational cost increases with basis-set size and with the number of couplings that must be evaluated. Also note that, as for essentially all methods discussed herein, the final result depends on the underlying electronic-structure method used to generate the transition density matrix.

#### 3.1.3 The transition density fragment interaction (TDFI) method

At the level of the Coulomb integral itself, the TDFI method, developed by Fujimoto in 2009, is very closely related to the direct AO-based evaluation described in Section 3.1.2.^47,49^ In both cases, the Coulombic coupling is evaluated as an interaction between the transition densities of the two chromophores in an atomic-orbital representation. In the TDFI formalism, this interaction may be written as8
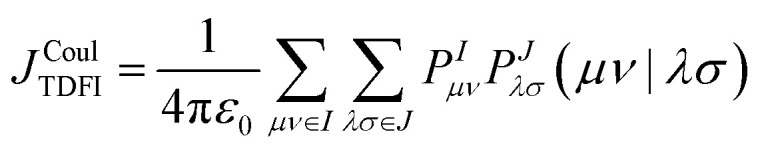
which has the same overall structure as the direct AO-based expression introduced above. The coupling is therefore again obtained as a double sum over pairwise Coulomb interactions between continuous AO-pair density contributions on fragments *I* and *J*.

The distinctive feature of TDFI is not the form of the Coulomb integral, but the way the fragment transition densities entering eqn (8) are generated. In a standard direct evaluation based on isolated monomers, the transition density of each molecule is calculated independently and the interaction between the two densities is then evaluated afterward. In TDFI, by contrast, the fragment densities are obtained self-consistently by means of the density-fragment interaction procedure.^50^ In this scheme, fragment *I* is calculated in the electrostatic field generated by fragment *J*, while fragment *J* is calculated in the field generated by fragment *I*. The fragment calculations are repeated iteratively until the total energy and the fragment densities no longer change significantly, that is, until a self-consistent solution has been reached. The resulting transition densities therefore correspond to fragments that are mutually polarized by their environment rather than to isolated molecules in vacuum.

This self-consistent treatment is physically important because the transition density of a chromophore can be modified by the presence of a nearby molecule. The neighbouring fragment changes the local electrostatic environment and can thereby alter the redistribution of electron density associated with the excitation. TDFI is designed to account for this effect before the Coulombic coupling is evaluated. In this sense, the method goes beyond a frozen-density picture and can provide more realistic Coulombic couplings when intermolecular polarization effects are non-negligible. Among the methods considered in this review, TDFI is unique in explicitly incorporating this mutual polarization at the level of the fragment transition densities.

Because TDFI combines AO-based Coulomb evaluation with self-consistent fragment densities, it can yield very accurate couplings. Its main drawback is that it is more computationally demanding than methods based on simpler transition-density representations, since the fragment calculations must be iterated to self-consistency before the coupling can be computed. For large molecular systems, or for applications requiring a very large number of couplings, such direct AO-based treatments may become impractical, which is one of the main reasons why approximate methods are still widely used.

#### 3.1.4 The transition density cube (TDC) method

The TDC method, developed by Krueger *et al.* in 1998, evaluates the Coulomb integral in eqn (4) by discretizing the transition density on a fine three-dimensional grid consisting of small voxels.^51^ It is the first method considered here that replaces the transition density by an explicit approximate representation rather than retaining it in the atomic-orbital basis. For the voxel centred at **r**_**i**_ with volume Δ*V*_**i**_, the associated transition charge is **q**_**i**_ = *ρ*^tr^(**r**_**i**_)Δ*V*_**i**_. The Coulombic coupling is then approximated by a double sum over all voxel pairs belonging to molecules 1 and 2:9
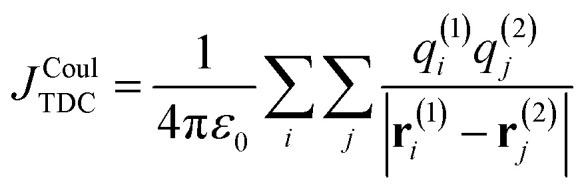


**Fig. 2:**
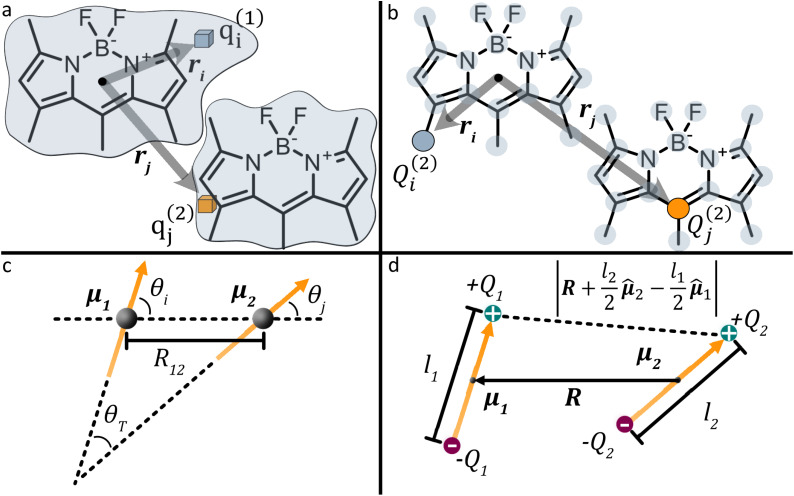
Schematic comparison of Coulomb-coupling approximations. (a) TDC: the transition density is discretized on a 3D grid. Two representative voxels *i* and *j* on molecules 1 and 2 carry transition charges *q*^(1)^_*i*_ and *q*^(2)^_*j*_ located at positions **r**^(1)^_*i*_ and **r**^(2)^_*j*_. The centre-to-centre separation is given by **R**. (b) TrC: the transition density is represented by a set of atom-centred transition charges. Shown are example charges *Q*^(1)^_*i*_ and *Q*^(2)^_*j*_ at atomic positions **r**^(1)^_*i*_ and **r**^(2)^_*j*_. (c) Point-dipole approximation: each transition density is reduced to a point dipole ***μ***_1_ and ***μ***_2_ separated by *R*_12_, with orientations *θ*_*i*_ and *θ*_*i*_ relative to **R** (and mutual angle *θ*_T_). (d) Extended-dipole: each transition dipole is represented by two charges ±*Q*_a_ separated by *l*_a_ along ***μ̂***_a_. *J*^Coul^ is obtained from the four pairwise charge–charge interactions between the two chromophores. For clarity, only one of the four charge–charge separations is shown 
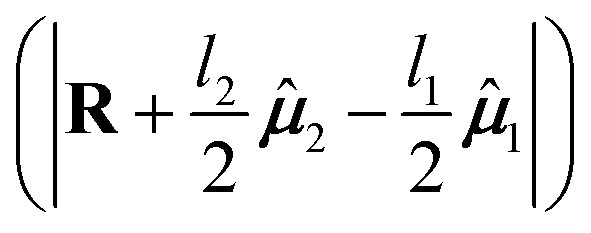
.

Here, *q*^(*n*)^_*i*_ is the transition charge associated with voxel *i* of molecule *n* and **r**^(*n*)^_*i*_ corresponds to the position vector of the respective transition charge as illustrated in Fig. 2a. In this way, the interaction between two continuous transition densities is approximated as the sum of Coulomb interactions between many small point charges distributed throughout space. The attainable accuracy is governed by the grid resolution: increasingly refined grids provide closer approximations to the exact Coulomb integral, albeit with a concomitant increase in computational expense.

In principle, the TDC method is capable of yielding highly accurate Coulombic couplings and an increasingly fine real-space grid provides an increasingly faithful discretization of the underlying transition density. Its accuracy may therefore approach that of direct AO-based evaluation. At short intermolecular separations, however, the method can encounter numerical difficulties because the transition-density cubes may overlap. When this occurs, the distances between voxel-centered point charges on the two molecules can become very small, leading to singularities or near-singularities in the Coulomb summation. To prevent this, the spatial extent of the cubes is often limited so that strong overlap is avoided. However, such truncation necessarily excludes part of the transition density and therefore introduces an additional source of error into the coupling.

#### 3.1.5 The transition-charge (TrC) method

A popular speedup condenses the continuous transition density to atom-centred charges. Instead of many thousands of grid voxels, a handful of atomic sites carry transition charges *Q*_*i*_ that represent the net contribution of the transition density around each nucleus. Accordingly, the coupling is expressed as a double sum over atom-centred transition charges on molecules 1 and 2 (eqn (10)), replacing the voxel-based sum used in the TDC approach.10
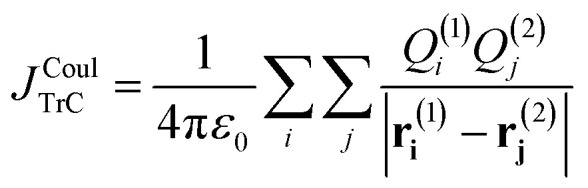


Here, *Q*^(*n*)^_*i*_ represents the transition charge on atom *i* of molecule *n* and **r**^(*n*)^_**i**_ corresponds to the position vector of the respective transition charge. Weiss^52^ and Chang^53^ pioneered replacing the continuous transition density with atom-centred transition charges and evaluating the coupling as a double Coulomb sum. Their treatments laid the groundwork for modern TrC approaches. Two contemporary routes are commonly used to obtain the atomic transition charges:

(1) Electrostatic-potential-derived (ESP) transition charges.^54^ In this method, the electrostatic potential of the *ab initio* transition density is first evaluated on a set of grid points distributed around the chromophore. This *ab initio* transition density is generally obtained from a linear-response calculation such as TD-DFT, CIS, or EOM-CC. For a transition density *ρ*^tr^(**r**), the corresponding transition electrostatic potential is11
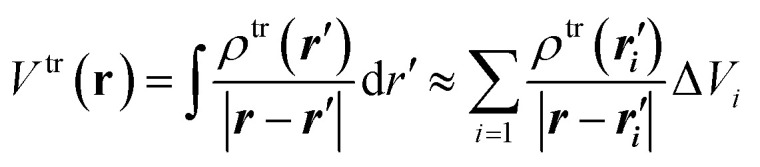


A set of atom-centred transition charges is then obtained by solving a weighted least-squares fitting problem that reproduces the electrostatic-potential values on the grid. Constraints are imposed to make sure that the fitted transition charges remain physically meaningful: the overall charge is neutral and the same dipole moments are reproduced.

In practice, one calculates the transition density for an optimized structure using a quantum chemistry package. The corresponding transition electrostatic potential is then evaluated on an ESP grid, and a chosen fitting scheme, most likely CHELPG, MK or RESP, is used to obtain atom-centred transition-ESP (TrESP) charges. The program Multiwfn provides reliable implementation of these fitting procedures, and additional details are given by Renger *et al.*^54^ Well-fitted TrESP charges reproduce the Coulomb coupling calculated obtained from the TDC method with far lower cost, since only tens to hundreds of fitted charges are required instead of the larger number of grid points used in TDC.

(2) The second method is based on Mulliken population analysis.^55,56^ Mulliken transition charges are obtained by applying the standard Mulliken population analysis to the transition density. The method partitions each AO–AO (atomic orbital) overlap population evenly between the two atoms associated with the basis functions, and the atomic transition charge is obtained by summing all contributions assigned to a given atom. This is simply the usual Mulliken decomposition of local and cross terms in the density, applied to the transition density rather than the ground-state density.

For general, non-orthogonal AO basis, the transition charge on atom X, *Q*^tr^_X_, can be written as12
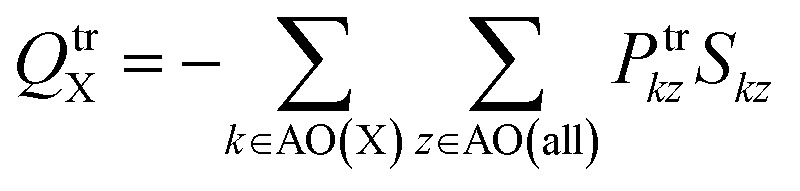
where *P*^tr^_*kz*_ is the AO-basis transition density matrix element, *S*_*kz*_ is the AO overlap matrix and the sum runs over AOs centred on atom X. Expanding the transition density matrix in terms of TD-DFT excitation amplitudes *A*_*ij*_ and molecular orbital (MO) coefficients gives the equivalent MO-level expression13
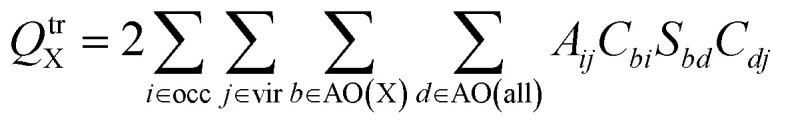
where *C*_*bi*_ is the coefficient of AO *b* on atom X in occupied MO *i*, and *C*_*dj*_ is the coefficient of AO *d* in virtual MO *j*. Eqn (13) reduces to a simpler form in an orthonormal AO basis; *i.e.* the off-term overlaps vanish by *S*_*bd*_ = *δ*_*bd*_, although this approximation can noticeably reduce accuracy. It is worth mentioning that the Mulliken charges are not inherently correct as the orbitals are equally divided between the participating atoms, regardless of actual spatial distribution of the density. Furthermore, they are very basis set dependent.^57^

The same basic consistency checks applied to TrESP charges should also be satisfied in Mulliken analysis, and in practice, the Multiwfn program can be used for computing the transition charges by applying Mulliken partitioning to the TD-DFT transition density matrix.

#### 3.1.6 More advanced atom-centred transition density models

The main advantage of the TrESP and Mulliken transition-charge methods is their simplicity and low computational cost. More advanced alternatives exist, however, which still retain the basic idea of representing the full transition density by atom-centred quantities on the interacting molecules. One such approach was introduced by Fujimoto in 2014 as the transition charge, dipole, and quadrupole from electrostatic potential (TrESP-CDQ) method.^58^ Compared with the conventional TrESP model, TrESP-CDQ incorporates two important improvements. First, it includes not only atom-centred transition charges, but also transition dipoles and quadrupoles, thereby providing a more detailed description of the local shape of the transition density. Second, the electrostatic-potential fitting is carried out using the same self-consistent transition densities as employed in the TDFI method, rather than transition densities obtained from isolated monomer calculations. In this way, TrESP-CDQ extends the original charge-only TrESP approach to a distributed transition-multipole model with improved accuracy.

A related, but more general, multipolar approach was later introduced by Góra and co-workers as the transition cumulative atomic multipole moments (TrCAMM) method.^59^ Like TrESP-CDQ, TrCAMM represents the transition density by atom-centred distributed multipoles rather than by charges alone. The key difference is that these multipoles are not obtained by fitting to the transition electrostatic potential. Instead, they are derived directly from the transition density matrix through the cumulative atomic multipole moments (CAMM) decomposition.^60^ TrCAMM therefore avoids the dependence on the fitting grid and fitting procedure that is inherent to TrESP-based methods, while still retaining the intuitive picture of atom-centred transition multipoles.^61^

#### 3.1.7 Multipole expansion

The methods presented in the preceding sections start from a quantum-chemically computed transition density (*e.g.*, *via* TD-DFT). In contrast, the models treated next do not generally require calculating a transition density matrix. Instead, they approximate the Coulomb interaction using only molecular geometry and low-order moments – most commonly the transition dipole moments (magnitudes and directions), which can be taken from experiment or simple estimates. These are long-range approximations that become reliable when the molecules are separated by distances much larger than the size of the chromophores themselves. In the regime where the centre-to-centre distance *R*_12_ greatly exceeds this spatial extent, the Coulomb kernel 1/|**r**_**1**_ − **r**_**2**_| can be systematically expanded in powers of small displacements about the molecular centres. This is a Taylor expansion, often referred to as a multipole expansion in electrostatics. Because a transition density integrates to zero net charge, the monopole term vanishes; the first nonzero contribution is the dipole–dipole term:14
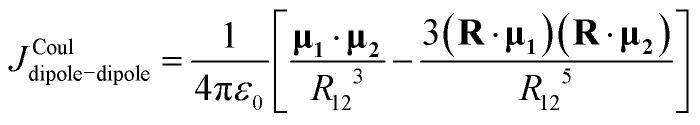
where **μ**_**n**_ is the transition dipole moment of molecule *n* and **R** is the displacement vector between transition dipole 1 and 2. The full derivation of eqn (14) from eqn (4) can be found in Section S2 of the SI. This expression is often presented in an alternative form using unit vectors (***μ***_***i***_ = |***μ***_***i***_|***μ̂***_*i*_ and ***R*** = |***R***|***R̂*** = *R*_12_***R̂*** where the “hat” indicates unit vector), as15
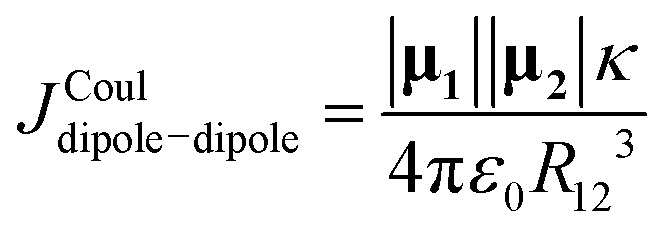
where *κ* is the orientation factor which is defined according to

16 *κ* = ***μ̂***_**1**_·***μ̂***_**2**_ − **3**(***μ̂***_**1**_·***R̂***)(***μ̂***_**2**_·***R̂***)

when the two transition dipoles and the inter-centre vector lie in the same plane, *κ* can be expressed in terms of the dipole–dipole angle *θ*_T_ and the angles *θ*_*i*_ and *θ*_*j*_ according to eqn (17), where the angles are defined in Fig. 2c.

17 *κ* = cos(*θ*_T_) − 3 cos(*θ*_*i*_)cos(*θ*_*j*_)

Higher-order terms in the Taylor expansion give rise to dipole–quadrupole, quadrupole–quadrupole, and successively higher interactions. Each successive term decays more rapidly with intermolecular separation. In general, the Coulombic coupling between multipoles of order *n* and *m* (*n*, *m* = 0 for monopole, *n*, *m* = 1 for dipole, *n*, *m* = 2 for quadrupole, *etc.*) scales with distance according to eqn (18).18
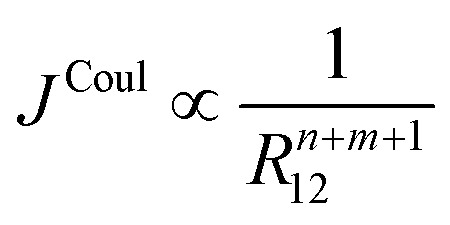


Which multipole interactions to include in practice depends on symmetry and transition character, and the desired level of accuracy. Strong, allowed transitions in relatively compact chromophores are usually well described by the dipole term beyond a few molecular diameters, whereas weak or symmetry-restricted transitions, or very anisotropic transition densities, may require inclusion of quadrupole or even octupole contributions at comparable separations. Note that in the case of dipole-allowed electronic transitions (*e.g.* for typical aromatic hydrocarbons), the leading nonzero Coulomb couplings are dipole–dipole, followed by dipole–octopole and octupole–octopole corrections. Terms involving even-parity transition moments – dipole–quadrupole, quadrupole–quadrupole, and magnetic-dipole–magnetic-dipole – are zero by symmetry.^62^

#### 3.1.8 Extended dipole approximation

The point-dipole limit described above assumes that each transition is localized at a point. The extended dipole approximation keeps the same Coulombic framework but acknowledges that a transition dipole moment has finite spatial extent.^63^ Each transition dipole on molecule *n* is represented by two opposite point charges ±*Q*_*n*_ separated along the dipole axis by a vector, **d**_**n**_, of length *l*_*n*_ as illustrated in Fig. 2d. The separation vector is taken parallel to the dipole direction ***μ̂***_*n*_ so that **d**_**n**_ = *l*_*n*_***μ̂***_*n*_ and the charge magnitude is fixed by the requirement that the model reproduces the transition dipole,19
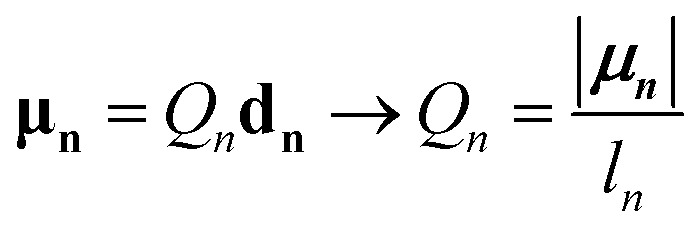


To derive the expression for the extended dipole approximation, let the molecular centres be **R**_**1**_ and **R**_**2**_ and define the centre–centre vector **R** = **R**_**2**_**− R**_**1**_. The Coulombic coupling is then the sum of the four charge–charge interactions. Written directly in terms of dipole magnitudes, dipole lengths, and directions, the general-geometry extended-dipole coupling is then20
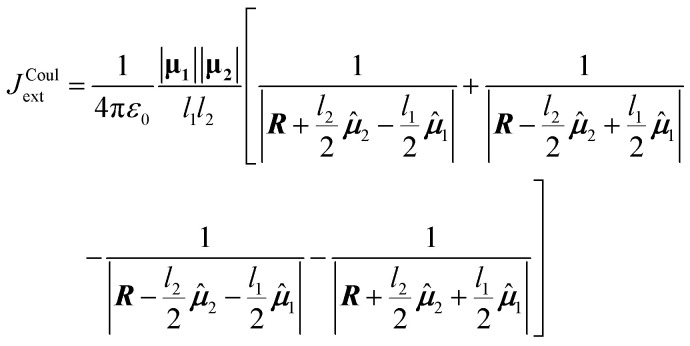


Each denominator is the distance between a specific pair of charges (two like-charge terms with positive sign, two unlike-charge terms with negative sign). The full derivation from eqn (4) is shown in Section S3 of the SI. In the far-field limit where *l*_*n*_ ≪ *R*_12_, a Taylor expansion of the denominators reproduces the dipole–dipole expression. At shorter separations the extended model often improves accuracy by incorporating finite spatial extent without resorting to a full transition-density calculation.

In summary, the approaches described in this section may be viewed as successive levels of approximation to the Coulomb integral in eqn (4). At the most rigorous end are the direct AO-based evaluation and the TDFI method, both of which evaluate the interaction in an atomic-orbital representation, with TDFI further incorporating self-consistent fragment polarization. The TDC method also aims to approximate the full Coulomb integral closely, but does so through a real-space grid representation of the transition density. Transition-charge methods such as TrESP and Mulliken, and their more advanced multipolar extensions TrESP-CDQ and TrCAMM, replace the continuous transition density by atom-centred charges or multipoles, thereby greatly reducing the computational cost while preserving much of the chemical intuition. The multipole expansion and extended dipole approximation constitute the most reduced descriptions, relying only on low-order moments and becoming quantitatively reliable primarily in the long-range regime. The appropriate choice of method therefore depends on the required balance between computational efficiency and quantitative accuracy.

### 3.2 Example calculations of *J*^Coul^

As noted in the introduction, a BODIPY system serves as a representative example in this review. In this section, we show how *J*^Coul^ can be evaluated for BODIPY oligomers using electronic structure calculations and a selection of the various methods discussed above.

**Fig. 3:**
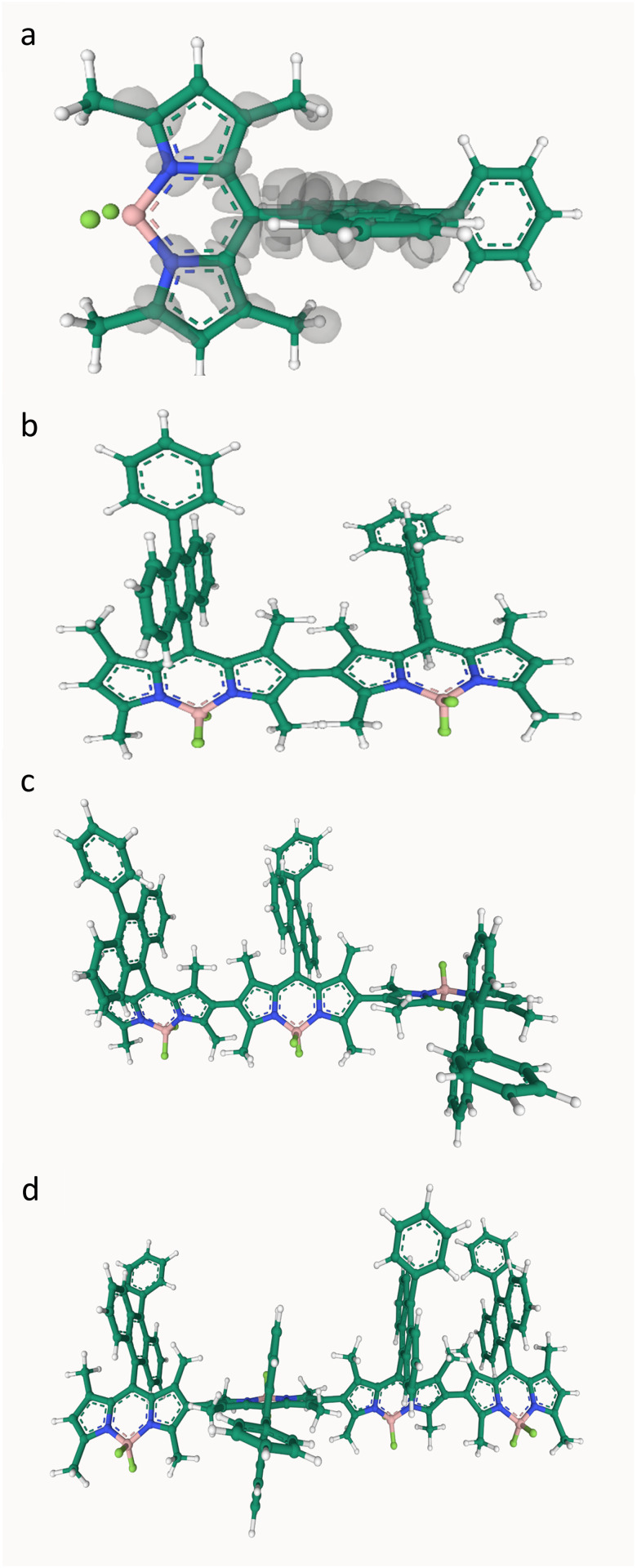
Geometry structures of the monomer (a), dimer (b), trimer (c) and tetramer (d) of BODIPY optimized at the ωB97XD/6-31g(d) level of theory. The transition density calculated for monomer is plotted in (a). Carbon, hydrogen, boron, nitrogen and fluorine are shown in dark green, white, pink, blue and pastel green, respectively.

Although the electronic structure calculations could be carried out in many ways and with a range of commercial or open-source codes, the TD-DFT method in the Gaussian 16 software^64^ is used here. Following Schäfer *et al.*,^10^ the ωB97XD functional^65^ and 6-31g(d) basis set were employed to optimize the geometries for the monomer, dimer, trimer, and tetramer structures shown in Fig. 3. It is worth mentioning that this initial geometry optimization is a necessary step for obtaining reliable results.

The TD-DFT calculations for the first excitation (S_0_ → S_1_) can be done on the optimized geometries by the keywords TD ωB97XD/6-31g(d) density = transition = 1 output = wfn. This instructs Gaussian to compute the transition density between the ground and first excited states at the chosen level of theory and to write the results to the .wfn file. Another practical point in Gaussian 16 is the need to save the checkpoint files (.chk) and then use the formchk utility to convert .chk to .fchk files. All the Gaussian input and output files are provided as SI.^§§^

Next, the generated .wfn and .fchk files are processed with the Multiwfn program^66^ to analyse the wavefunction and extract the Mulliken- and ESP-based transition charges, as well as the transition density for the BODIPY oligomers. The electron excitation analysis module is used to calculate the transition density cube file and Mulliken transition charges, while the TrESP module, together with CHELPG (charges from electrostatic potentials using a grid) fitting scheme, is employed to derive the ESP transition charges. We encourage the reader to consult the Multiwfn manual carefully to ensure the most reliable results.

With the transition density, transition charges, *XYZ* coordinates for the optimized oligomers, and dipole moments in hand, the exciton couplings can be evaluated using the models outlined in Sections 3.1.2–3.1.8. It is important to note, however, that the reliability of the computed *J*^Coul^ depends strongly on the quality of the underlying DFT calculations. The size of the system also plays a key role in choosing an appropriate model. For example, the computational cost of the TDC approach becomes prohibitively high for most oligomers. In practice, applying these models often require a degree of compromise between computational expense and the desired level of accuracy.

Table 1 summarizes the exciton Coulomb couplings obtained from TDC, TrC (ESP), TrC (Mulliken), point-dipole and extended dipole models for the BODIPY dimer, trimer and tetramer. Furthermore, the TDC coupling was calculated using both a direct and a fast Fourier transform (FFT)-based scheme. In the direct approach, all pairwise Coulomb interactions between the voxel charges of the two transition-density cubes are summed explicitly. In the FFT approach, the same coupling is evaluated more efficiently by first constructing the Coulomb potential generated by one cube and then integrating the second cube in this potential. The close agreement between the two results shows that both implementations are numerically consistent. Note that voxels carrying less than 0.05% of the largest voxel charge magnitude were neglected, which reduced the computational cost while still retaining about 98.9% of the total absolute transition charge for each monomer. In addition, to avoid singularities in the direct summation when the two transition densities partially overlap, any voxel–voxel distance smaller than 10^−10^ Å was replaced by 10^−10^ Å. The FFT scheme is free of this pairwise singularity because the Coulomb kernel is handled in reciprocal space with its *k* = 0 component set to zero. The TDC, TrC (ESP) and TrC (Mulliken) couplings reported here can be reproduced using our in-house python code,^§^ which is publicly available. For the point-dipole and extended-dipole calculations, we use the experimentally determined monomer transition dipole moment from ref. 10. The corresponding coupling calculations can be reproduced with our in-house MATLAB implementation (Jcoul_point_extended_dipole.m).

**Table 1:** Pairwise exciton couplings *J*^Coul^_*mn*_ (cm^−1^) for the BODIPY dimer, trimer, and tetramer calculated using TDC, TrC (ESP), TrC (Mulliken), point-dipole, and extended-dipole models. Point-dipole and extended-dipole couplings for all oligomer sizes were evaluated using monomer positions extracted from the geometry-optimised tetramer

Model	TDC (direct)	TDC (FFT)	TrC (ESP)	TrC (Mulliken)	Point-dipole	Extended-dipole
Dimer	*J*_12_ = −967	*J*_12_ = −990	*J*_12_ = −1140	*J*_12_ = −816	*J*_12_ = −821	*J*_12_ = −1811
Trimer	*J*_12_ = −954	*J*_12_ = −917	*J*_12_ = −1117	*J*_12_ = −803	*J*_12_ = −821	*J*_12_ = −1811
	*J*_13_ = −104	*J*_13_ = −120	*J*_13_ = −106	*J*_13_ = −95	*J*_13_ = −103	*J*_13_ = −119
	*J*_23_ = −936	*J*_23_ = −998	*J*_23_ = −1111	*J*_23_ = −784	*J*_23_ = −820	*J*_23_ = −1808
Tetramer	*J*_12_ = −937	*J*_12_ = −854	*J*_12_ = −1112	*J*_12_ = −784	*J*_12_ = −821	*J*_12_ = −1811
	*J*_13_ = −103	*J*_13_ = −128	*J*_13_ = −106	*J*_13_ = −94	*J*_13_ = −103	*J*_13_ = −119
	*J*_14_ = −12	*J*_14_ = −29	*J*_14_ = −30	*J*_14_ = −28	*J*_14_ = −30	*J*_14_ = −32
	*J*_23_ = −935	*J*_23_ = −1078	*J*_23_ = −1092	*J*_23_ = −791	*J*_23_ = −820	*J*_23_ = −1808
	*J*_24_ = −102	*J*_24_ = −135	*J*_24_ = −105	*J*_24_ = −94	*J*_24_ = −102	*J*_24_ = −119
	*J*_34_ = −953	*J*_34_ = −932	*J*_34_ = −1119	*J*_34_ = −802	*J*_34_ = −822	*J*_34_ = −1811

Table 1 shows a pronounced model dependence in the predicted couplings, with the extended-dipole estimate deviating most from the other approaches. Furthermore, while the TDC, TrC and point-dipole models predict couplings of the same order of magnitude, their numerical values still differ appreciably. Importantly, the extended-dipole model is sensitive to how the ±*Q* charges are positioned. For the values reported in Table 1, the charges were placed at the two terminal β positions of the BODIPY core (*i.e.*, the β-pyrrolic carbon atoms that define the tethering axis in the β-linked oligomers), so that the effective dipole spans the chromophore along the direction most relevant for intermolecular coupling. As the intercharge separation is reduced, the extended-dipole coupling continuously approaches the point-dipole limit. The choice used here (placing the charges at the ends of each monomer) therefore yields an upper bound within the extended-dipole framework. Consequently, care is required in selecting an appropriate dipole “extension” that reflects the spatial extent and orientation of the underlying transition density.

In general, the TDC and TrC methods are among the most reliable approaches considered in these example calculations, because they do not depend on the simplified long-range point-dipole approximation and can therefore account more effectively for short-range features of the transition-density interaction. In the present results, the TrC (ESP) couplings are consistently slightly larger than those obtained with the TDC method.

Despite this, previous reports have shown that transition-charge models can, in some cases, underestimate the Coulombic coupling.^59,67–69^ At the same time, the magnitude of this deviation is not universal, but depends on the intermolecular distance and relative orientation of the chromophores. Under certain geometrical conditions, the TrC method has been shown to yield couplings of accuracy comparable to those obtained from TDC.^54^

Finally, we note that the Coulombic couplings reported here were computed in the intramolecular setting, using the geometry-optimised structure of the covalently linked system. As a result, the individual pairwise couplings (*e.g.*, *J*_12_, *J*_23_, …) are not constrained to be identical, but reflect the slightly different relative orientations and separations of each neighbouring chromophore in the optimised geometry. For completeness, we also provide an intermolecular implementation,^§^ in which the monomers can be placed at user-defined positions and orientations to evaluate couplings for a chosen packing motif.

## 4. Exciton coupled systems and hybridisation

Having established how to calculate the interaction energy between molecules, attention now turns to how this coupling alters the photophysics of molecular assemblies. When the coupling is sufficiently strong, the excited states of the individual chromophores hybridize to form new states, analogous to the formation of molecular orbitals from atomic orbitals. These hybrid states are delocalized over the participating molecules and their photophysics are examined in detail in later sections. In the simplest model, the energy separation of hybrid states produced by this coupling is set by the coupling strength, *J*^Coul^. For a dimer of identical monomers, the difference in energy between the highest and lowest hybrid states (sometimes denoted the exciton bandwidth) is 2*J*^Coul^. For a longer, ideal nearest neighbor aggregate it is 4*J*^Coul^.^70^ In practice, the coupling strength required for these excitonic states to be spectroscopically resolved is often discussed in relation to the linewidth of the electronic transition of the individual chromophores. In essence, the broader the linewidth, the larger the coupling must be for the exciton structure to be clearly resolved. If the splitting is comparable to or larger than the linewidth, the exciton structure can usually be resolved, and the system is described as being in the strong-coupling regime. Conversely, if the excitonic splitting is much smaller than the linewidth, the states are often treated as effectively localized on the individual chromophores, and energy transfer is then well described by Förster-type resonance energy transfer.^18,71^ With these two definitions, numerous systems will fall in-between, being neither strongly nor weakly coupled. There is considerable literature on definitions of the strong and weak coupling regimes,^70,72^ we will therefore not linger on the regime of intermediate coupling, where the excited states are not well described as either fully localized or fully delocalized. Instead, we will describe theory and examples that lie firmly in the strong coupling regime. We will start with a qualitative vector approach, introduced by Förster and Kasha,^18,23,71^ and then continue with quantitative approaches based on setting up the Frenkel Hamiltonian for the coupled system. In the subsequent sections, the Frenkel Hamiltonian, which only considers the strict Coulombic interaction between pure electronically excited states, is modified to account for vibronic coupling and eventually also charge transfer interactions.

### 4.1 A qualitative approach for assessing coupling scenarios

To gain a qualitative understanding of how exciton interactions affect molecular photophysics, a vector approach can be used.^22^ The benefit of the vector approach is that it gives insights into the direction of the energy shifts and intensity changes of optical transitions without any formal computation. The drawback is that it does not give any insight into the magnitude of these changes as the coupling energy, *J*^Coul^, is not used. The coupling energy can thus be too small for the multi-chromophore system to enter the strong exciton coupling regime at all, and no effects would then be experimentally observed (even if changes have qualitatively been predicted).

The vector approach addresses whether an exciton interaction: (1) have a repulsive or attractive force, and thereby destabilize or stabilize the excited states, and (2) have a constructive or destructive interference of their transition dipole moments. The method can therefore be used to predict bathochromic (red) and hypsochromic (blue) shifts, as well as changed absorption/emission intensities. The method is graphical, where the transition dipole moments on the individual chromophores are drawn, with interchromophoric angles taken into consideration. As it is not quantitative, the limiting cases where the transition dipole moments being organized parallel, in a head-to-tail fashion, or in a circle are particularly instructive and will be used as examples herein. Each transition dipole moment is drawn as a vector, with the direction of the vector representing the phase of its oscillation and the length representing its magnitude. The interaction between two of them can be either repulsive or attractive based upon if their oscillations are in or out of phase.

**Fig. 4:**
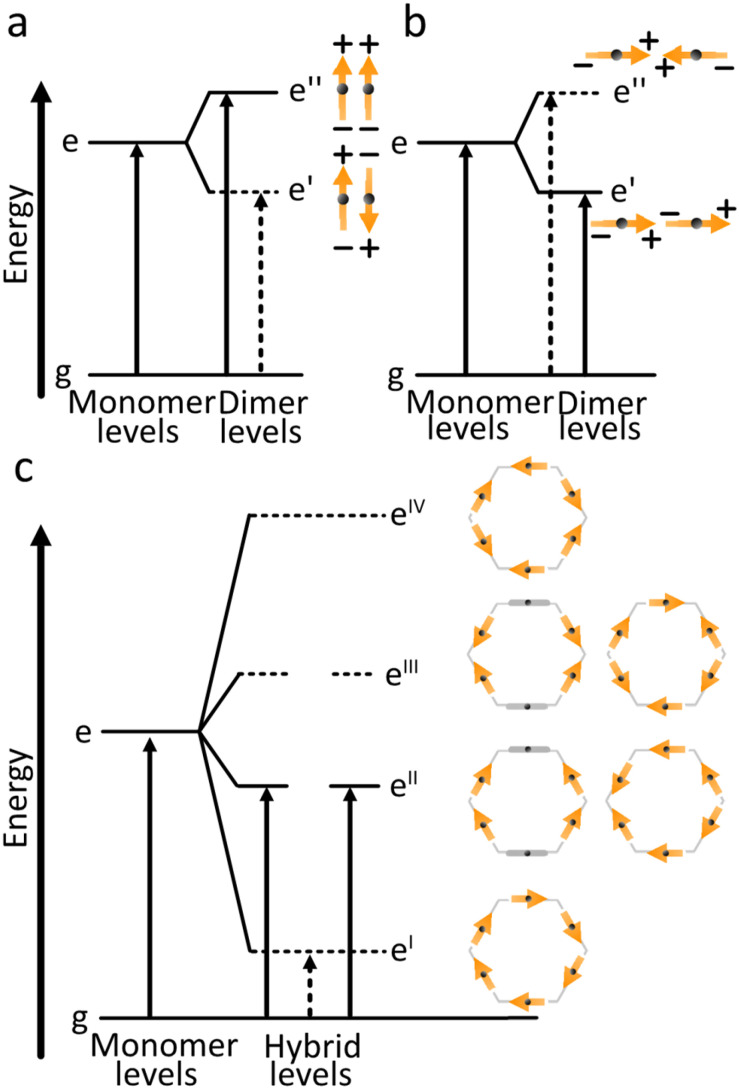
Exciton coupling scenarios. (a) Two parallel transition dipole moments that are in and out of phase. (b) Two head-to-tail oriented transition dipole moments that are in and out of phase. (c) Six transition dipole moments arranged in a ring give rise, through exciton coupling, to six delocalized hybrid exciton states. Their relative energies follow the same qualitative pattern as other six-membered ring systems (*e.g.*, benzene), with the lowest state optically forbidden and the first allowed transition occurring to the next higher, doubly degenerate pair.

Fig. 4a shows the case where two transition dipole moments are oriented in a parallel arrangement (side-by-side, H-aggregate). For the in-phase scenario, a repulsive force is experienced, with an accompanied increase in energy of the formed hybrid state compared to that of the individual monomers. The constructive interference of the in-phase oscillations enhances the oscillator strength for the transition to this state. For the out of phase scenario, an attractive force is experienced, lowering the energy of the formed hybrid state. However, the out of phase oscillations result in a net lowering of the oscillator strength for the transition (in the ideal scenario it is zero). Spectroscopically, a hypsochromic shift is observed for the parallel arrangement, and as the lowest excited state now is dark, no or very weak emission is typically observed.

Fig. 4b shows the case where two transition dipole moments are oriented in a head-to-tail arrangement. For this scenario, the in-phase oscillation is both attractive (reduced energy) and constructive (increased oscillator strength). Spectroscopically, a bathochromic shift of the absorbance is observed for this arrangement (J-aggregate), together with an increased rate of emission from the S_1_ → S_0_ transition (superradiance).

The model can also be used for more complex arrangements. The Anderson group has pioneered the synthesis of cyclic oligo-porphyrins. When comparing the radiative rate of six porphyrins in a linear configuration (head-to-tail arrangement, Fig. 4b) with that of six porphyrins in a cyclic arrangement (Fig. 4c), they observed a close to two orders of magnitude decrease in the radiative rate constant.^73^ This observation can qualitatively be explained as follows. Both cases display a head-to-tail arrangement, with the lowest energy state experiencing a constructive interference between all adjacent monomers. However, when summing the vectors of all transition dipole moments in the ring, the resultant becomes zero for the cyclic arrangement. The lowest energy transition is thus formally forbidden. This result is general in the sense that it applies to all exciton coupled systems that form a circular arrangement. It also forms a design principle saying that as oligo-dyes are arranged in an increasingly bent fashion, the oscillator strength for the S_1_ ← S_0_ transition will diminish.

The vector approach to account for exciton coupled systems can be expanded to involve an arbitrary number of chromophores. The energy of formed states will be arranged according to the number of repulsive/attractive interactions between adjacent chromophores, and the direction and magnitude of the resultant transition dipole moment of the formed hybrid states is given by the vector sum. This method can thus be used as a simple design tool for the construction of multi-chromophoric molecules. However, often the magnitude of energy level shifts is of interest, and then quantitative methods are needed.

### 4.2 Quantitative methods for assessing exciton coupling scenarios

The previous section introduced a qualitative picture of exciton coupling in simple model systems using the vector model. To quantify the interaction and predict the energies and intensities of the resulting hybrid states, a more formal treatment is developed here. Relevant literature are common textbooks on the subject such as from Parson,^74^ Agranovich^75^ and Kühn.^76^ Earlier sections established the Coulombic coupling, *J*^Coul^, between two chromophores from their transition densities. This coupling hybridizes the monomer excitations into new, delocalized hybrid states. Within the single-excitation (one-exciton) manifold, a convenient basis is formed by states in which exactly one chromophore is electronically excited, and all others remain in their ground states. Generally, a complete basis set containing all basis functions of an arbitrary number of molecular wavefunctions can be expressed as {|*n*〉}^*N*^_*n*=1_, where |*n*〉 = *φ*^g^_1_…*φ*^e^_*n*_…*φ*^g^_*N*_ represents the state with chromophore *n* electronically excited and all others in their ground states and *N* being the total number of molecules in the system. Here, *φ*^e/g^_*n*_ are the electronic wavefunctions of molecule *n* in the excited (e) or ground (g) states. For a three molecular system (*N* = 3) the basis functions describing the excited states are:

21 |1〉 = *φ*^e^_1_*φ*^g^_2_*φ*^g^_3_

22 |2〉 = *φ*^g^_1_*φ*^e^_2_*φ*^g^_3_

23 |3〉 = *φ*^g^_1_*φ*^g^_2_*φ*^e^_3_

Linear combinations of the basis functions will form the resultant wavefunctions of the hybrid states, *ψ*_*α*_, arising from strong exciton coupling. The coefficients, *c*^*α*^_*n*_, quantify what the contribution from each basis state is, and the sum of all squared coefficients for each hybrid wavefunction is 1.24
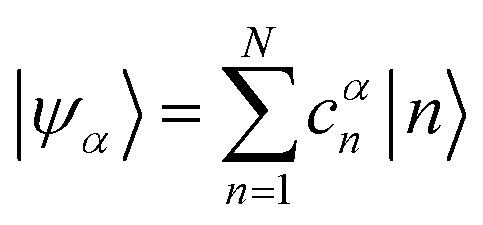
25
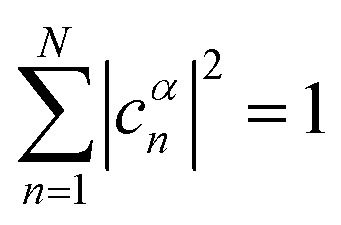


To get the energy and coefficients *c*^*α*^_*n*_ of the formed hybrid states, the Schrödinger equation, *ĤΨ* = *EΨ*, needs to be solved using a suitable Hamiltonian, *Ĥ*, to describe the system. Here, the system can be described by a so-called one-exciton Frenkel Hamiltonian, that is a sum of the Hamiltonians for the individual molecular wavefunctions and coupling terms between the chromophores.26
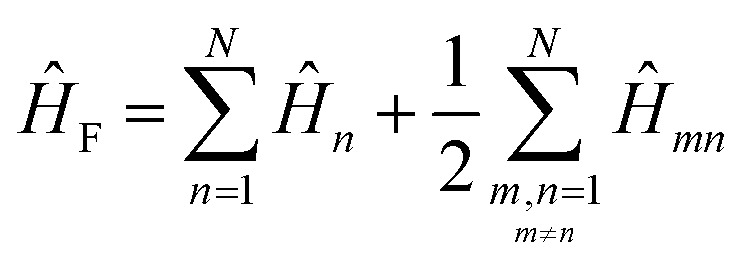


Eqn (26) can be expressed in bra-ket notation according to the following equations:

27 *Ĥ*_*n*_ = *ε*_*n*_|*n*〉〈*n*|

28 *Ĥ*_*mn*_ = *J*^Coul^_*mn*_(|*m*〉〈*n*| + |*n*〉〈*m*|)

where *ε*_*n*_ is the excitation energy of molecule *n*. The Hamiltonian compactly encodes everything needed to predict exciton energies of hybrid states through the time-independent Schrödinger equation. The easiest way to numerically solve the Schrödinger equation (*i.e.*, to find the system's eigenvalues and eigenvectors) is to use linear algebra solvers, which require expressing the Hamiltonian in matrix form. The multiplication of *Ĥ*_F_ with *Ψ* results in a set of linear equations. These can be expressed as one large matrix, *H*, which provides a concrete object that can be diagonalized to obtain energies of delocalized states. To construct the matrix, first choose an ordering of the basis states, for example (|1〉, |2〉, …, |*N*〉). The matrix elements of the operator *Ĥ*_F_ are then defined as

29 *H*_*nm*_ = 〈*n*|*Ĥ*_F_|*m*〉, *n*, *m* = 1, 2, …, *N*.

*i.e.*, “row *n*, column *m*” is the number obtained by sandwiching *Ĥ*_F_ between basis states 〈*n*| and |*m*〉. Evaluating the two sums in the Hamiltonian using orthonormality30
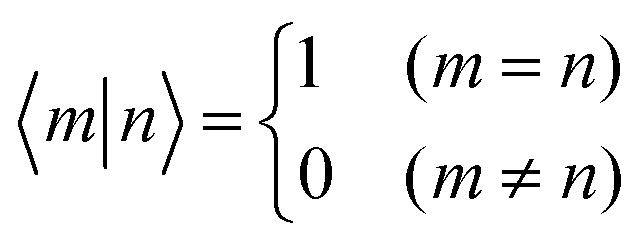
gives the diagon entries as the excitation energies, *ε*_*n*_, of the individual monomers in the relevant environment and the off-diagonal entries are the couplings *J*^Coul^_*mn*_ (*m* ≠ *n*). The derivation for the matrix construction is shown for a dimer in Section S4 of the SI. As an example, the resulting Hamiltonian matrix for three molecules (*N* = 3) is31
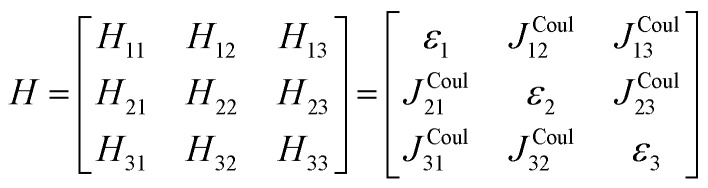
where *J*^Coul^_*mn*_ = *J*^Coul^_*nm*_ since *H* is Hermitian and *J*^Coul^_*mn*_ are real values. With the matrix defined, it is now possible to compute the excited state energies of the hybrid states by diagonalizing the Hamiltonian matrix:

32 *HC* = *CE* → *C*^−1^*HC* = *E*

33
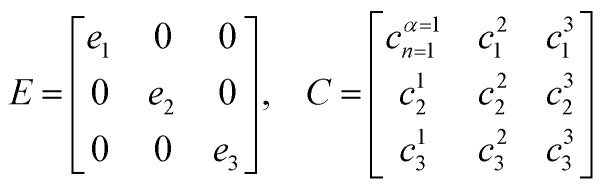


Here, *E* is the diagonal eigenvalue matrix containing the eigenvalues (formed hybrid state energies) and *C* is the eigenvector matrix containing eigenvectors corresponding to each eigenvalue. Each eigenvalue in *E* corresponds to a column of eigenvector coefficients in *C*. For example, the value in the first row and column of *E* (*e*_1_) corresponds to the eigenvector in the first column of *C*, that is, {*c*^*α*=1^_*n*=1_, *c*^1^_2_, *c*^1^_3_, …, *c*^1^_*N*_} and so on where *α* indicate the hybrid state in question. The eigenvalues represent the energy of the hybrid states that are formed due to exciton coupling. By considering the geometry of the specific molecular assembly under investigation along with the eigenvectors, one can further ascertain the oscillator strength of transitions to the formed hybrid states. This enables the determination of whether these transitions are allowed or forbidden, providing valuable insights into the system's electronic structure and optical properties. The transition dipole moment vector for hybrid state *α* from the ground state *g*, ***μ***_***α***_ = 〈*ψ*_*α*_|***μ̂***|*g*〉, is34
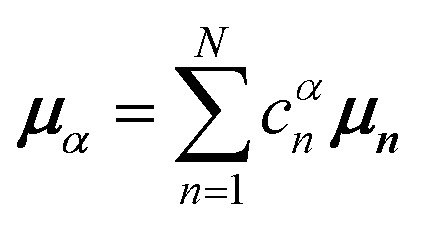
where ***μ***_***n***_ = 〈*n*|***μ̂***|*g*〉 is the transition dipole moment vector of monomer *n*, *c*^*α*^_*n*_ are the eigenvector coefficients of hybrid state *α* and |*g*〉 = *φ*^g^_1_*φ*^g^_2_…*φ*^g^_*N*_ represents the ground state in which all molecules in the aggregate are in their electronic ground state. Here, ***μ̂*** is the transition dipole moment operator defined as:35
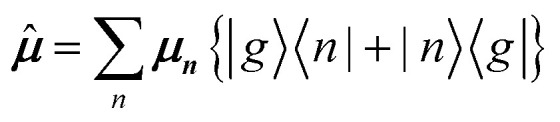


Note that the transition dipole moment operator ***μ̂*** should not be confused with the monomer transition dipole moment unit vector ***μ̂***_*n*_ introduced earlier. The former is an operator, whereas the latter denotes the direction of a monomer transition dipole.

Once the eigenvector coefficients, *c*^*α*^_*n*_, and the monomer transition dipoles, ***μ***_***n***_, are known, the oscillator strength of hybrid state *α* follows from

36 *f*_*α*_ ∝ |***μ***_***α***_|^2^

so that constructive or destructive interference between the local transition dipoles determines whether a given hybrid state is optically bright or dark. In the following section, this theoretical framework is applied to simple model aggregates with different geometries. By diagonalizing the corresponding example Hamiltonians and examining the resulting eigenvectors one can assess which hybrid states are expected to be more allowed or forbidden in the optical spectra.

#### 4.2.1 Example calculations

**Fig. 5:**
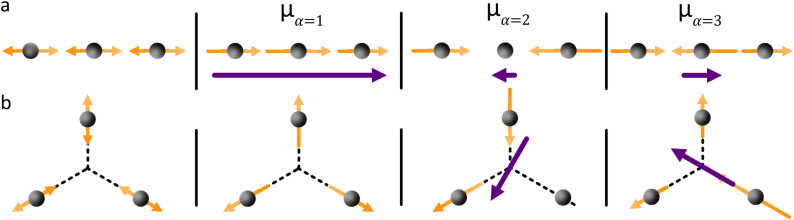
Example orientations of transition dipole moments in two *N* = 3 aggregates: (a) a J-aggregate-like arrangement and (b) a *C*_3_-symmetric trimer. Monomers are shown as grey spheres, individual monomer transition dipoles as orange arrows, and the net transition dipole moment of the resulting hybrid state as the purple arrow. Arrow lengths are proportional to the corresponding dipole magnitudes.

The first example is for a system with *N* = 3, and with the orientation of the monomers as illustrated in Fig. 5a (head-to-tail for all monomers, ideal J-aggregate). The coupling, *J*^Coul^, for such a trimer system was calculated in Section 3.2. Using the obtained coupling values from the TrC (ESP) method converted from cm^−1^ to eV and the monomer excitation energy, *ε*_*n*_ = 2.45 eV, one obtains the following Hamiltonian matrix:37
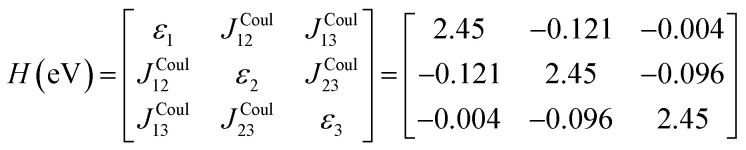


Diagonalization (performed *via* standard numerical diagonalization using for instance MATLAB's eig or similar routines) results in the following eigenvalues and eigenvectors:38
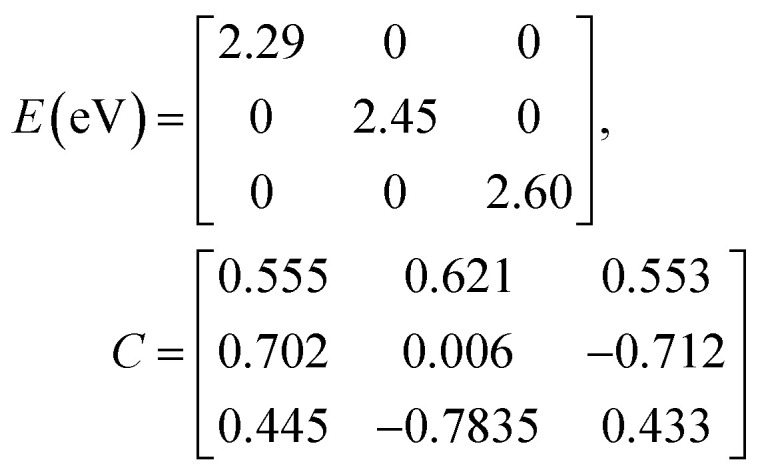


In this particular case, for the head-to-tail configuration, the direction of all monomer transition dipole moments is the same and with equal magnitude |***μ***| = |***μ***_1_| = |***μ***_2_| = |***μ***_3_|. The direction can be arbitrarily chosen to be the *x*-axis; ***μ***_***n***_ = |***μ***|***x̂***, where ***x̂*** is a unit vector along the *x*-axis. Consequently, each hybrid state's transition dipole moment is oriented in the same direction with a magnitude set by the scalar sum of the eigenvector column entries. For hybrid state *α* = 1, corresponding to the first column of the eigenvalue and eigenvector matrices:39
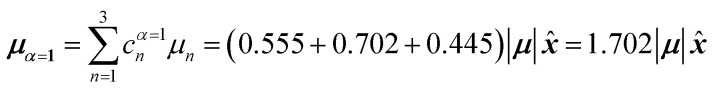
and for hybrid state *α* = 2 and *α* = 3:40
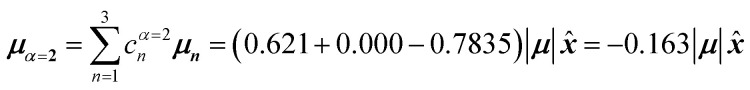
41
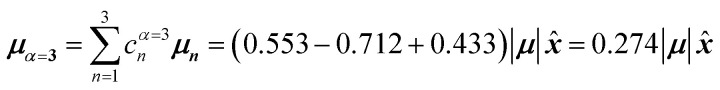


Thus, the head-to-tail (collinear) geometry concentrates essentially all oscillator strength in the lowest-energy hybrid state, as expected for a J-aggregate. The two higher energy hybrid states remain only weakly allowed. The middle state acquires a small but nonzero transition moment, and the highest-energy state is slightly brighter than the middle one, though both are far weaker than the dominant low-energy transition. The results of these calculations are depicted graphically in Fig. 5a.

Fig. 5b illustrates a case where the transition dipole moments are not colinear, exemplified by three identical monomers arranged in *C*_3_-symmetric geometry. This example highlights how the exciton-coupling framework extends naturally to higher-dimensional arrangements of transition dipoles. Using arbitrarily chosen coupling values and monomer excitation energies for this demonstration the following Hamiltonian matrix can be assembled:42
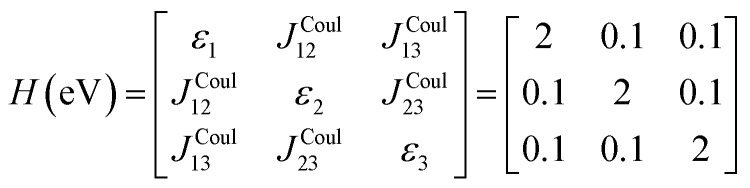


Note that due to the *C*_3_-symmetry all coupling strengths, *J*^Coul^_*mn*_, are equal. Diagonalization results in the following eigenvalues and eigenvectors:43
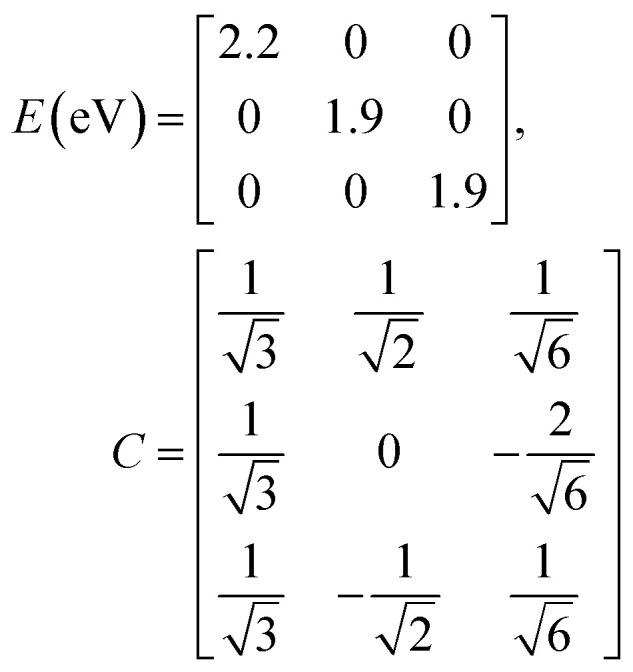


For equal magnitude transition dipole moments of the monomers oriented 120° apart in the plane yields 
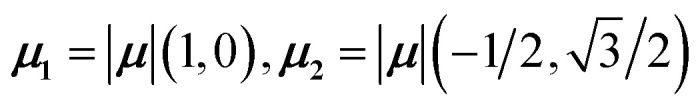
 and 
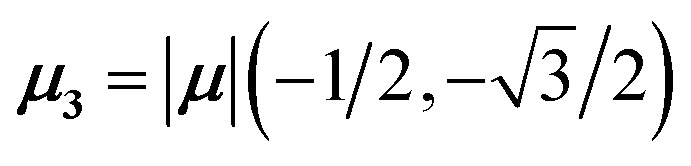
, where the pairs in parentheses give the Cartesian components in the orthonormal basis (***x̂***, ***ŷ***) in the *xy*-plane. The transition dipole moment of hybrid state *α* is therefore44
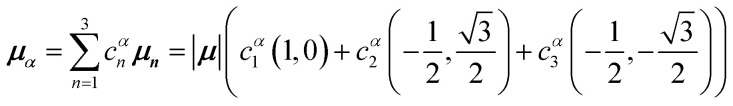


Thus, for hybrid state *α* = 1 corresponding to the first eigenvalue column and the first eigenvector column (evaluating *x* and *y* components separately)45
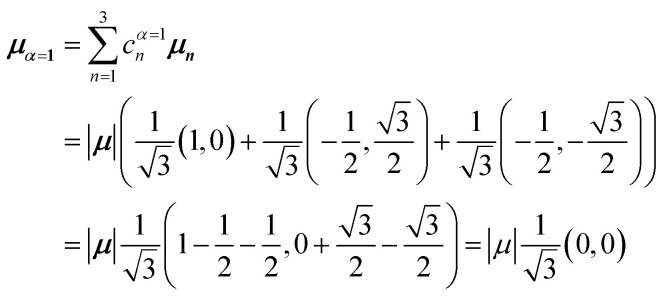
46
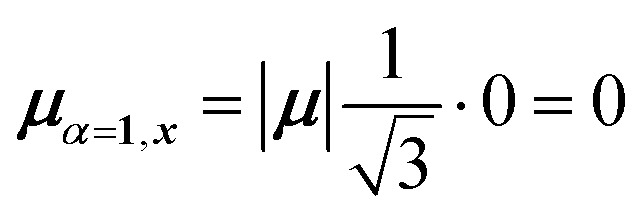
47
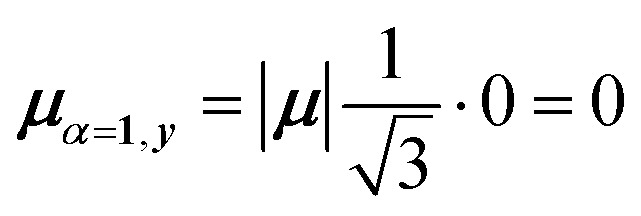


Evidently, for the symmetric hybrid state *α* = 1, both *x* and *y* components are zero, resulting in a dark state with zero transition dipole moment. A closely related symmetry-driven cancellation appears in the previously discussed six-membered ring (Fig. 4c), where both the lowest and highest hybrid states are dark and the first bright transition occurs to the doubly degenerate pair. A similar analysis for the two degenerate hybrid states *α* = 2 and *α* = 3 yields48
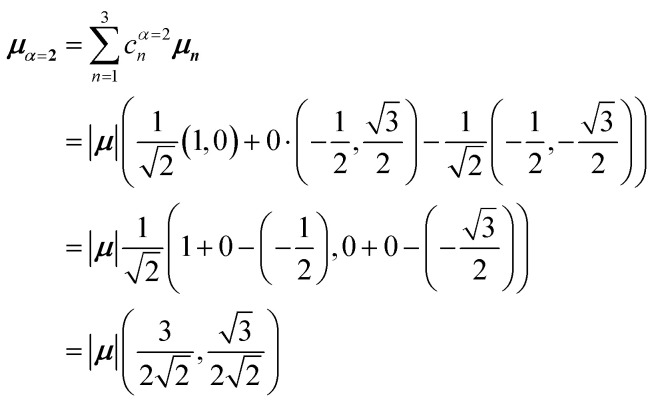
49
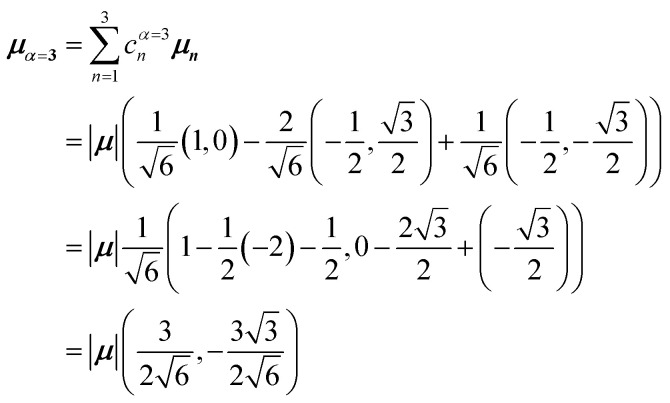


As a result, the two degenerate hybrid states, *α* = 2 and *α* = 3, have transition dipole moments with equal magnitude50
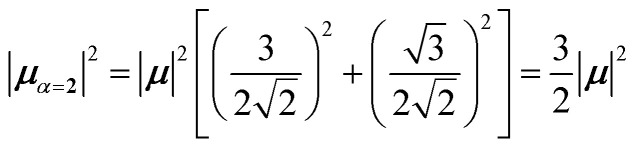
51
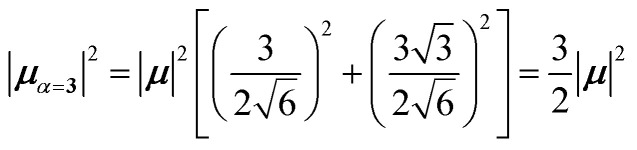
and the relative angles of the hybrid states’ transition dipole moments can be calculated by writing the vectors in polar form ***μ***_***α***,***x***_ = |***μ***|cos(*θ*_*α*_) and ***μ***_***α***,***y***_ = |***μ***|sin(*θ*_*α*_) where *θ*_*α*_ is the angle from the +*x*-axis which, for hybrid state *α* = 2 and *α* = 3, can be calculated as52
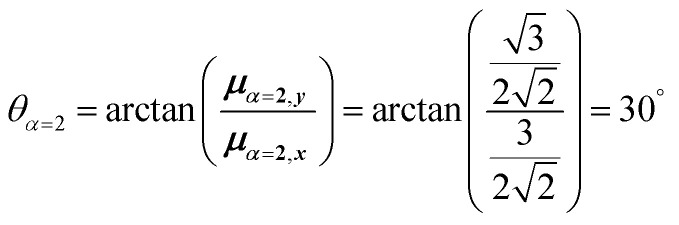
53
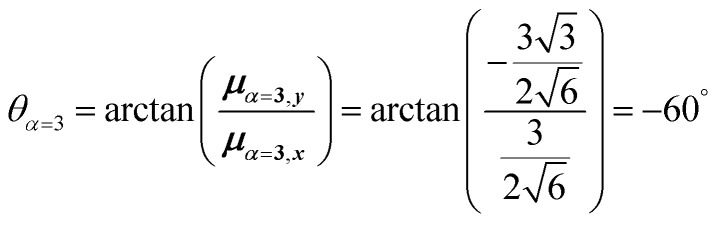


Thus, the transition dipole moments for the two degenerate states are at 90° relative to each other and with equal magnitude. They form a plane polarized transition as observed in for instance Zn-tetraphenylporphyrin and similar compounds assuming ideal *C*_3_-symmetry.^77^

Note that because Coulombic couplings decay with distance (dipole–dipole ∼ *R*^−3^), it is common to simplify *H* by setting *J*^Coul^ = 0 when *J*^Coul^ is below a certain value or when the distance exceeds a cutoff-value. This is a common simplification when using computationally heavy methods such as TDC to determine *J*^Coul^. For very long or periodic systems, nearest-neighbour (NN) or next-nearest neighbour (NNN) models can often be adequate.

## 5. Vibronic coupling

Up to this point the coupled transition densities have been treated as purely electronic. In organic molecules, however, electronic excitations are intertwined with intramolecular vibrations (vibronic coupling). To account for this coupling to a selected normal mode, the one-exciton Frenkel Hamiltonian from Section 4.2 can be extended to the Frenkel–Holstein form (single-excitation restriction retained):^75,76^

54 *Ĥ*_FH_ = *Ĥ*_F_ + *Ĥ*_vib_ + *Ĥ*_F–vib_

Here, *Ĥ*_F_ is the purely electronic part which is identical to the Frenkel Hamiltonian introduced in Section 4.2:55
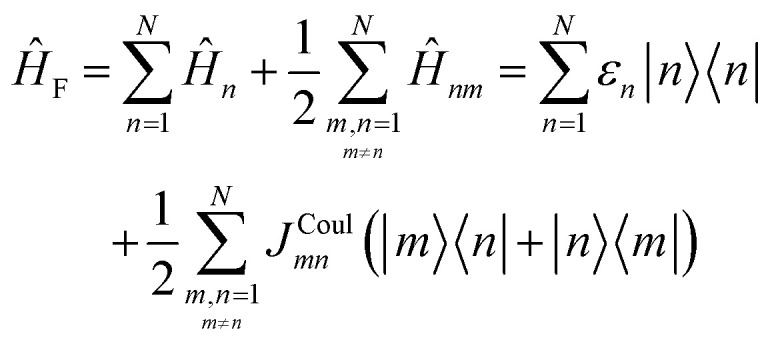


To account for the vibrational coupling, a set of annihilation and creation operators, *b*^†^_*n*_(*b*_*n*_), must be introduced. *b*^†^_*n*_(*b*_*n*_) creates (annihilates) one vibrational quantum in the ground state surface potential on molecule *n*. These are used to define the remaining terms of the Frenkel–Holstein Hamiltonian, which introduce the vibrational mode and its coupling:56
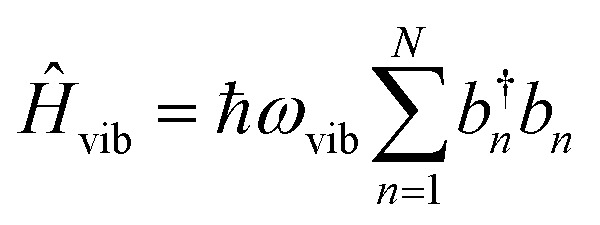
57
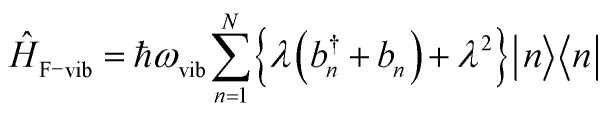


**Fig. 6:**
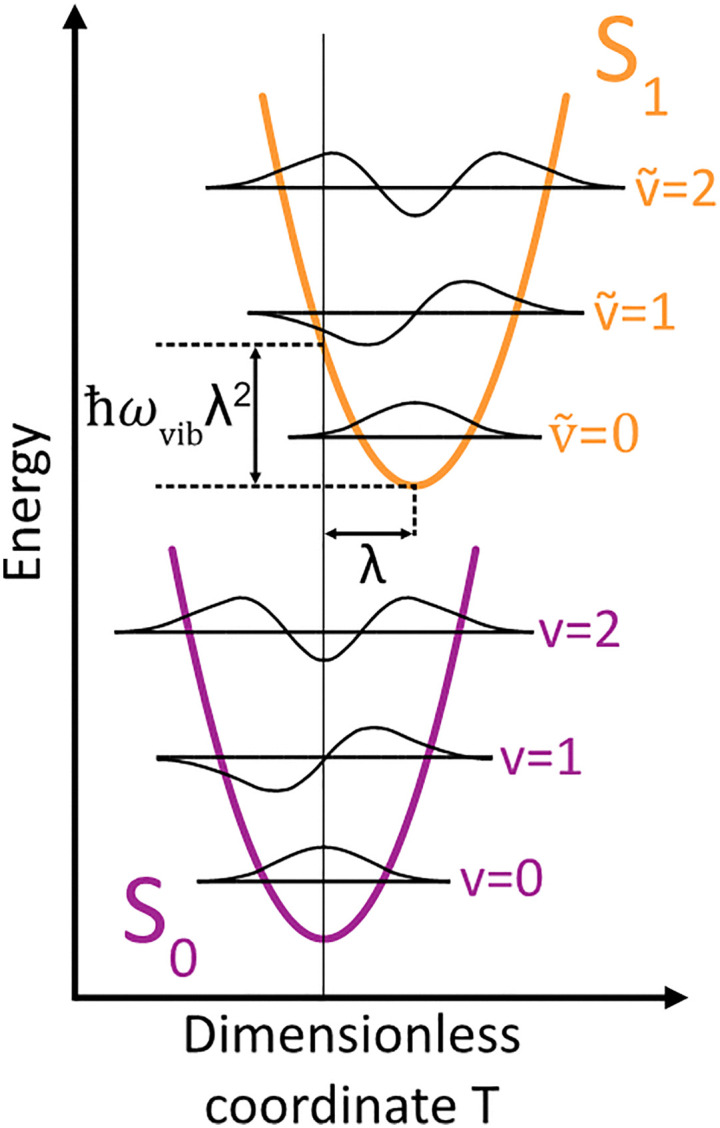
Schematic energy diagram for the harmonic vibrational potentials of ground (S_0_) and the displaced excited (S_1_) state along the dimensionless coordinate 
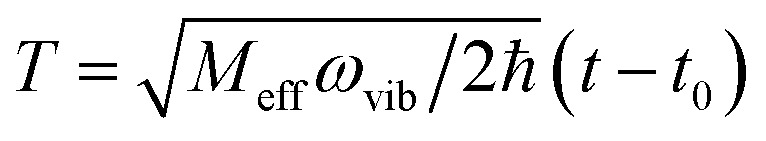
 where *M*_eff_ is the effective mass of the normal vibrational mode and *t*_0_ is the equilibrium position of the nuclei when the system is in its electronic ground state. The displacement of the ground and excited state's potentials is quantified by the square root of the Huang–Rhys factor. The vibrational wavefunctions of the excited state potentials are denoted with a tilde (∼) to distinguish them from the ground state vibrational wavefunctions.

*Ĥ*_vib_ accounts for the vibrational energy in the system. In this term, *b*^†^_*n*_*b*_*n*_ is a number operator that counts the number of vibrational quanta with energy *ħω*_vib_ on molecule *n*. The final term, *Ĥ*_F–vib_, accounts for the exciton-vibration coupling and the displacement/shift of the harmonic excited state potential surface relative to the harmonic ground state potential surface. Here, *λ*^2^ = *S* is the Huang–Rhys factor, which quantifies this displacement as shown in Fig. 6.

Practically, the Huang–Rhys factor, *λ*^2^, can be estimated from theoretical calculations or experimentally from the relative height of the 0-1 and 0-0 Gaussian absorption peaks of the monomer unit.^78^58
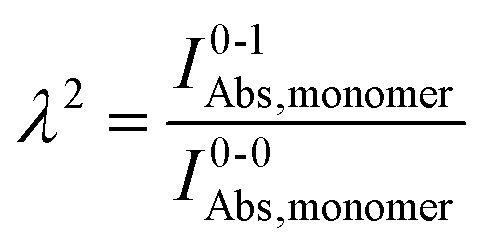


The vibrational mode's energy, *ħω*_vib_, can be estimated from the energy difference between the 0-0 and 0-1 vibronic peaks of the monomer spectrum. For many π-conjugated organic molecules the S_0_–S_1_ electronic transition is often coupled to a symmetric vinyl stretching mode with an energy of *ħω*_vib_ ∼ 0.15–0.18 eV.

### 5.1 Introducing a basis set

To solve the Frenkel–Holstein Hamiltonian *Ĥ*_FH_, a basis set that explicitly includes vibrational quanta is introduced. A convenient choice is the multiparticle basis set in which the term “particle” refers to an excitation which is either a purely electronic excitation, a purely vibrational excitation, or a vibronic (electronic/vibrational) excitation. Like the case of the Frenkel Hamiltonian, only a single electronically excited molecule is considered, all others are in the electronic ground state. A one particle state is written as

59 |*n*, *ṽ*〉

which denotes an electronic excitation localized on molecule *n* together with *ṽ* = 0, 1, 2 … vibrational quanta in the displaced (relative to the ground state potential surface) excited state potential of that molecule. All other molecules are in their electronic and vibrational ground states. Note that the tilde indicates quanta on the displaced excited state potential surface rather than the ground state. A two-particle state

**Fig. 7:**
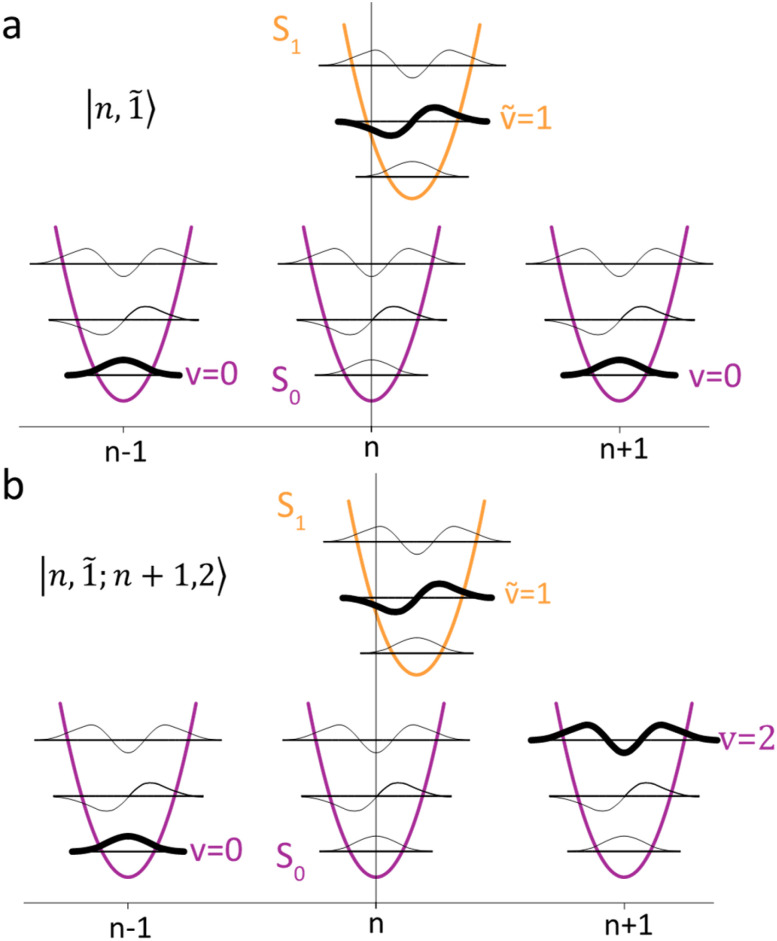
Illustration of the (a) one particle state, in which molecule *n* is in the electronic excited state (S_1_) and with vibrational quantum ***ṽ*** = 1 (indicated by the bolded line) and all other molecules are in their electronic ground states (S_0_) and vibrational ground states, |***n***, **1̃**〉. (b) Two particle state in which molecule *n* is once again in the electronic excited state and with vibrational quantum ***ṽ*** = 1. In addition, one molecule in the system (*n* + 1) has a vibrational quantum ***v*** = 2 and all other molecules (except *n* and *n* − 1) are in their electronic ground states and vibrational ground states, |***n***, **1̃**; ***n*** + **1**, **2**〉.

60 |*n*, *ṽ*; *n*′, *v*′〉, (*n*′ ≠ *n*, *v*′ ≥ 1)

describes an electronic excitation with *ṽ* vibrational quanta on molecule *n*, together with *v*′ vibrational quanta on a different molecule *n*′ in the ground electronic state. Higher-order states (three-particle, *etc.*) would add additional vibrational quanta on additional molecules. For many organic aggregates, truncation at the two-particle level, known as the two particle approximation, generally yields quantitatively reliable results and contributions from three or more particle states are typically small.^79^ A schematic illustration of the one- and two-particle state configurations is presented in Fig. 7.

An independent truncation caps the total number of vibrational quanta, *ν*_max_, that is included in the basis for computational feasibility and is expressed as61
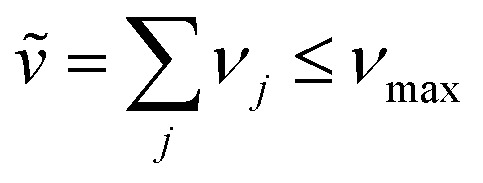


In this expression, *ṽ* corresponds to the number of vibrational excitations on the electronically excited molecule, while the summation accounts for vibrational excitations that may appear on other remaining molecules in their electronic ground states. The integer *ν*_max_ therefore defines the maximum total vibrational excitation allowed within the truncated basis. A practical choice for *ν*_max_ comes from the monomer's vibronic progression. It is generally advised to include at least levels up to and slightly beyond the last monomer band with non-negligible spectral intensity. For instance, if clear 0-0, 0-1, and 0-2 bands are visible and a faint 0-3 shoulder appears, choose at least *ν*_max_ ≥ 4 so the basis contains all the vibronic bands that appear in the spectrum. For the common ∼0.17 eV vinyl stretching mode, *ν*_max_ = 4 typically suffices for converged absorption/emission.^44^ However, convergence should be checked by increasing *ν*_max_ until energies and intensity ratios are stable.

With the basis fixed and ordered, the Frenkel–Holstein Hamiltonian is assembled as a finite matrix and the eigenvalue problem

62 *HC* = *CE* → *H* = *CEC*^−1^

can then be solved, where *E* is the diagonal matrix of eigen energies of the formed hybrid states, and *C* contains the corresponding eigenvectors.

### 5.2 Example calculation

As an example, with *N* = 2 and *ν*_max_ = 2, and using the two-particle approximation the basis ordering will take the following form for a one-particle basis (6 states):

63 **1**.|1, 0̃〉, **2**.|1, 1̃〉, **3**.|1, 2̃〉, **4**.|2, 0̃〉, **5**.|2, 1̃〉, **6**.|2, 2̃〉

For the two-particle basis, an additional number of states is added upon the ones from the one-particle basis in accordance with eqn (60). Thus, the two-particle contribution are (6 additional states, one separate vibrational quantum):

64 **7**.|1, 0̃; 2, 1〉, **8**.|1, 1̃; 2, 1〉, **9**.|1, 0̃; 2, 2〉, **10**.|2, 0̃; 1, 1〉, **11**.|2, 1̃; 1, 1〉, **12**.|2, 0̃; 1, 2〉

Note that *ν*_max_ = 2 was chosen to keep the matrix size small in this example. For calculations on real systems, it is advisable to use a larger value of *ν*_max_, as discussed earlier. For this example, with *N* = 2 and *ν*_max_ = 2, the Hamiltonian matrix will be of size 12 × 12. Each matrix element is defined analogously to Section 4.2. Here, however, the basis dimension exceeds the number of molecules because it includes botone- and two-particle states. Consequently, the matrix elements are defined as

65 *H*_***στ***_ ≡ 〈***σ***|*Ĥ*_FH_|***τ***〉

*i.e.*, “row *σ*, column *τ*” is the number obtained by sandwiching *Ĥ*_FH_ between 〈***σ***| and |***τ***〉 where ***σ*** and ***τ*** are the one- and two-particle basis states in eqn (63) and (64), respectively. Thus, with the basis ordering already defined, the first element in the Hamiltonian matrix is

66 *H*_11_ = 〈**1**|*Ĥ*_FH_|**1**〉 = 〈1, 0̃|*Ĥ*_FH_|1, 0̃〉

and so forth for the remaining terms.

#### 5.2.1 The diagonal entries

*H*_***σσ***_, in this example will take the form; for one-particle ↔ one-particle interactions |*n*, *ṽ*〉:

67 〈*n*, *ṽ*|*Ĥ*_FH_|*n*, *ṽ*〉 = *ε*_*n*_ + *ħω*_vib_*ṽ*

and for two-particle ↔ two-particle interactions |*n*, *ṽ*; *n*′, *v*′〉

68 〈*n*, *ṽ*; *n*′, *v*′|*Ĥ*_FH_|*n*, *ṽ*; *n*′, *v*′〉 = *ε*_*n*_ + *ħω*_vib_(*ṽ* + *v*′)

These diagonal elements take on relatively simple forms because, on the diagonal, the Hamiltonian returns the energy of the basis state itself. All terms in the Frenkel–Holstein Hamiltonian that mix different electronic sites or vibrational occupations (such as the excitonic coupling *J*^Coul^) either connect distinct basis states or vanish by orthogonality and therefore do not contribute when the bra and ket refer to the same basis state.^80^ Consequently, |*n*, *ṽ*〉 has energy *ε*_*n*_ + *ħω*_vib_*ṽ* while |*n*, *ṽ*; *n*′, *v*′〉 has energy *ε*_*n*_ + *ħω*_vib_(*ṽ* + *v*′).

#### 5.2.2 The off-diagonal terms

*H*_***στ***_, (***σ*** ≠ ***τ***) will consist of the exciton coupling, *J*^Coul^, multiplied with the appropriate vibrational overlap integrals. These overlap integrals are sometimes referred to as Franck–Condon overlaps, which quantify the overlap between vibrational wave functions on the excited state potential surface and the ground state potential surface.^80^ In the notation of Parson,^74^ and assuming equal curvatures of the harmonic potential surfaces and a dimensionless displacement of the potential surfaces quantified by the Huang Rhys factor, *S* = *λ*^2^, the overlap integrals for the ground state lowest vibrational level and the excited state lowest vibrational level is69
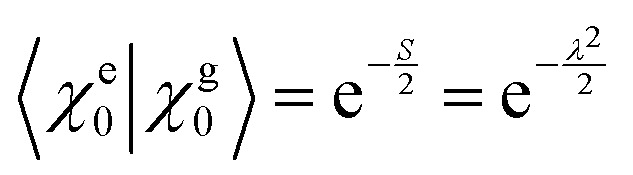
where *χ*^e^_0_ denotes the vibrational wavefunction in the excited state and *χ*^g^_0_ denotes the vibrational wavefunction in the ground state, both with vibrational quantum numbers 0 to indicate the zeroth vibrational level in each electronic state. In the notation used in this review, where the excited electronic state vibrational number is denoted with ∼, eqn (69) above can equivalently be written as

70 *F*_*ṽ*,*v*_ = 〈*ṽ*|*v*〉, (*ṽ*, *v* = 0, 1, 2, …)

71
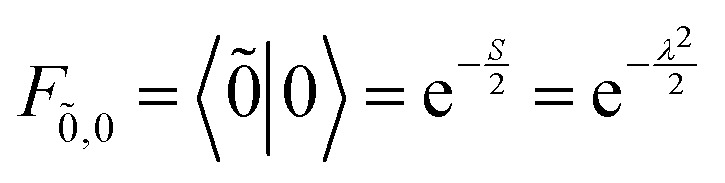


The overlap integrals for other combinations of vibrational levels can be built from 〈0|0〉 using the recursion formulas:^81^72
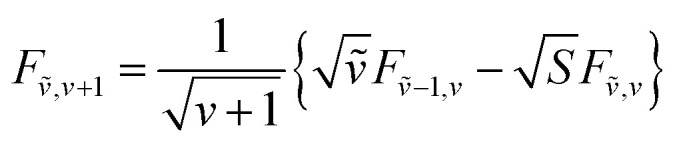
73
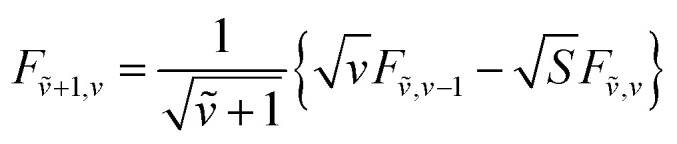


Note that any *F* with negative index, *e.g.*, *F*_−1,*v*_, is zero. Thus, for the overlap of the integral of the lowest vibrational level of the ground state with the *ṽ*th vibrational level in the electronic excited state74
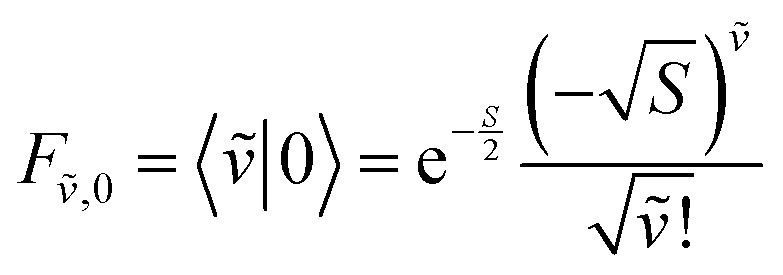


For the current example with *N* = 2 and *ν*_max_ = 2 the following overlap integrals will be needed
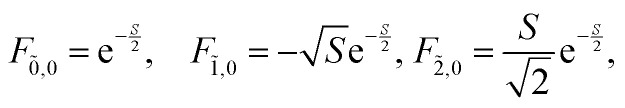

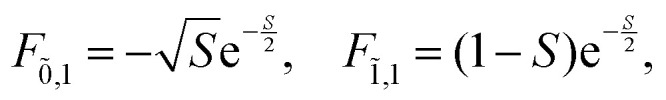

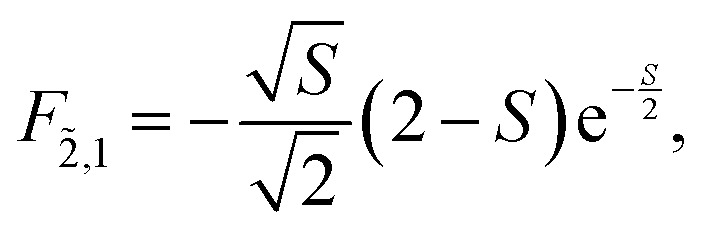
75
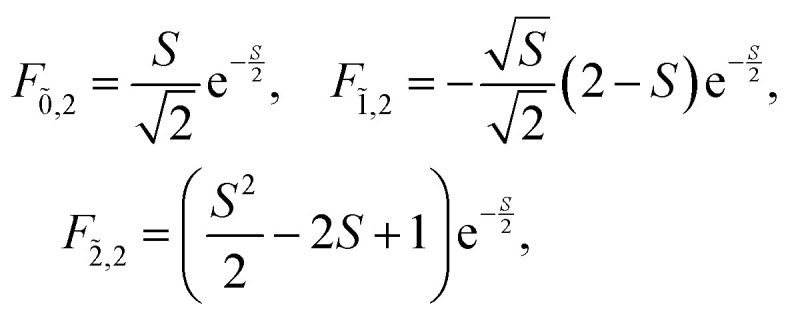
with the definitions of the overlap integrals, the off-diagonal terms can be defined according to the following equations. First, for the one-particle ↔ one-particle interactions:76
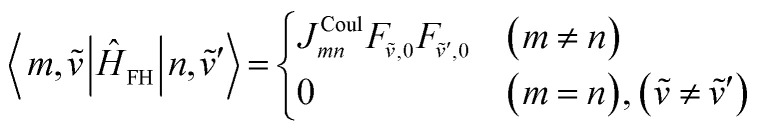


The off-diagonal terms are zero when (*m* = *n*) and (*ṽ* ≠ *ṽ*′) because different vibrational functions in the same harmonic potential are orthogonal to each other. It might not be obvious from the Hamiltonian in eqn (54) why the overlap factors appear in the off-diagonal terms. The relevant part of the Hamiltonian is the exciton transfer term *J*^Coul^_*mn*_(|*m*〉〈*n*| + |*n*〉〈*m*|), which changes which molecule is electronically excited, but does not (in the Condon approximation) move the nuclei. Thus, when evaluating the coupling between two one-particle vibronic states, one must project the nuclear state on each site from its initial potential surface to its final one. In other words, the site that gains the excitation goes from the ground-state potential to the excited-state potential, while the site that loses the excitation goes from the excited-state potential back to the ground-state potential. For example, when evaluating the matrix element 〈1, 2̃|*H̃*_FH_|2, 4̃′〉, the state |2, 4̃′〉 represents a state with 4 vibrational quanta in the excited state potential on molecule 2 while all other molecules have zero vibrational quanta in the ground state potential. When the system changes from the |2, 4̃′〉 state to the |1, 2̃〉 state, the vibrational wave function on molecule 2 changes to one describing zero quanta in the ground state potential, while the vibrational wave function on molecule 1 changes to one describing 2 vibrational quanta in the excited state potential. When evaluating matrix elements, these changes are indicated by the last two bra-kets in the following expression: 〈1, 2̃|*Ĥ*_FH_|2, 4̃′〉 = *J*_12_〈2̃|0〉_1_〈0|4̃〉_2_ where the 1 and 2 subscripts indicate the molecule on which the vibrational wave functions reside. These bra-kets are the vibrational overlap factors in eqn (76). Hence, each off-site matrix element is given by the electronic coupling, *J*^Coul^_*mn*_, multiplied by the appropriate product of vibrational overlaps.^80^

Next are the one-particle ↔ two-particle interactions:77
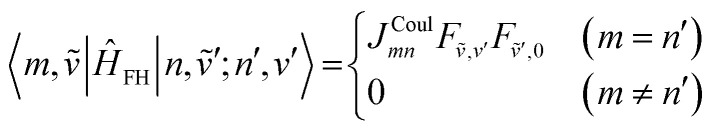
78
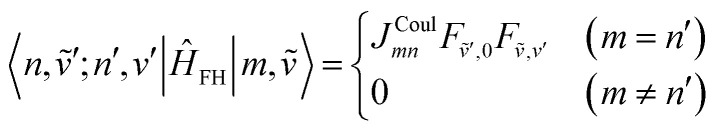
and finally, for the two-particle ↔ two-particle case:79
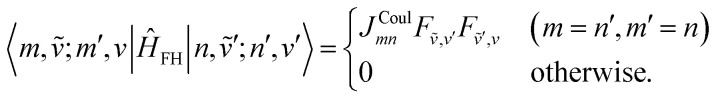


It should be noted that for aggregates with three or more sites (*N* ≥ 3) there is an additional two-particle ↔ two-particle contribution where the extra vibrational quantum remains on the same site in the bra and *k*:80
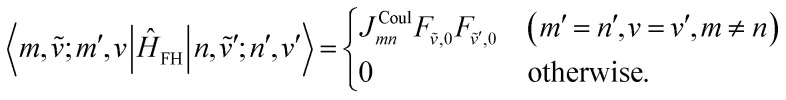


This term does not appear in the *N* = 2 example demonstrated here but becomes relevant when generalising the multiparticle basis to larger aggregates. With these terms in hand, the Hamiltonian matrix can be constructed and is shown below in block form where *J*^Coul^_12_ = *J*^Coul^_21_ = *J*.81
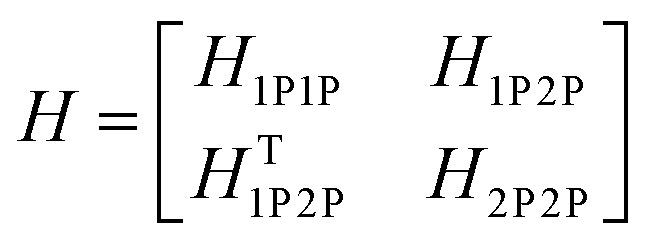
82
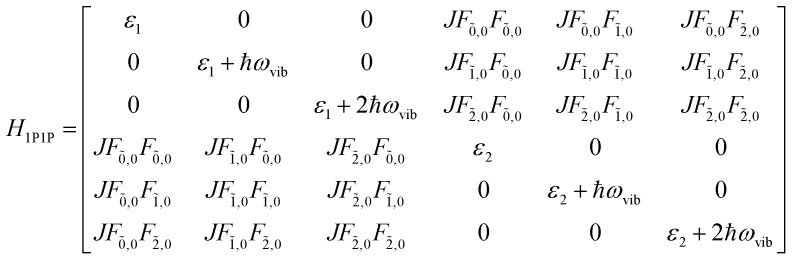
83
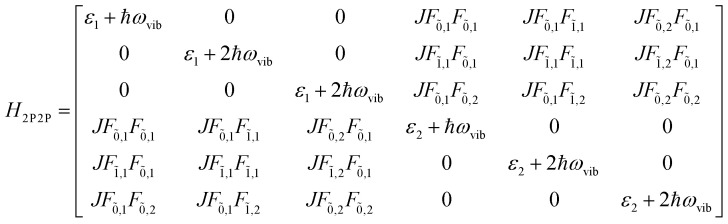
84
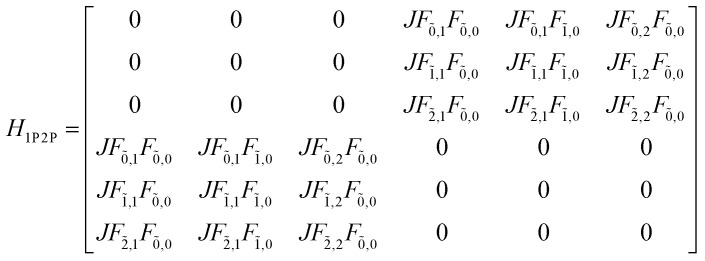


### 5.3 Simulation of absorbance spectra from the eigenvalues and eigenvectors obtained from diagonalization

Once the Hamiltonian matrix has been constructed, eigenvalues and eigenvectors can be obtained *via* diagonalization of *H* (as for the Frenkel Hamiltonian in Section 4.2). The full eigenvector and eigenvalue matrices are rarely written out because their size grows rapidly with the number of molecules, *N*, and total vibronic quanta, *ν*_max_. Instead, one specifies the *α*th eigenstate in the multiparticle basis (in this case the two particle-approximation) as85
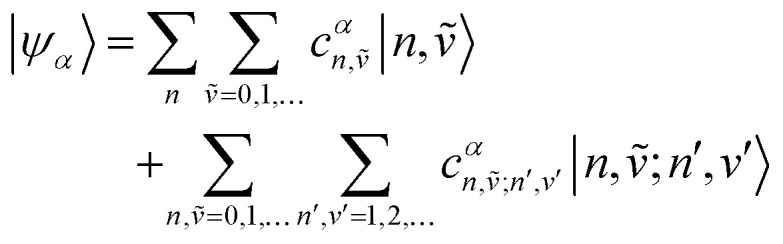
where *c*^*α*^_*i*_ are the eigenvector coefficients which are found by diagonalization of the Hamiltonian. Written out term by term for the example with *N* = 2 and *ν*_max_ = 2, eqn (85) is simply:86
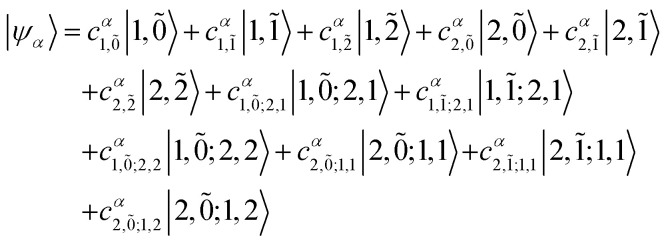


From the diagonalization, the obtained eigenvalues are the new hybrid state energies of the aggregate. Together with the simultaneously obtained eigenvector coefficients it is possible to simulate absorption and emission spectra.

#### 5.3.1 The absorption spectrum

As a function of *ω*, *A*(*ω*) is the sum over all transitions from the vibrationless ground state to all eigenstates |*ψ*_*α*_〉 according to87
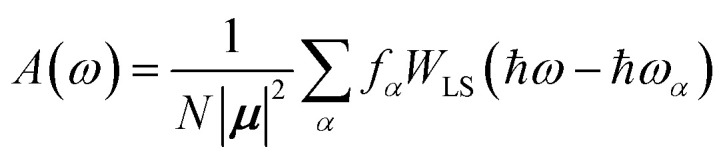


Here the division by *N* is to make the spectrum normalized to the number of molecules, *N*. Division by the monomer transition dipole moment magnitude |***μ***|^2^ is to make a reduced spectrum, which is dimensionless. *f*_*α*_ is the oscillator strength from the vibrationless ground state |*G*〉 to the *α*th eigenstate |*ψ*_*α*_〉 and is given by

88 *f*_*α*_ = *ħω*_*α*_|〈*ψ*_*α*_|*

<svg xmlns="http://www.w3.org/2000/svg" version="1.0" width="12.000000pt" height="16.000000pt" viewBox="0 0 12.000000 16.000000" preserveAspectRatio="xMidYMid meet"><metadata>
Created by potrace 1.16, written by Peter Selinger 2001-2019
</metadata><g transform="translate(1.000000,15.000000) scale(0.012500,-0.012500)" fill="currentColor" stroke="none"><path d="M480 1080 l0 -40 -40 0 -40 0 0 -40 0 -40 -40 0 -40 0 0 -40 0 -40 40 0 40 0 0 40 0 40 40 0 40 0 0 40 0 40 40 0 40 0 0 -40 0 -40 40 0 40 0 0 -40 0 -40 40 0 40 0 0 40 0 40 -40 0 -40 0 0 40 0 40 -40 0 -40 0 0 40 0 40 -40 0 -40 0 0 -40z M320 720 l0 -80 -40 0 -40 0 0 -120 0 -120 -40 0 -40 0 0 -120 0 -120 -40 0 -40 0 0 -80 0 -80 40 0 40 0 0 80 0 80 40 0 40 0 0 40 0 40 120 0 120 0 0 40 0 40 40 0 40 0 0 -40 0 -40 40 0 40 0 0 40 0 40 40 0 40 0 0 40 0 40 -40 0 -40 0 0 -40 0 -40 -40 0 -40 0 0 80 0 80 40 0 40 0 0 120 0 120 40 0 40 0 0 40 0 40 -40 0 -40 0 0 -40 0 -40 -40 0 -40 0 0 -120 0 -120 -40 0 -40 0 0 -80 0 -80 -120 0 -120 0 0 40 0 40 40 0 40 0 0 120 0 120 40 0 40 0 0 80 0 80 -40 0 -40 0 0 -80z"/></g></svg>


*|*G*〉|^2^

where ** is the transition dipole moment operator defined in eqn (35) but restated here for convenience:89
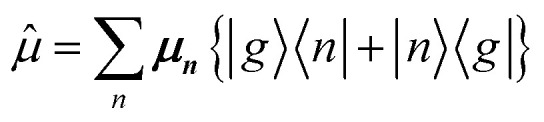
where |*g*〉 = *φ*^g^_1_*φ*^g^_2_…*φ*^g^_*N*_ = |*g*_1_, *g*_2_, …, *g*_*N*_〉 is the pure electronic aggregate ground state in which all molecules are in their electronic ground state and |*n*〉 = *φ*^g^_1_…*φ*^e^_*n*_…*φ*^g^_*N*_ = |*g*_1_, *g*_2_, …, *e*_*n*_, …, *g*_*N*_〉 is the site-localized electronic excited state on chromophore *n*. |*G*〉 in eqn (88) adds a vibrational specification. In the case of absorbance, and assuming no thermal activation of the ground state vibrational levels, the full vibronic ground state with zero vibrational quanta on every site in the ground-state potential is denoted as: |*G*〉 = |*g*; 0_1_, 0_2_, …, 0_*N*_〉. Finally, the last part of eqn (87), *W*_LS_(*ħω* − *ħω*_*α*_), is a homogeneous line shape function, usually of Lorentzian or Gaussian form. Thus, *A*(*ω*) in eqn (87) is, in simple terms, all the eigenvalues obtained from the diagonalization weighted by the transition dipole moments squared, which in turn are based on the eigenvector entries corresponding to each eigenvalue, and with a homogeneous broadeninfunction which converts the weighted transitions from single transition “lines” to bands centred at the eigenvalue. Note that the second part of the dipole moment operator 
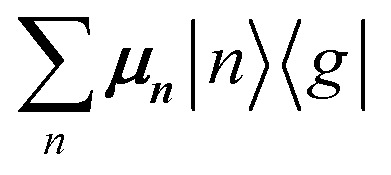
 creates an electronic excitation on molecule *n* from the ground electronic state |*g*〉 and is the only relevant term for absorption. The other term is zero in the case of absorption, but it is relevant for emission. With this in mind, and by inserting the *α*th eigenstate in the multiparticle basis of eqn (85) into eqn (86) one obtains the following expression:90
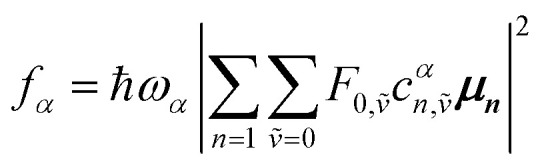


Note that within the Franck–Condon approximation and starting from the vibrationless ground state, the transition dipole creates a single electronic excitation while leaving the nuclear wavefunction unchanged. Written in the excited-state basis, this produces only one-particle states |*n*, *ṽ*〉 weighted by the Franck–Condon overlaps *F*_0,*ṽ*_ = 〈0|*ṽ*〉 as introduced in eqn (74). Consequently, in eqn (90) only the one-particle components of |*ψ*_*α*_〉 contribute and the two-particle contribution vanishes. However, of course the two-particle pieces matter indirectly through mixing in the Hamiltonian.

#### 5.3.2 Absorption example

As an illustrative example, consider a trimer (*N* = 3) in a head-to-tail configuration with *ν*_max_ = 4 (the geometry defined in Fig. 5a). In this case, with the two-particle approximation, the Hamiltonian matrix has a 75 × 75 dimension. Due to their large size, the matrices are presented in a separate MATLAB script (Vibronic_coupling_abs_em.m). The script follows the procedure of Section 5.2 including construction and subsequent diagonalization of the Hamiltonian matrix. With the obtained eigenvalue and eigenvector matrices the oscillator strength in eqn (90) of each hybrid state *α* can be calculated. As an example, for hybrid state *α* = 1:
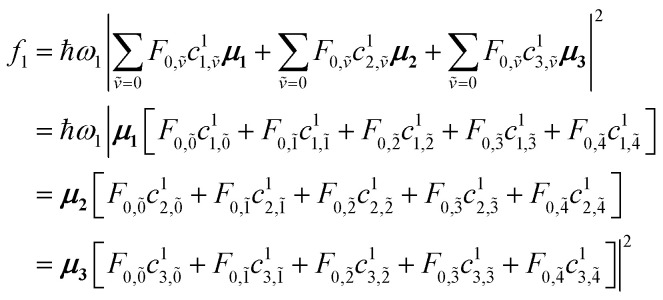
and so forth for every hybrid state where the eigenvector coefficients *C*^*α*=1^_*n*,*ṽ*_ are the one-particle elements of the first column in the eigenvector matrix *C* according to:91
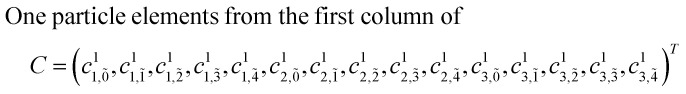


**Fig. 8:**
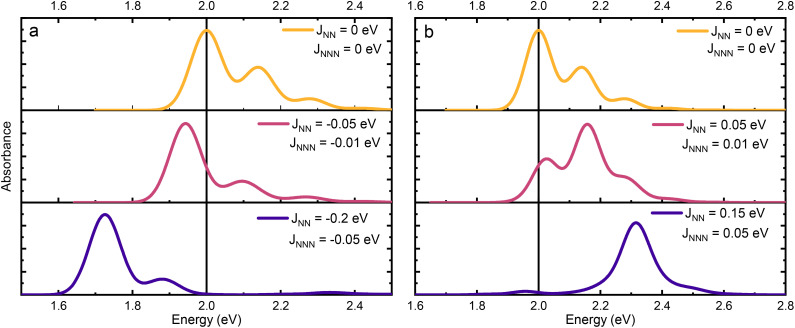
Simulated absorption spectra for a trimer (*N* = 3) with vibronic truncation *v*_max_ = 4, vibrational frequency *ω*_vib_ = 0.14 eV, and Huang–Rhys factor *S* = 0.5. (a) J-aggregate spectra for *J*^Coul^_NN/NNN_ < 0, starting with *J*^Coul^_NN/NNN_ = 0 at the top and increasing |*J*^Coul^_NN/NNN_| downward. (b) H-aggregate spectra for *J*^Coul^_NN/NNN_ > 0 with coupling magnitudes similar to panel (a). The line shape is a Gaussian function with FWHM = 0.1 eV.

And the remaining 60 elements in each column are the two-particle elements, which, as previously mentioned, only contributes indirectly through mixing in the Hamiltonian. It is assumed, as it was for the example with the Frenkel Hamiltonian in Section 4.2 with the head-to-tail configuration, that the direction of all monomer transition dipole moments is the same and with equal magnitude |***μ***_1_| = |***μ***_2_| = |***μ***_3_| = |***μ***|. The direction can be arbitrarily chosen to be the *x*-axis; ***μ***_***n***_ = |***μ***|***x̂***, where ***x̂*** is a unit vector along the *x*-axis. With the direction defined, the oscillator strength of all hybrid states can be calculated, and the reduced absorption spectra can be produced according to eqn (87) and it is presented in Fig. 8a. Fig. 8a further shows the simulated absorption spectra of the same system with *N* = 3 and head to tail orientation (J-aggregate) but with stronger coupling. As expected, as the coupling strength increases the spectra progressively shifts to lower energy. Importantly, the ratio of the 0-0 and 0-1 absorption bands increases with the coupling strength. This behaviour is typical for J-aggregates, and this can in fact be used to extract coupling strengths from experimentally obtained steady state spectra. This will be described in greater detail in Section 5.5. Fig. 8b further shows another example with the same coupling strength magnitude, but flipped in sign, so that it is now an H-aggregate. Consequently, we see the opposite trend with a spectral shift to higher energy and loss of intensity of the 0-0 peak relative to the 0-1 peak, resulting in a decrease in the 0-0/0-1 ratio. However, something interesting occurs as the coupling strength is further increased. An absorption peak with small intensity becomes discernible at lower energy relative to the uncoupled system. This occurs due to an intermolecular version of Herzberg–Teller coupling, which can be observed for sufficiently strong coupling strengths. Herzberg–Teller coupling is a type of intensity borrowing mechanism in which a formally forbidden electronic transition acquires weak oscillator strength *via* vibronic mixing.^82–84^ Note however, that a weak, lower-energy feature in an aggregate spectrum does not always mean that it is an H-aggregate and that the feature originates from Herzberg–Teller coupling. There are also other reasons for lower energy features such as charge transfer interactions (*vide infra*) and/or simultaneous J-aggregate coupling with a different neighbouring chromophore in some other direction, which will be discussed in more detail in Section 7.1.

#### 5.3.3 Example comparison with experimental data

**Fig. 9:**
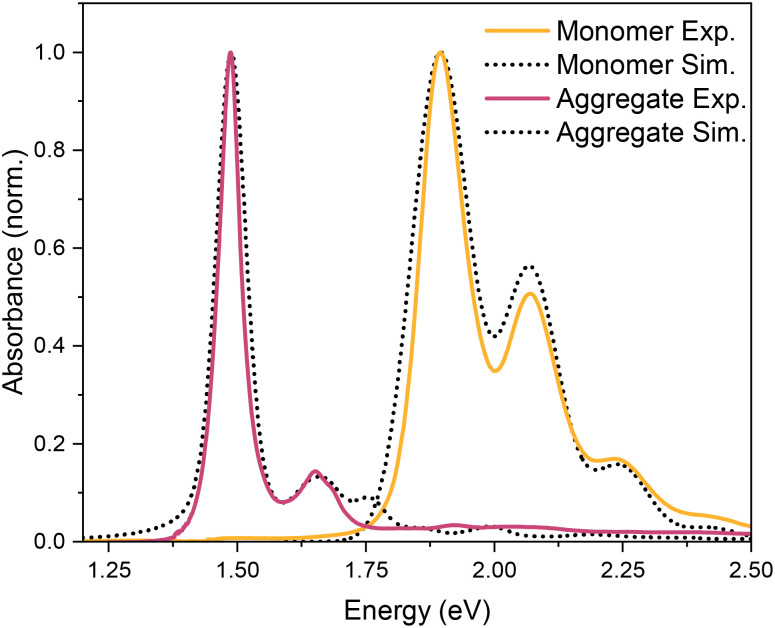
Quaterrylene monomer (in toluene) and aggregate (in 1,1,2,2-tetrachloroethane) experimental spectra (solid lines) measured in ref. 7, along with the theoretical spectrum (dotted line). The parameters used for the theoretical aggregate spectra were *N* = 10, *λ*^2^ = 0.51, *ω*_vib_ = 0.175 eV, *J*^Coul^_NN_ = −0.2 eV and *J*^Coul^_NNN_ = −0.045 eV. The line shape is a Voigt function with Gaussian FWHM = 0.065 eV and Lorentzian FWHM = 0.085 eV. For the monomer, *J*^Coul^_NN/NNN_ = 0 and the line shape is a Gaussian function with FWHM = 0.1 eV.

Molecules such as derivatives of quaterrylene can spontaneously form J-aggregates in certain solvents. One example is bayrac-alkylated quaterrylene for which the experimentally obtained monomeric (in toluene) and aggregate (in 1,1,2,2-tetrachloroethane) absorption spectra are presented in Fig. 9.^7^Fig. 9 further displays the theoretically obtained absorption spectrum calculated using the vibronic coupling theory discussed above. From the monomer spectrum the Huang–Rhys factor, *λ*^2^, was determined to be 0.51 and *ω*_vib_ = 0.175 eV. A reasonable match was obtained with *N* = 10 and with nearest and next-nearest coupling of *J*^Coul^_NN_ = −0.2 eV and *J*^Coul^_NNN_ = −0.045 eV, respectively. The experimental and theoretical spectra do not coincide perfectly, which is not entirely expected given that the sample likely contains a distribution of aggregates with different *N* and varying degrees of disorder.

### 5.4 Simulation of emission spectra from the eigenvalues and eigenvectors obtained from diagonalization

The emission spectrum, in turn, can be calculated according to92
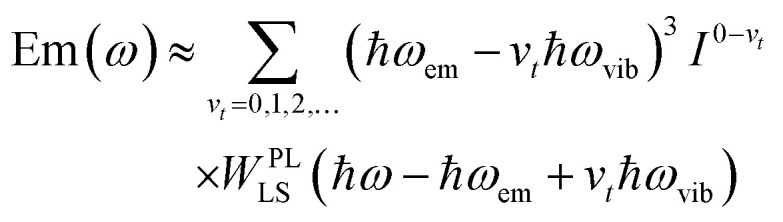
where it is assumed that Kasha's rule^85^ applies and emission occurs from the lowest energy hybrid state |*ψ*_*α*_〉 = |*ψ*_em_〉 with energy *ħω*_em_. This is a good approximation when the energy separation between |*ψ*_em_〉 and higher hybrid states is several *k*_B_*T* (so that thermal repopulation is negligible) and nonradiative relaxation within the exciton manifold is much faster than radiative decay. In many molecular aggregates, however, several hybrid states may be thermally accessible at room temperature, which will then require a Boltzmann average over emitting states.^86,87^*W*^PL^_LS_(*ħω* − *ħω*_em_ − *v*_*t*_*ħω*_vib_) is a normalised line shape, and the (*ħω*_em_ − *v*_*t*_*ħω*_vib_)^3^ factor is the dipole radiation prefactor for spontaneous emission. The *I*^0–0^ emission line strength (*v*_*t*_ = 0) is given by93
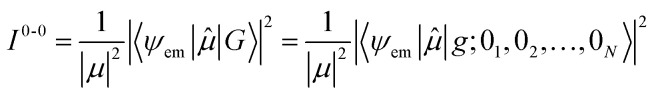
and is the transition from the emitting eigenstate |*ψ*_em_〉 to the full vibronic ground state with zero vibrational quanta on every site in the ground-state potential, |*G*〉, as introduced in the absorption section. The expression is similar to the oscillator strength in eqn (88) since both equations involve transitions to or from |*G*〉 where every monomer in the system is in the electronic and vibrational ground state. The remaining *I*^0−*v*_*t*_^ emission line strengths are given by94
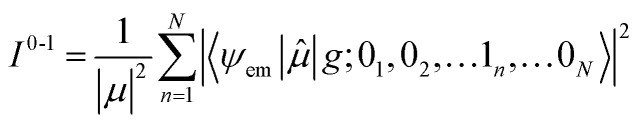
95
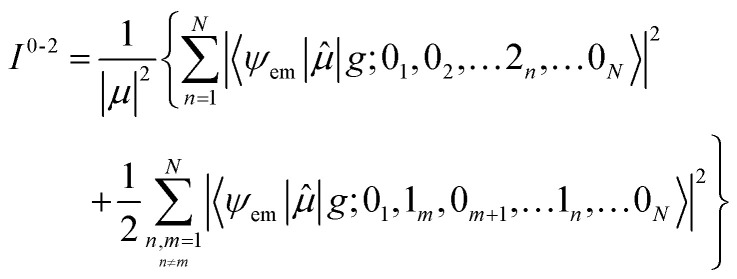
and so forth for *I*^0-3^ and higher terms. Note that division by |*μ*|^2^ is to obtain a reduced, dimensionless line strength and the ½ factor in eqn (95) is there to prevent double counting (*e.g.*, when *n* = 1 and *m* = 2 and then also when *m* = 1 and *n* = 2). In eqn (94), the term |*g*; 0_1_, 0_2_, …1_*n*_, …0_*N*_〉 indicate that the transition ends in a manifold of degenerate, orthogonal states with one vibrational quantum somewhere in the ground electronic manifold. Since there are *N* distinct final states in this case, the intensity of each possible terminal state must be accounted for, which is why the summation appears in eqn (94) but is absent in eqn (93). As an example, for *N* = 2, eqn (94) becomes96
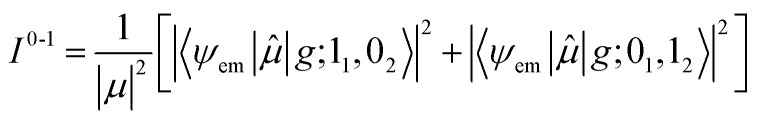


In the case of *I*^0-2^ in eqn (95), there are two summations because two vibrational quanta in the ground electronic state can be realized in two distinct ways. In the first sum, both vibrational quanta are on the same molecule. In the second sum, there is one vibrational quantum on two distinct molecules giving a total of two vibrational quanta.

Similar to the absorption case, by inserting the multiparticle expansion of |*ψ*_*α*_〉 (eqn (85)) and using the Condon approximation, the emission line strengths can be written as97
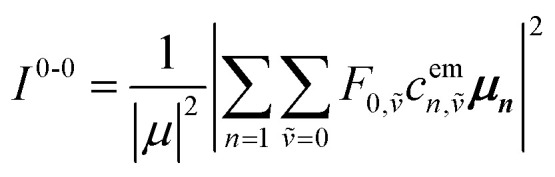
98
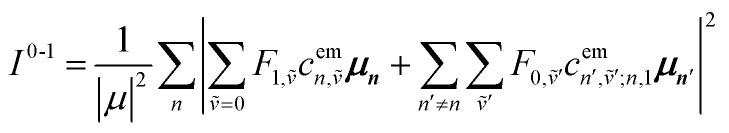
99
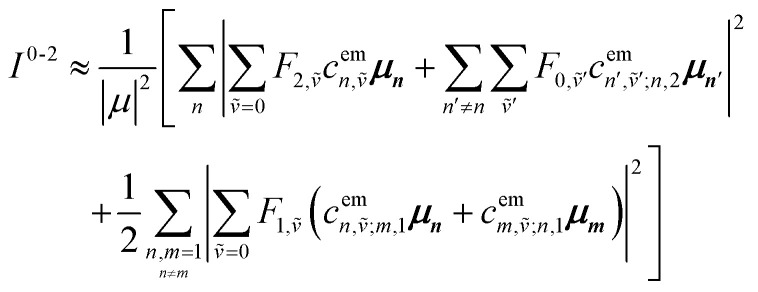
where *c*^em^_(*n*,*ṽ*)_, 
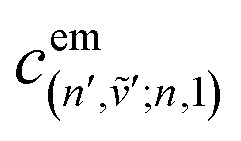
, *etc.*, are the eigenvector coefficients of the emitting eigenstate, *ψ*_em_. Note that *I*^0-0^ and *I*^0-1^ as written in eqn (97) and (98), respectively, are exact within the two-particle approximation. However, for *I*^0-2^ in eqn (99), a component containing three particle coefficients is neglected and the expression is thus an approximation. However, the three-particle contribution is usually small for *I*^0-2^. For *I*^0-3^ and higher terms there are additional components that involve three particle coefficients, which are zero in the two-particle approximation. Consequently, higher terms require *t* application of higher order multi-particle approximations to yield fully accurate results. However, at relatively small coupling strengths one can achieve reasonable accuracy without using higher order multi-particle states.^44,88,89^

#### 5.4.1 Emission example

**Fig. 10:**
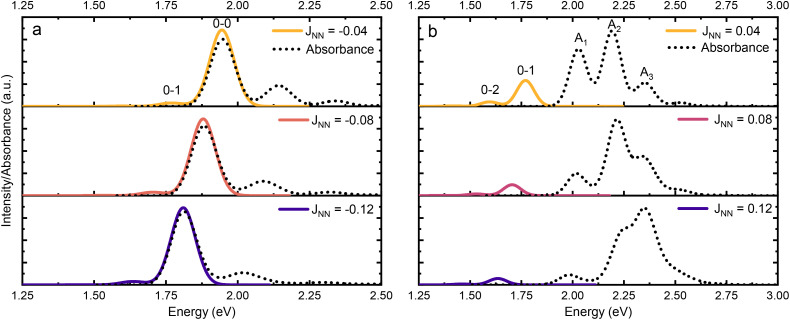
Simulated absorption and emission spectra for *N* = 10 aggregate with vibronic truncation *v*_max_ = 7, vibrational frequency *ω*_vib_ = 0.175 eV, and Huang–Rhys factor *S* = 0.51 with open boundary conditions. (a) J-aggregate spectra for *J*^Coul^ < 0, starting with small coupling strength in the top and increasing |*J*^Coul^| downward. (b) H-aggregate spectra for *J*^Coul^ > 0 with coupling magnitudes matched to panel (a).

For the emission, a system of *N* = 10 with nearest neighbour coupling and open boundary conditions is simulated. The total number of vibrations is *ν*_max_ = 7, the transition energy of the uncoupled monomers, *ε*_*n*_ = 2 eV, the vibrational frequency *ħω*_vib_ = 0.175 eV and the Huang–Rhys factor *λ*^2^ = 0.51. The emission spectra are presented for varying coupling magnitudes for *J*^Coul^ < 0 (J-aggregate) and *J*^Coul^ > 0 (H-aggregate) in Fig. 10a and b, respectively. Additionally, the emission spectra are plotted together with the corresponding absorption spectra with the same parameters to fully demonstrate the spectral shifts.

The simulated spectra in Fig. 10 corresponds to ideal aggregates. This means that there is no energetic or structural disorder, and, in addition, temperature effects are neglected. In the strict limit of perfectly ordered H-aggregate with periodic boundary conditions, the 0-0 emission is symmetry-forbidden and therefore vanishes completely as shown in Fig. 10b. However, real aggregates are finite and disordered to some degree, and a small 0-0 intensity can thus be observed in experimental spectra. In addition, the 0-0 peak can also arise when states above the lowest energy excited state in H-aggregates are thermally populated and emit.^90^ Note that in these simulations the 0-0 absorption and emission spectra coincide, *i.e.* there is no intrinsic Stokes shift. This follows from the idealized Frenkel–Holstein model used here, which assumes identical harmonic ground- and excited-state potentials and neglects environmental reorganization. As for the 0-1 and higher order vibronic peaks, vibronic coupling results in non-zero intensities that decrease with increasing coupling strength, which can also be observed in Fig. 10. In the following section, a brief discussion regarding the role of the mentioned periodic and open boundary conditions and the influence of disorder is presented.

#### 5.4.2 Open and periodic boundary conditions

In the context of exciton coupling, periodic boundary conditions means that the aggregate is treated as if its ends were joined to form a ring. The system then has exact translational symmetry, and the exciton states are fully delocalized wave-like states that extend over the entire aggregate. The opposite case is open boundary conditions, where the aggregate has two distinct ends and the exciton wavefunctions form standing waves. The simulated emission and absorption spectra in Fig. 10 were generated using open boundary conditions. For finite aggregates of relatively small *N*, this choice is generally more realistic, because actual molecular assemblies always have a finite length with end effects.^44,89^

In an ideal H-aggregate, the lowest energy excited state has eigenvector coefficients that alternate in sign from one molecule to the next. Under open boundary conditions these amplitudes form a standing-wave envelope across the chain. For even *N*, the positive and negative contributions to the transition dipole moment cancel almost exactly. Thus, the total transition dipole moment, and by extension the 0-0 line, is strongly suppressed as shown Fig. 10 for *N* = 10. For odd *N*, this cancellation is slightly imperfect because one side of the standing wave is not exactly mirrored by the other, leading to a weak but non-zero 0-0 intensity. Imposing periodic boundary conditions restores perfect destructive interference for any *N*, and in the ideal H-aggregate limit the 0-0 emission is strictly zero. However, for very large *N* the choice of open or periodic boundary conditions only has a minor influence on the observed spectrum. The concept of open and periodic boundary conditions is nonetheless very important. Several analytical relations from vibronic coupling theory (for example expressions for band intensities and their ratios in H- and J-aggregates, which will be discussed in Section 5.5) are derived under the assumption of periodic boundary conditions.

### 5.5 Reversing the procedure – extracting information from absorption and emission spectra

Up to this point it has been shown that using vibronic coupling, new exciton states can be calculated, and absorption and emission spectra can be simulated by solving the Holstein Hamiltonian. In addition, the vibronic coupling framework can be used to extract quantities such as the exciton coherence length or the exciton coupling strength, *J*^Coul^, by analysing experimental absorption and emission spectra.

#### 5.5.1 Coherence from emission spectra

Emission spectra can give information about how many chromophores share a coherent excitation. This can be understood by comparing eqn (97) and (98) which describe the emission line strengths *I*^0-0^ and *I*^0-1^, respectively. Eqn (97) is the square of a sum over molecules. Writing 
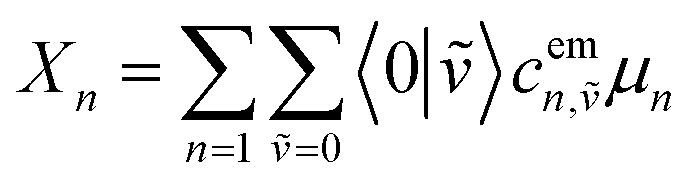
 one has 
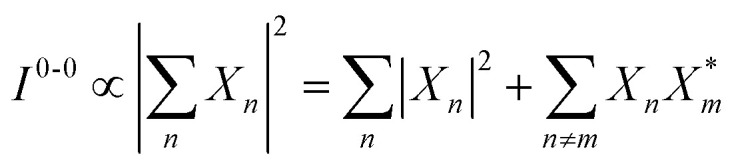
 where the asterisk means complex conjugate. The extra terms 
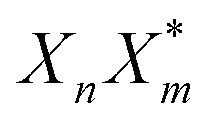
 represent mutual reinforcement between different molecules. When the emitting excited state is coherently shared across many molecules so that their contributions *X*_*n*_ point in the same “direction” (*i.e.*, are similar and combine in the same way), these terms add rather than cancel, and the 0-0 intensity grows with the number of molecules that participate coherently (the coherence number *N*_coh_). If thermal fluctuations or static inhomogeneities make different molecules in the coupled system contribute in mismatched ways, the reinforcement terms largely cancel and the 0-0 band is suppressed. In this sense, 0-0 directly reflects exciton coherence. By contrast, eqn (98) for 0-1 is a sum over distinct final states in which the single ground-state vibrational quantum resides on a specific molecule *n*. Final states with the vibrational quantum on different molecules are orthogonal, so inter-molecule reinforcement does not survive when forming the total intensity; what remains is a sum of squares over molecules, with only a local one-particle/two-particle mixing within each absolute value. As a result, 0-1 is largely insensitive to how many molecules share the excitation coherently and do not display the coherent enhancement characteristic of 0-0. These structures explain the different photoluminescence fingerprints of H- and J-aggregates. In an ideal J-aggregate the emitting excited state is distributed nearly uniformly over many molecules, the terms in eqn (97) then add coherently so that *I*^0-0^_aggregate_ scales with the coherence number, whereas *I*^0-1^_aggregate_ does not. This leads to the ratio rule100
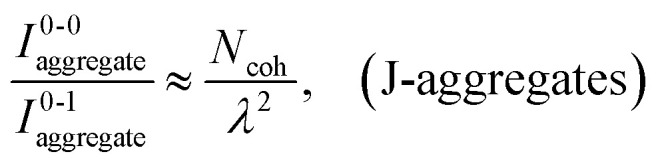


In an ideal H-aggregate, symmetry causes the molecular contributions in eqn (97) to cancel, making 0-0 forbidden in the disorder free case while the vibronic sidebands such as 0-1 remain allowed. It is thus possible to have emission even in an ideal H-aggregate, however, only starting from the 0-1 state as demonstrated in Fig. 10. The intensity of the 0-1 band will however diminish with increasing exciton coupling strength.

In the context of exciton coupling, “disorder” refers to static, molecule-to-molecule variations in transition energies, intermolecular couplings, and/or transition-dipole orientations that are frozen on the emission timescale. Another example of disorder are the end sites of an aggregate, which break translational symmetry and partially localize the excited state, slightly modifying the intensities compared to the periodic, disorder-free case. These kinds of disorder shorten coherence and breaks perfect translational symmetry. In J-aggregates it reduces *N*_coh_ and weans 0-0 relative to 0-1. In H-aggregates it can activate 0-0 (previously symmetry-forbidden) so the ratio *I*^0-0^/*I*^0-1^ becomes a sensitive indicator of the degree of disorder even in H-aggregates (even though the ratio rule in eqn (100) is only valid for J-aggregates).

#### 5.5.2 Linewidth narrowing

The coherence number can also be obtained without accounting for vibronic coupling as deduced by Knapp who showed that when the Coulomb coupling is sufficiently large, the absorption linewidth of J-aggregates is reduced by 
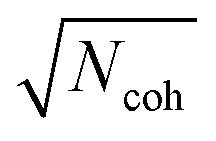
.^91^ This narrowing arises because the optically bright excited state is delocalized over *N*_coh_ molecules when the individual molecules experience slightly different local transition-energy shifts. For an isolated monomer, the observed linewidth directly reflects the distribution of local transition energies associated with its environment. In an exciton-coupled aggregate, the optical transition probes an excitation that is distributed over several molecules, so the effective transition energy corresponds to an average over the local site energies within the delocalization length. If these site-energy shifts are uncorrelated from molecule to molecule, the resulting variance is reduced by a factor of 1/*N*_coh_, giving a linewidth reduction on the order of 
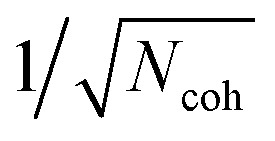
. However, if the energetic shifts are correlated across many neighboring molecules, this averaging mechanism is suppressed, and the aggregate linewidth approaches the monomer linewidth.^91^ Line narrowing in the case of emission is a less reliable indicator of the coherence number. This is because line narrowing may simply occur due to the lowest energy state in a slightly disordered aggregate being the emitting state. Thus, the ratio rule from vibronic coupling theory is a more robust method of estimating the coherence number and disorder. However, note that the ratio rule in emission is naturally sensitive to so called second order inner-filter effects where the overlap of the 0-0 absorption and emission can lead to a drop in the emission ratio of 0-0 and 0-1 peaks due to reabsorption of the emission. The inner-filter effect can be quite prominent in aggregates and must be eliminated or mitigated for accurate usage of the ratio equation.

#### 5.5.3 Exciton coupling strength from absorption spectra

Using vibronic coupling theory, valuable information can also be extracted from the relative ratio of the absorption peaks. By experimentally determining the ratio of the 0-0 and 0-1 absorption transitions of an aggregate (and having parameters such as the Huang–Rhys factor and *ω*_vib_ from the monomer absorption spectrum) it is possible to calculate the nearest neighbour exciton coupling *J*^Coul^_NN_ using the following equation:^12^101
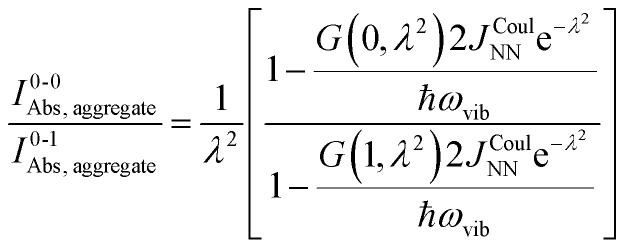
where *J*^Coul^_NN_ should be negative for J-aggregates and positive for H-aggregates and where the vibrational function, *G*(*ν*_*t*_; *λ*^2^), can be calculated as102
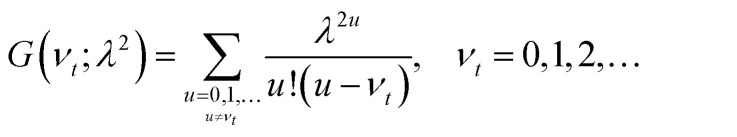


Here, *ν*_*t*_ indexes the observed vibronic band (0-0, 0-1, …). The sum in *G*(*ν*_*t*_; *λ*^2^) runs over *u* = 0, 1, 2, …, with *u* ≠ *ν*_*t*_ where *u* is the monomer vibronic (vibrational quantum) level index. In practice the series is truncated once the partial sums converge. Note that the ratio formula in eqn (101) is strictly valid for aggregates with periodic boundary conditions. For very large aggregates, edge effects become negligible, and the result is a good approximation even with open boundary conditions. For dimers, the boundary conditions are effectively irrelevant. Note that in the dimer case, the factor of 2 in eqn (101) reduces to 1, since only a single nearest-neighbour interaction is present. In the case of trimers and similar smaller aggregates open boundary conditions introduce edge effects that can lead to slight deviations from the ratio formula.^92^

As a final note, the multiparticle basis set that has been described here accurately describes the excitonic eigenstates in the weak to intermediate coupling regime. The two-particle approximation is usually a good enough approximation in this regime. However, stronger coupling might require the inclusion of three particle- or higher states.^93^ In the strong coupling regime it might also be beneficial to work with another basis such as the exciton–phonon basis.^29,94^ Additional information about vibronic coupling can be found in the review by Hestand and Spano,^12^ which covers the theory in detail and includes discussion on for instance the coupling of multiple vibronic modes,^95^ and the inclusion of disorder and temperature effects in the calculations,^86^ which is not treated here. A related but distinct framework is the Kühn–Renger–May approach, which also treats exciton-vibrational coupling.^96,97^ In this formulation, one or a few effective vibrational modes are retained explicitly in the system Hamiltonian, while the remaining vibrations are represented as a phonon bath. The Kühn–Renger–May framework is therefore particularly useful for describing environmental effects such as relaxation, dephasing, and spectral broadening, and more generally for capturing how coupling to the surroundings influences the photophysics of molecular aggregates. Related Kühn–Renger–May-inspired implementations have been applied to cyanine aggregates and DNA-templated dye assemblies, where fitting of absorption and circular dichroism spectra has been used to extract structural parameters such as intermolecular separations and relative dye orientations.^98–101^

## 6. Charge transfer interactions

In the preceding sections, the aggregate photophysics were modelled first within a purely Frenkel and subsequently a Frenkel–Holstein picture. In both cases the electronically excited manifold was spanned solely by neutral, localized excitations coupled by a Coulombic resonance interaction and, in the case of the Frenkel–Holstein picture, modulated by intramolecular vibrational modes. This description is appropriate when the chromophores are separated far enough, so that their frontier orbitals (HOMO and LUMO) do not overlap appreciably. For many π-stacked aggregates of interest, however, the centre-to-centre separation between monomers is only on the order of 3–5 Å. At such short distances the electronic wavefunctions on neighbouring molecules overlap, and the simple picture of a purely Coulombic exciton coupling becomes incomplete. In addition to the neutral, localized, Frenkel configurations, one must then include configurations in which the electron is localized on a different chromophore than the hole. These so-called charge-transfer (CT) configurations formally correspond to a positively charged (cationic) site and a negatively charged (anionic) site on different molecules. CT states are sometimes also referred to as charge separated states when there is considerable physical separation of the electron and hole, whereas CT is more commonly used when the charges are in proximity. Importantly, even when such CT states lie several hundred meV above the lowest neutral excited state, their admixture into the optical excitations can substantially affect both the excited state energies and the effective intermolecular coupling.^102,103^ Physically, the appearance of CT character introduces a new class of short-range interactions that are not captured by the Coulombic coupling. The total excitonic coupling instead contains both a long-range Coulomb term and a short-range CT-mediated term, which can interfere constructively or destructively depending on aggregate geometry and electronic structure. To incorporate these effects, the purely Frenkel Hamiltonian introduced in Section 4.2 can be extended by enlarging the electronic basis to include CT configurations. The possibility of mixing effects with CT states was proposed by Lyons in 1957.^104^ A detailed theoretical investigation was subsequently done by Merrifield in 1961.^25^ Merrifield's model for Frenkel–CT mixing considers electron and hole transfer between neighbouring monomers, described by one-electron and one-hole hopping integrals, *t*_e_ and *t*_h_, respectively, defined as

103 *t*_e_ ≡ 〈*ϕ*^A^_LUMO_|*ĥ*|*ϕ*^B^_LUMO_〉

104 *t*_h_ ≡ −〈*ϕ*^A^_HOMO_|*ĥ*|*ϕ*^B^_HOMO_〉

where *ϕ*^A^_LUMO(HOMO)_ is the LUMO (HOMO wavefunction on molecule A or B and *ĥ* is the single electron Hamiltonian operator. The Merrifield Hamiltonian, *Ĥ*_M_, can be split into a Frenkel part, *Ĥ*_F_, a CT part, *Ĥ*_CT_, and a term at mixes Frenkel and CT states, *Ĥ*_F–CT_, according to

105 *Ĥ*_M_ = *Ĥ*_F_ + *Ĥ*_CT_ + *Ĥ*_F–CT_

where *Ĥ*_F_ is the Frenkel Hamiltonian introduced in Section 4.2.106
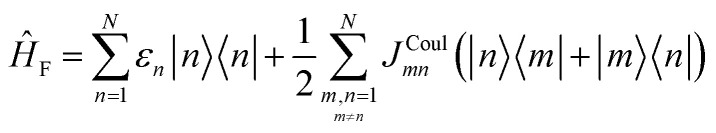


The transfer of electron and hole between neibouring molecules is accounted for by *Ĥ*_CT_:107
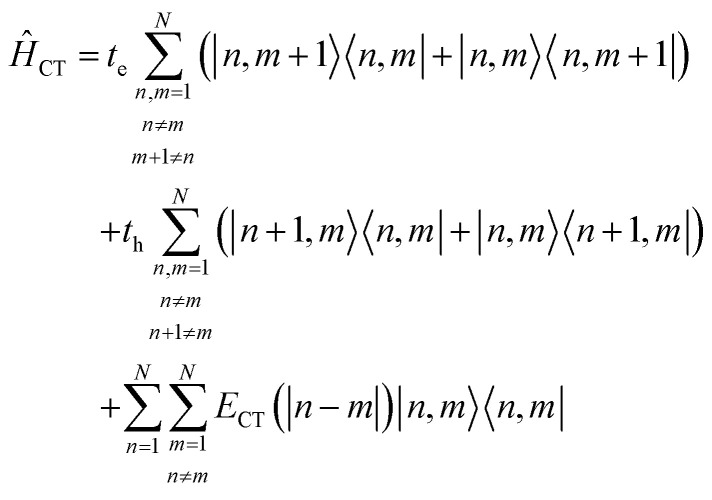
Here |*n*, *m*〉 represents the state where a hole resides on molecular site *n* (molecule *n*) on the aggregate, and an electron resides on molecular site *m*. Note that this CT basis is not the same as the “two-particle” vibronic basis used in the vibronic coupling section. In that case, “two-particle” refers to an exciton plus a local vibrational excitation, not a separated electron–hole pair.

The first term in eqn (107), proportional to *t*_e_, describes motion of the electron within the CT manifold, with |*n*, *m* + 1〉〈*n*, *m*| moving an electron from site *m* to site *m* + 1 and the term |*n*, *m*〉〈*n*, *m* + 1| moving the electron from site *m* + 1 to site *m*. The second bracket, proportional to *t*_h_, similarly describes motion of the hole, with |*n* + 1, *m*〉〈*n*, *m*| moving a hole from site *n* to site *n* + 1 and |*n*, *m*〉〈*n* + 1, *m*| moving a hole from site *n* + 1 to site *n*. Also note that the summations in the first and second terms avoid states where *n* = *m*, because these states correspond to the Frenkel excitons where the electron and hole reside on the same site. The Frenkel excitons can couple to the CT states through *t*_e_ and *t*_h_, but such terms are contained in *H*_F–CT_. The last sum assigns an energy, *E*_CT_ (*n* − *m*), to each CT configuration with the electron and hole residing on sites *m* and *n* respectively.

The final term in the Merrifield Hamiltonian is the Frenkel–CT mixing term, *Ĥ*_F–CT_, which couples a local neutral excitation on site *n* to CT configurations, in which either the electron or the hole has been transferred to a neighbouring site.108
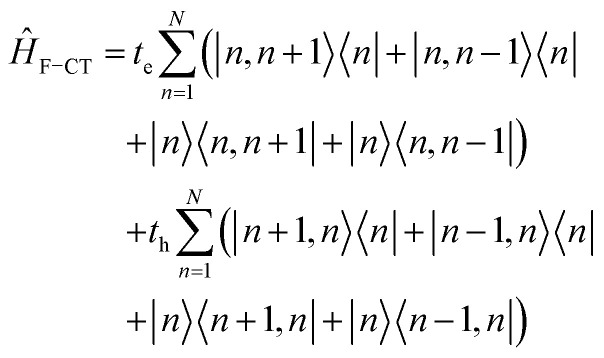


Note that vibronic coupling has not yet been considered in *Ĥ*_M_. The inclusion of vibronic coupling is discussed first in Section 6.2. Additionally, it should be mentioned that the Merrifield Hamiltonian can also be expressed in using creation and annihilation operators as described by for instance Agranovich and Bassani.^75^

### 6.1 Example calculation

As an example, with *N* = 3 the basis ordering will take the form:

Frenkel states (3 states) of the form |*n*〉 = |1〉, |2〉, …, |*N*〉:

109 **1**.|1〉 = |*φ*^e^_1_*φ*^g^_2_*φ*^g^_3_〉, **2**.|2〉 = |*φ*^g^_1_*φ*^e^_2_*φ*^g^_3_〉, **3**.|3〉 = |*φ*^g^_1_*φ*^g^_2_*φ*^e^_3_〉

CT states (6 states) of the form |*n*, *m*〉 where the first index, *n*, indicates the position of the cation (hole) and the second index indicates the position, *m*, of the anion (electron):110
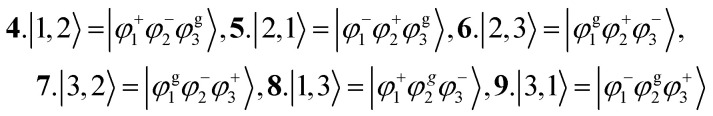


The Frenkel states are the same as in the pure Frenkel case described in Section 4.2. The CT states follow the same logic where *φ*^e/g/+/−^_*n*_ are the electronic wavefunctions of molecule *n* in the ground (g), excited (e), cationic (+) or anionic (−) states.

In this ordering, the first three basis states are pure Frenkel excitons and the remaining six are CT configurations. Among the CT states, states **4–7** correspond to nearest-neighbour CT (|*n* − *m*| = 1), while **8** and **9** correspond to next-nearest-neighbour CT (|*n* − *m*| = 2). For this example, with *N* = 3 and the above basis ordering, the electronic Hamiltonian matrix will be of size 9 × 9. Each matrix element is defined in the same way as in Section 5 according to

111 *H*_***στ***_ ≡ 〈***σ***|*Ĥ*_M_|*τ*〉

*i.e.*, “row *σ*, column *τ*” is the number obtained by sandwiching the Merrifield Hamiltonian, *Ĥ*_M_, between 〈***σ***| and |***τ***〉 where ***σ*** and ***τ*** are the Frenkel and CT basis states defined and ordered in eqn (109) and (110), respectively.

#### 6.1.1 Diagonal terms

The diagonal entries consist of the energies of the pure Frenkel and CT states. First are the Frenkel energies, *ε*_*n*_, of the individual monomers in the relevant environment:

112 〈*n*|*Ĥ*_M_|*n*〉 = *ε*_*n*_

The CT energies depend on the electron–hole separation, *n* − *m*. With the above defined basis, the diagonal entries will be the energy of the CT state, *E*_CT_ (|*n* − *m*|), according to:

113 〈*n*, *m*|*Ĥ*_M_|*n*, *m*〉 = *E*_CT_ (|*n* − *m*|)

In the current *N* = 3 example, there are two distinct diagonal CT energies, *E*_CT_ (1) and *E*_CT_ (2), corresponding to nearest-neighbour (|*n* − *m*| = 1) and next-nearest-neighbour (|*n* − *m*| = 2) charge-transfer configurations, respectively. In the presented framework, a CT state, |*n*, *m*〉, is defined as a state with a cation localized on molecule *n* and an anion localized on molecule *m*. Its energy depends on the electron–hole separation *via*114
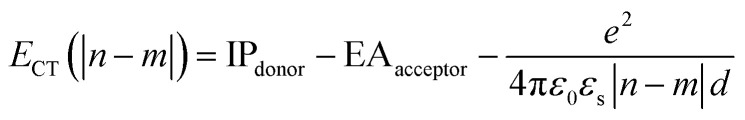
where IP_donor_ is the ionization potential of the donor and EA_acceptor_ is the electron affinity of the acceptor. The final term is a Coulomb stabilization term involving the elementary charge, *e*, vacuum permittivity, *ε*_0_, the static dielectric constant, *ε*_s_, of the surrounding medium and the nearest neighbour intermolecular distance, *d*. Thus *E*_CT_ (2), with |*n* − *m*| = 2, corresponds to a more weakly bound, more spatially separated cation–anion pair than *E*_CT_ (1), and is therefore higher in energy because the Coulomb stabilization term is smaller. Importantly, the existence of such a configuration does not require a direct orbital overlap between non-nearest-neighbour molecules. The overlap enters only through the hopping integrals *t*_e_ and *t*_h_, which connect different CT configurations and mix them with the Frenkel states. In the present model these transfer integrals are restricted to nearest-neighbour hops, so CT states with |*n* − *m*| = 2 are coupled to the Frenkel/CT manifold only *via* sequences of nearest-neighbour hops (for example |1, 2〉 → |1, 3〉 *via* an electron hop from site 2 to 3). As a result, the low-lying eigenstates are dominated by Frenkel and nearest-neighbour (|*n* − *m*| = 1) CT configurations, and the contribution from |*n* − *m*| ≥ 2 is small when *E*_CT_(2)–*E*_CT_(1) is large compared to *t*_e_ and *t*_h_. This observation motivates the commonly applied “nearest-neighbour CT” approximation in which only |*n* − *m*| = 1 states are retained and CT configurations with |*n* − *m*| ≥ 2 are removed, which will be demonstrated at the end of this example.

#### 6.1.2 Off-diagonal terms

As before, the off-diagonal elements fall into several classes, corresponding to different physical processes. The Frenkel ↔ Frenkel interactions originate from the excitonic Coulomb couplings *J*^Coul^_*mn*_ already introduced in Section 4.2.

115 〈*m*|*Ĥ*_M_|*n*〉 = *J*^Coul^_*mn*_ (*m* ≠ *n*)

Next, the Frenkel ↔ CT coupling part of the Merrifield Hamiltonian, *Ĥ*_F–CT_, describes processes in which a local neutral excitation on site *n* is converted to a CT state |*n*, *m*〉 configuration by hopping either the electron or the hole to a neighbouring site. These processes are quantified by the electron and hole hopping integrals *t*_e_ and *t*_h_.116
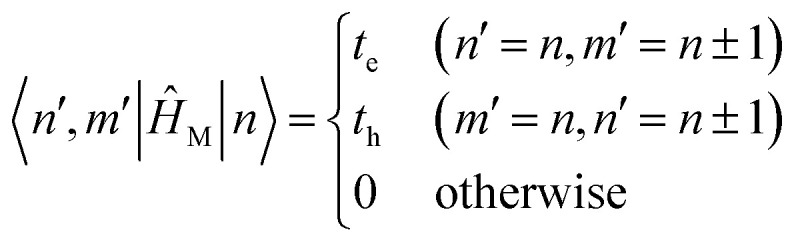


The Hermitian conjugate in *Ĥ*_F–CT_ ensures the corresponding reverse processes, *i.e.* 〈*n*|*Ĥ*_M_|*n*′,*m*′〉 = 〈*n*′, *m*′|*Ĥ*_M_|*n*〉*. Finally, the electron and hole transfers within the CT manifold gives the CT ↔ CT interactions according to:117
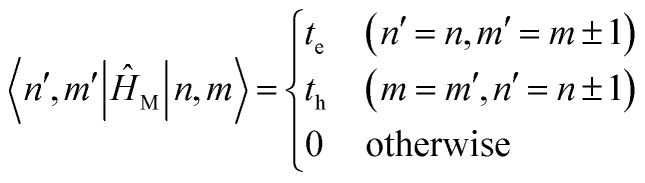
with the requirement that all site indices must remain within the system and that *m* ≠ *n* and *m*′ ≠ *n*′ since such cases belong to *Ĥ*_F–CT_ instead.

In this ordered basis the purely electronic Hamiltonian with CT contributions can be written in block form according to118
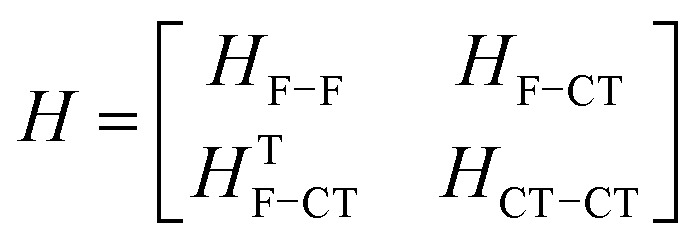
119
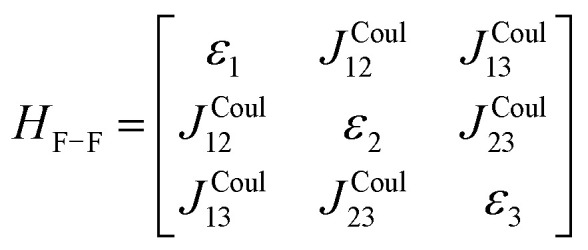
120
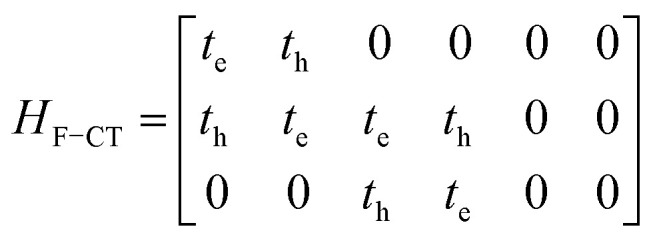
121
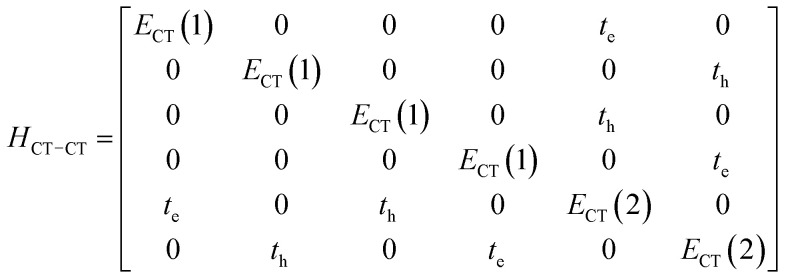


As expected, the Frenkel–Frenkel block, *H*_F–F_, in eqn (119) is identical to the *N* = 3 example derived for the purely electronic Frenkel Hamiltonian in Section 4.2. The new structure introduced by the Merrifield Hamiltonian resides entirely in the blocks that couple Frenkel and CT configurations and within the CT manifold itself. The off-diagonal elements are governed by the electron and hole transfer integrals *t*_e_ and *t*_h_ while the CT diagonals are set by the separation- dependent CT energies, *E*_CT_ (|*n* − *m*|).

#### 6.1.3 Nearest neighbour approximation

To facilitate the treatment of CT states, particularly for large (or even infinite) aggregates, the block matrix in eqn (118) can be simplified if one assumes that the static dielectric constant of the aggregate system is small, which is a good approximation for organic aggregates and crystals. This assumption can, however, break down in strongly screening environments, for example when the aggregates are embedded in or surrounded by highly polar media (high-dielectric solvents), electrolyte/salt solutions, or polar/ionic matrices, where dielectric screening can substantially reduce Coulomb interactions.^105^ Nevertheless, assuming a small dielectric constant, this will result in a large Coulomb binding energy that restricts the cation–anion pair from separating over more than one or two molecules. With this assumption, the energy of the CT state is assumed to be infinite for separations |*n* − *m*| ≥ 2:122
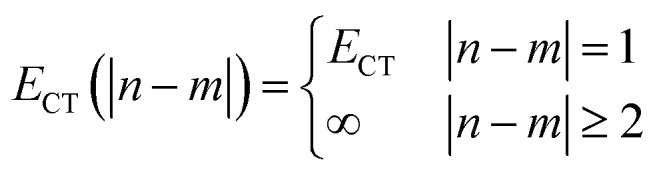


Consequently, in this nearest neighbour CT approximation, the corresponding basis states for |*n* − *m*| ≥ 2 are removed from the matrix representation. For the *N* = 3 example that means removing the basis states 8 and 9 in eqn (110). The new block Hamiltonian will then be a 7 × 7 matrix123
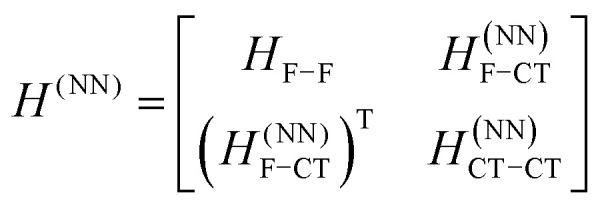


The Frenkel–Frenkel block is unchanged124
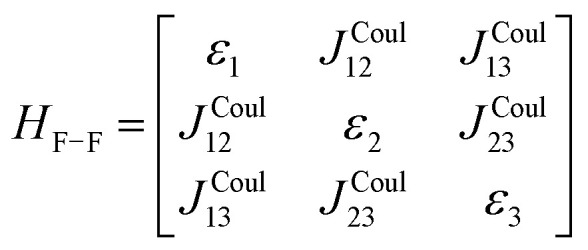


However, if one enforces nearest-neighbour Coulombic coupling in addition to CT, the *J*^Coul^_13_ = *J*^Coul^_31_ entries should be set to zero. The *H*^(NN)^_F–CT_ block is reduced to125
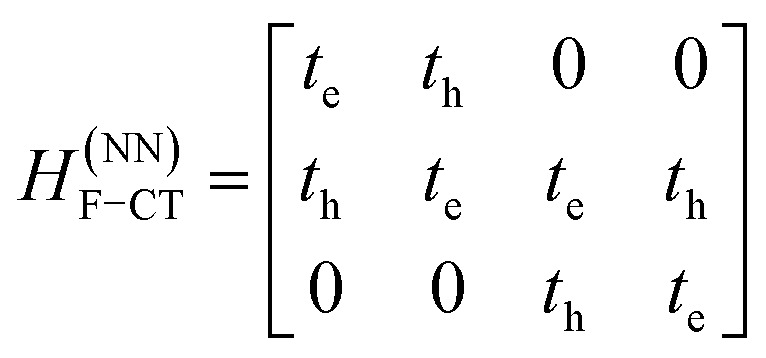


And *H*^(NN)^_CT–CT_ will become a purely diagonal matrix according to:126
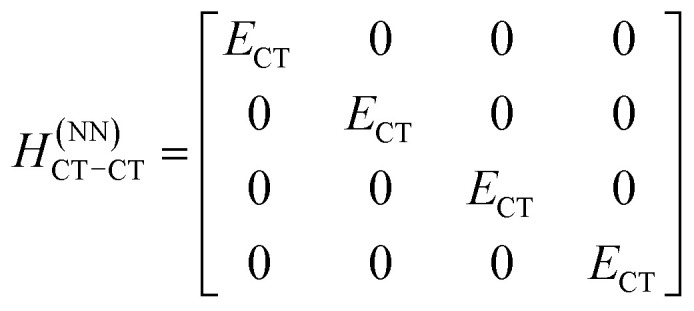


The reason is that all CT–CT off-diagonal elements connect |*n* − *m*| = 1 states (**4**–**7**) to |*n* − *m*| = 2 states (**8–9**). There are no CT–CT off-diagonal elements between |*n* − *m*| = 1 states alone. Any electron or hole hop from a |*n* − *m*| = 1 state occurs either to a Frenkel state or to a |*n* − *m*| = 2 CT state.

### 6.2 Charge transfer and vibronic coupling

In the preceding subsection, the electronically excited manifold was described by the purely electronic Merrifield Hamiltonian, *Ĥ*_M_, defined in eqn (105). This Hamiltonian includes neutral Frenkel excitons, CT configurations, and their mutual mixing *via* electron and hole transfer integrals *t*_e_ and *t*_h_. To account for the coupling between the electronic excitations, CT states, and an intramolecular vibrational mode, the Merrifield Hamiltonian can be extended in the same spirit as the Frenkel–Holstein model introduced in Section 5. As before, a single effective intramolecular mode of frequency *ħω*_vib_ on each molecule is considered. This mode is assumed harmonic in all relevant electronic manifolds (neutral ground, neutral excited, cationic, and anionic), with different equilibrium positions along the vibrational coordinate. The displacements are quantified by Huang–Rhys factors: *λ*^2^ for the neutral excited state and *λ*_+_^2^ and *λ*_−_^2^ for the cationic and anionic states, respectively. The values for *λ*_+_^2^ and *λ*_−_^2^ are system dependent and can be determined either experimentally or through theoretical calculations.^45,105–107^ For perylene-based π-stacks and related donor–acceptor systems, good fits to spectra have been obtained when the ionic Huang–Rhys factors are set to roughly half the neutral factor.^108,109^ This reflects the reduced structural relaxation of the macrocyclic stretching (or ring-breathing) mode upon ionization compared to neutral excitation. The resulting Merrifield–Holstein Hamiltonian, *Ĥ*_MH_, can be written as

127 *Ĥ*_MH_ = *Ĥ*_M_ + *Ĥ*_vib_ + *Ĥ*_F–vib_ + *Ĥ*_CT–vib_

where *Ĥ*_M_ was already defined in eqn (105), *Ĥ*_vib_ was defined in eqn (56) and *Ĥ*_F–vib_ was defined in eqn (57). The new term, *Ĥ*_CT–vib_, accounts for the coupling between the intramolecular vibrational mode and the CT configurations. In the notation introduced in the previous subsection, the state |*n*, *m*〉 represents a configuration with a hole localized on site *n* and an electron localized on site *m*. In such a CT state, the vibrational potential on the cationic site *n* is displaced by an amount characterized by *λ*_+_^2^, while the potential on the anionic site *m* is displaced by an amount characterized by *λ*_−_^2^. The vibronic coupling in the CT manifold can therefore be written as128
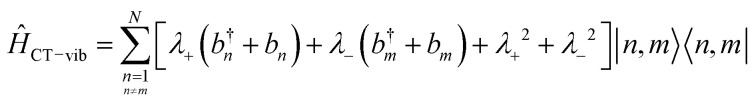
which is analogous to *Ĥ*_F–vib_ in eqn (57).

#### 6.2.1 Defining a basis set

To solve the Merrifield–Holstein Hamiltonian it is convenient to, as in the case of vibronic coupling in Section 5, use a multiparticle basis set, which is truncated at the two-particle level for the Frenkel excitons and at the three-particle level for the CT excitons. However, as in the case of pure vibronic coupling, there are regimes where higher order particle states should be included for quantitative accuracy. In particular, when the transfer integrals become large compared to the energy difference between the CT state and the local neutral excitation: |*t*_e_|, |*t*_h_| ≫ |*E*_CT_ − *ε*| or when states with |*n* − *m*| ≥ 2 are included in the basis, higher-order multiparticle configurations can acquire significant weight and the two-particle truncation may no longer be sufficient. In the present case the electronic excitation can be either a Frenkel exciton or a CT configuration, so both types appear in the one- and two-particle basis sets. The Frenkel exciton states are identical to what was presented in the Frenkel–Holstein section. Specifically, eqn (63) and (64) for the one- and two-particle states, respectively. They are, however, restated here for convenience:

One-particle states:

129 |*n*, *ṽ*〉

Two-particle states

130 |*n*, *ṽ*; *n*′, *v*′〉, (*n*′ ≠ *n*, *ν*′ ≥ 1)

where *ṽ* indicate vibrational quanta of molecule *n* in an electronically excited state, together with *v*′ vibrational quanta on a different molecule *n*′ in the ground electronic state. In a CT configuration, a cation resides on site *n*^+^ and an anion on site *m*^−^. A two-particle CT vibronic state therefore has the form

131 |*n*^+^, *v*_+_; *m*^−^, *v*_−_〉

Here, *v*_+_ are vibrational quanta in the cationic potential on molecule *n*^+^, and *v*_−_ are vibrational quanta in the anionic potential on site *m*^−^. Analogously to the Frenkel case, the three-particle CT state have a CT vibronic configuration accompanied by one additional vibrational quantum on a third, electronically neutral site. These states can be written according to

132 |*n*^+^, *v*_+_; *m*^−^, *v*_−_; *n*′, *v*′〉, (*n*′ ≠ *n*^+^ ≠ *m*^−^, *ν*′ ≥ 1)

**Fig. 11:**
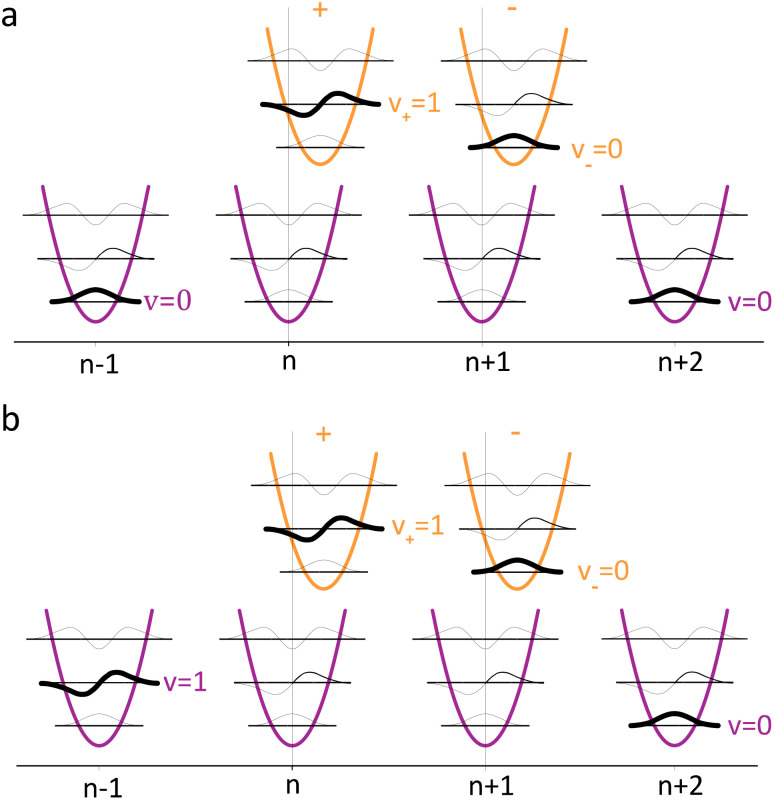
Illustration of the (a) two-particle CT state in which molecule *n* is a cation with vibrational quantum ***v***_+_ = 1 (indicated by the bolded line) and all other molecules (except the anion in position *n* + 1 with ***v***_−_ = 0) are in their electronic ground states and vibrational ground states; |***n***, 1; ***n*** + **1**, **0**〉 (b) three-particle CT state in which molecule *n* is once again a cation with vibrational quantum ***v***_+_ = 1 and the anion in position *n* + 1 has vibrational quantum ***v***_−_ = 0. In addition, one molecule in the system (*n* − 1) has a vibrational quantum ***v*** = 1 and all other molecules (except *n*, *n* + 1 and *n* − 1) are in their electronic ground states and vibrational ground states; |***n***, **1**; ***n*** + **1**, **0**; ***n*** − **1**, **1**〉.

The same truncation of the total number of vibrational quanta, *ν*_max_, used in the purely vibronic case in Section 5 (see eqn (61)) is applied here as well. A schematic illustration of the two- and three-particle CT state configurations is presented in Fig. 11.

### 6.3 Special case: energetically well separated CT and neutral excited states

As mentioned previously, when both Coulombic and CT interactions are present, the total excitonic coupling between two chromophores contains a long-range Coulomb term and an additional short-range, CT-mediated term. The former is the familiar Coulombic coupling, *J*^Coul^_*mn*_, that appears in the purely Frenkel and Frenkel–Holstein Hamiltonians introduced in Sections 4.2 and 5. The latter arises implicitly in the Merrifield Hamiltonian through the electron and hole transfer integrals *t*_e_ and *t*_h_, which couple a local neutral excitation to a nearest-neighbour CT configuration. In the general case these couplings must be treated explicitly by retaining CT states in the basis (the Merrifield–Holstein Hamiltonian). However, when the CT energy is energetically far from the neutral excited state energy, *ε*, the CT states can be integrated out, and their effect can be represented by an effective Hamiltonian acting solely in the Frenkel subspace. More precisely, when |*E*_CT_ (1) − *ε*| ≫ |*t*_e_|, |*t*_h_|, |*J*^Coul^_*mn*_|, *ħω*_vib_ it is possible to estimate an effective CT-mediated short range coupling term according to133
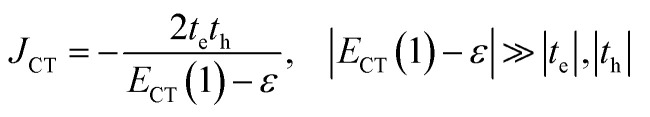
where the CT state serves as a virtual intermediate in a superexchange mechanism. In this limit it is convenient to define an effective coupling term, *J*^eff^_*mn*_, according to

134 *J*^eff^_*mn*_ = *J*^Coul^_*mn*_ + *J*_CT_*δ*_|*m*−*n*|,1_

where *δ*_|*m*−*n*|,1_ is the Kronecker delta defined as135
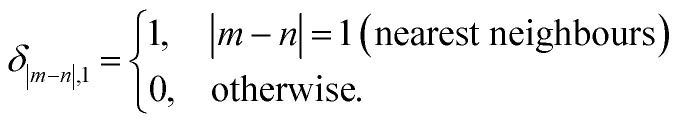


In the present context, this ensures that the CT-mediated coupling acts only between nearest neighbours, while the Coulombic couplings *J*^Coul^_*mn*_ are not subject to this restriction and may extend beyond nearest neighbours. Both this nearest-neighbour constraint on the CT term and any truncation of the Coulombic couplings can, however, be adjusted as appropriate for a given system. The resulting effective coupling can then be used in either the purely electronic Frenkel Hamiltonian (eqn (26)) or in the Frenkel–Holstein Hamiltonian (eqn (54)). In both cases, the excitation energy of the single molecule on site *n* acquires a small CT-induced energy correction, *Δ*_CT_, to the diagonal terms,136
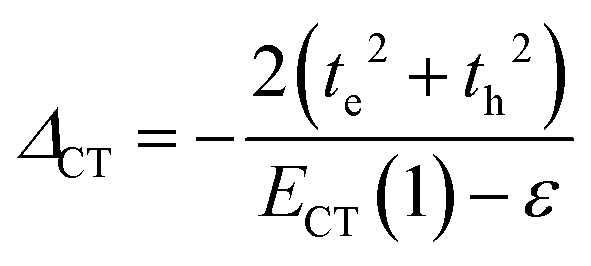
which arises from virtual electron and hole transfer to the CT manifold and back. Collecting these results, the corresponding CT-renormalized Frenkel and Frenkel–Holstein Hamiltonians – formally identical to their purely Frenkel counterparts but with the modified site energy and couplings defined above – can be written as follows:137
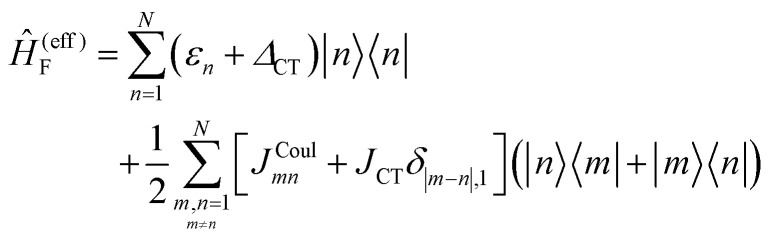

138 *Ĥ*^(eff)^_HF_ = *Ĥ*^(eff)^_F_ + *Ĥ*_vib_ + *Ĥ*_vib–el_

Here *ε*_*n*_ is the excited state energy of the single molecule on site *n* and *Δ*_CT_ is the corresponding CT-induced energy shift, and *Ĥ*_vib_ and *Ĥ*_vib–el_ are the vibrational and vibronic coupling terms defined in Section 5. No example is demonstrated for this special case since the procedure is identical to the examples presented in Sections 4.2 and 5 for the pure Frenkel and for the Frenkel–Holstein case, respectively.

### 6.4 How to practically calculate the one electron overlap integrals *t*_e_ and *t*_h_

In this review, *t*_e_ and *t*_h_ are defined as one-electron transfer integrals between localized frontier orbitals on neighbouring molecules (eqn (103) and (104)). In practice, there are two widely used methods to calculate *t*_e_ and *t*_h_, called the fragment orbital method and the energy-splitting method. Both methods start from the same input: a chosen dimer geometry (for example obtained from crystallographic data or from DFT geometry optimization). The difference is how the coupling is extracted.

In the case of the fragment orbital method one works in a basis of monomer-localized frontier orbitals and calculates (a) the orbital overlap integrals *S*_h_ (between HOMOs on molecules 1 and 2) and *S*_e_ (between LUMOs on molecules 1 and 2) (b) the site energies *E*_h/e,1_ (for the HOMO/LUMO on molecule 1) and *E*_h/e,2_ (for the HOMO/LUMO on molecule 2) and (c) the raw (non-orthogonal) couplings 
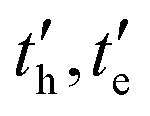
. Within the multiparticle basis the CT states are orthogonal to one another, which implies that the underlying HOMO and LUMO are also orthogonal. However, monomer-localized frontier orbitals derived from quantum chemical calculations are not, in general, orthogonal. The raw couplings 
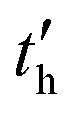
 and 
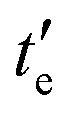
 must therefore be converted into an effective (orthogonalized) transfer integral *via* a Löwdin-type correction according to^110,111^139
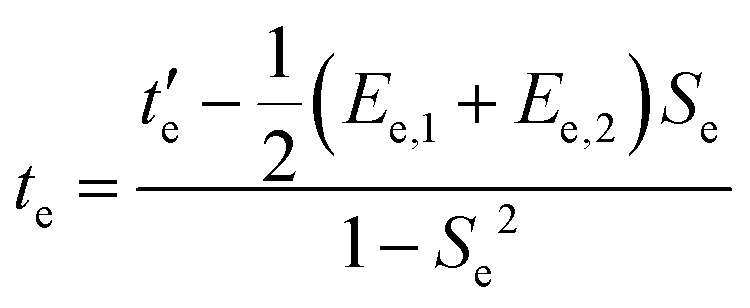
140
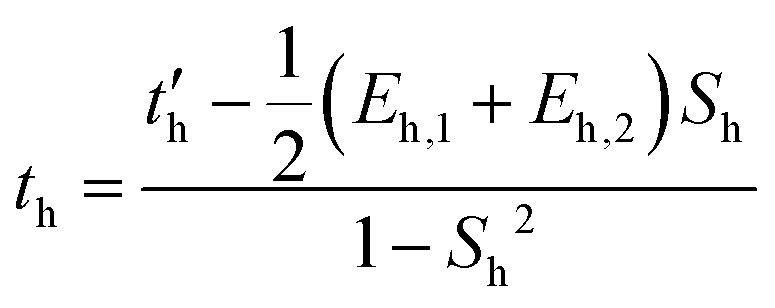


The energy-splitting method is a shortcut alternative in which the magnitudes of the transfer integrals are estimated from the frontier orbital energies of the dimer alone according to |*t*_h(e)_| ≈ Δ*E*/2. In this method, Δ*E* refers to the energy separation between the two dimer orbitals formed by mixing the same two monomer orbitals (bonding *vs.* antibonding combination). For holes this is typically the HOMO − (HOMO−1) gap, Δ*E*_h_ = *E*(HOMO) − *E*(HOMO−1). For electrons it is the (LUMO+1) − LUMO gap, Δ*E*_e_ = *E*(LUMO+1) − *E*(LUMO). This shortcut works only when the two molecules are effectively equivalent (related by a symmetry transformation) and when the relevant dimer frontier orbitals are clean symmetric/antisymmetric mixtures of the two monomer HOMOs (or LUMOs). If the dimer is asymmetric, or if other orbitals mix in (common in slipped π-stacks), the observed Δ*E* can be dominated by site-energy differences and polarization rather than by true inter-site coupling, leading to overestimation of *t*_e(h)_. For quantitative work it is therefore safer to compute *t*_e_ and *t*_h_ from the fragment orbital procedure.^110,112^ Moreover, in the energy-splitting method, the relative signs of *t*_e_ and *t*_h_, which have important consequences for whether the CT interaction induces J- or H-like behaviour in the aggregate, as shown in eqn (133), must also be assigned. This is typically done by visual inspection of the frontier molecular orbitals of the dimer (HOMO, HOMO−1, LUMO, and LUMO+1), as discussed in Section 5.8.3 of ref. 12. The fragment orbital procedure gives the signs of *t*_e_ and *t*_h_ as a part of the calculation, which is another attractive feature of this approach.

## 7. Properties of exciton coupled states

The previous chapters introduced the origins of intermolecular excitonic coupling, highlighting that long-range Coulombic interactions and short-range CT-mediated coupling can coexist in the same material. In many π-stacked crystals and aggregates these contributions can be comparable in magnitude and may interfere constructively or destructively. As a result, small changes in packing can strongly reshape absorption and emission linewidths, delocalization length, and radiative behaviour. These effects can in fact sometimes occur without producing an obvious “H-” or “J-like” shift in the steady state absorption and emission spectra. The sections below summarise key interference effects and outline practical spectroscopic signatures that can be used to diagnose different coupling regimes and aggregate types.

### 7.1 Interference between Coulombic and CT-mediated coupling

As shown in Section 6.3, when both Coulombic coupling (*J*^Coul^) and CT-mediated coupling (*J*_CT_) contribute to the interaction between molecules, the net coupling can, when |*E*_CT_ − *ε*| ≫ |*t*_e_|, |*t*_h_|, |*J*^Coul^_*mn*_|, *ħω*_vib_, be treated as approximately additive according to eqn (133). If *J*^Coul^ and *J*_CT_ have the same sign, constructive interference (HH or JJ) increases the coupling strength and exaggerates the corresponding H- or J-like vibronic signatures and associated properties. In contrast, opposite signs (HJ or JH) produce destructive interference that reduces the total coupling strength and can suppress the spectral signatures normally used to identify aggregate type, despite large underlying coupling strengths. This interference motivates an expanded aggregate classification, where the first letter denotes the sign of the Coulombic (long-range) contribution and the second letter denotes the sign of the CT-mediated (short-range) contribution, with upper-/lower-case sometimes used to indicate relative magnitude (hJ, jH, *etc.*). At the extreme of destructive interference, a “null point” can be reached where *J*^Coul^ ≈ −*J*_CT_. In this limit, absorption and photoluminescence can appear strikingly monomer-like even though the chromophores remain closely packed, making “null aggregates” an important cautionary case for structure assignment based on optical spectra alone. The interference between Coulombic and CT-mediated coupling is easy to appreciate when |*E*_CT_ − *ε*| ≫ |*t*_e_|, |*t*_h_|, |*J*^Coul^_*mn*_|, *ħω*_vib_ holds true. However, the underlying competition between Coulombic and CT pathways remains relevant even when the local neutral excited state and CT state are closer in energy.

In Coulomb–CT aggregates, the two contributions compete within the same nearest-neighbour pair (sometimes termed integrated interference). HJ behaviour can, however, also arise in a purely Coulombic setting when H-type and J-type Coulombic couplings occur along different directions in the same aggregate (*e.g.*, head-to-tail coupling along one axis and side-by-side coupling along another). This situation is sometimes referred to as a segregated HJ aggregate. A monomer-like (“null”) absorption spectrum can occur in segregated HJ aggregates as well.^80^ However, in this case the cancellation applies primarily to the optically active excited state and thus the optical response rather than eliminating intermolecular coupling throughout the aggregate altogether. A null-like spectrum therefore does not necessarily mean that interactions are weak overall. This distinction is important for transport. In an integrated Coulomb–CT null aggregate, cancellation occurs within the same nearest-neighbour interaction so the effective coupling can become very small, which is expected to hinder energy transport.^113^ In a segregated Coulombic null aggregate, substantial couplings can still operate along particular directions even though the excited state optical response cancels, so exciton transport can remain comparatively efficient despite the null-like optical spectrum. Segregated HJ aggregates can also display distinct radiative behaviour. The lowest-energy excited state can be weakly emissive, while a brighter state may lie slightly higher in energy. Increasing temperature can thermally populate the higher-energy bright state, thereby increasing the overall radiative rate (and apparent emission intensity), with the enhanced emission originating primarily from the bright state rather than the lowest-energy weakly emissive state. This has for instance been observed in perylene diimide π-stacks assigned as Hj *vs.* hJ. In this case, Hj behaviour is associated with a weak 0-0 emission feature that grows with increasing temperature, whereas hJ behaviour shows a dominant 0-0 emission peak and a 0-0/0-1 ratio that is strongly enhanced (and nearly temperature-independent).^113,114^

### 7.2 Packing sensitivity: why small slips often matter more for CT than for Coulomb coupling

In general, CT-mediated coupling is far more sensitive to geometry than Coulombic coupling. This is because *J*_CT_ depends on frontier-orbital overlap (*via t*_e_ and *t*_h_), which is strongly shaped by the nodal structure of the HOMO/LUMO. For typical organic molecules these nodal patterns vary on bond-length scales, so sub-Å to Å slips can markedly change the magnitude and even the sign of *J*_CT_. By contrast, *J*^Coul^ is governed by longer-range electrostatics and generally varies more smoothly with displacement. In simple dipole/extended-dipole pictures, reversing the interaction from “side-by-side” (repulsive-type) to “head-to-tail” (attractive-type) typically requires lateral slips on the order of a molecular dimension. Thus, a useful rule of thumb is that *J*_CT_ can vary on bond-length scales, whereas *J*^Coul^ varies on molecular-length scales.

For perylene systems, the short-range term can change sign over displacements at the order of ∼1–2 Å, while much larger displacements are required to invert the Coulombic sign. In for instance conventional π-stacks, this often means that the long-range contribution is robustly H-like, while the short-range CT term can be tuned to be either H- or J-type through relatively small packing changes. This sensitivity has concrete consequences as shown for example in ref. 105, 111, 115 and 116 where slips on the Å scale were shown to strongly alter the balance between Coulombic and CT pathways and thereby switch between destructive and constructive interference, with large effects on spectra, excited state delocalization and transport. It should also be mentioned that null behaviour in aggregates can arise for a different reason than cancellation of interactions. For example, if both Coulombic and CT interactions are intrinsically small due to packing geometry. An example of this is cross-stacked (near-orthogonal) pentacene derivatives where the exciton coupling is reported to be very small, giving monomer-like optical behaviour without requiring opposing Coulombic and CT contributions.^117^

It should also be noted that alternative (and complementary) notation for coupling interferences has been proposed. Caram and co-workers proposed an H/I/J classification that focuses on the energetic position of the optically allowed state relative to lower-lying dark states, rather than on a single net “H or J” label. In this picture, an I-aggregate can show a red-shifted bright state (J-like absorption) while still having lower-energy dark states, which can rationalize cases with red-shifted absorption but suppressed (or thermally activated) emission.^118^

### 7.3 A summary of spectroscopic metrics for coupling, coherence and radiative behaviour

It has been demonstrated throughout this review, in particular in the vibronic coupling Section 5.5, that steady-state absorption and emission spectra can provide practical metrics for excitonic coupling strength, coherence length, and radiative behaviour. Here comes a summary of some of the most important points. In the simplest homodimer picture with a single dominant Coulombic interaction, *J*^Coul^, the formed hybrid states are split by Δ*E* ≈ 2|*J*^Coul^|, and the optically allowed transition is shifted by roughly ±|*J*^Coul^| relative to the monomer (the sign depends on whether it is a J- or H-aggregate). For an ideal one-dimensional aggregate with only nearest-neighbour coupling, *J*^Coul^_NN_, the highest and lowest energy hybrid states are instead split by ∼4|*J*^Coul^_NN_|, with additional hybrid states lying between these two extremes. These relations remain useful as rough estimates, but they can fail or become ambiguous once longer-range couplings, multiple molecules per unit cell, vibronic coupling, disorder, or CT mixing become important. A more robust route, especially at intermediate coupling strengths, is to use vibronic intensity ratios. As discussed in the vibronic coupling sections, the relative intensity of the first two vibronic peaks in the absorption spectra reports primarily on the effective excitonic coupling as demonstrated with eqn (101). In essence, the 0-0/0-1 ratio increases with increasing J-like character and decreases with increasing H-like character compared to the monomer 0-0/0-1 ratio.

The corresponding ratio of the 0-0 and 0-1 emission transitions is more directly linked to coherence in the emitting state, because the 0-0 strength depends on the phase-coherent sum of transition dipoles, whereas the vibronic sideband is not coherently enhanced. For disordered J-aggregates under common experimental conditions, this connection can be made quantitative *via* the “emission ratio rule” in eqn (100). This equation enables the coherence number, *N*_coh_, to be estimated directly from steady-state emission spectra.

These vibronic ratios also provide a natural bridge to the interference discussion in the previous section. In an aggregate, when the ratio of the first and second absorption transition is close to the monomer value, the net effective coupling is likely small. This is consistent with destructive interference and a reduced energy transport efficiency, whereas large deviations from the monomer ratio indicate strong coupling and, often, more efficient exciton motion.

Linewidths and line-shapes provide complementary information but require additional caution. A recurring observation in molecular aggregates is linewidth narrowing, where delocalisation averages over site-energy disorder and reduces the apparent width of optical transitions. In many practical treatments, the absorption linewidth narrows roughly with increasing delocalisation length (often scaling approximately as 
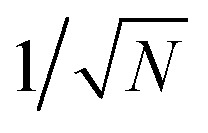
), and this behaviour has been widely used as an additional proxy for coherence.^119^ However, linewidths are shaped by multiple contributions (static disorder, homogeneous dephasing, and vibronic structure). Furthermore, in the case of emission, apparent narrowing can occur even without strong excitonic coupling simply because emission preferentially originates from lower-energy sites after relaxation. Consequently, linewidth narrowing should be used with caution when estimating coherence lengths and numbers.

Excited state delocalisation can also reduce the effective nuclear reorganisation accompanying optical excitation. Intuitively, when the excited state is shared over *N*_coh_ chromophores, each molecule experiences only a fraction of the electronic redistribution and therefore undergoes a smaller structural relaxation. In the simplest picture where the dominant reorganising motions are similar on each molecule and the excitation is coherently distributed, the total reorganisation required to form the delocalised excited state is reduced approximately in proportion to 1/*N*_coh_, corresponding to a smaller vibrational contribution to the effective reorganisation energy.^119^ In practice, the magnitude of this reduction depends on how local the relevant nuclear motions and environmental fluctuations are, but the trend provides a useful physical link between delocalisation, vibronic structure, and linewidth narrowing. A lower reorganization energy is spectrally observed as a reduced Stokes shift. Furthermore, a lower reorganisation energy can also improve radiative efficiency by weakening the usual energy-gap-law losses, which has been proposed as one reason some delocalised excitonic systems remain bright even in the NIR.^7^

Radiative behaviour adds another diagnostic dimension. In ideal J-aggregation, the lowest-energy hybrid state has an enhanced transition dipole, sometimes referred to as “superradiance”.^120^ For an aggregate the enhancement can scale approximately with the number of coherently coupled chromophores. In real materials, disorder and localisation reduce this enhancement and can suppress superdiant signatures. Importantly, the Coulomb and CT interference mechanisms highlighted above can modulate radiative rates indirectly by changing the effective coupling and the coherence length, and directly by reshaping which excited states carry oscillator strength.

### 7.4 Screening of Coulombic coupling

Dielectric screening enters the Coulombic part of the exciton coupling by replacing the vacuum permittivity with an effective permittivity of the environment. In its simplest form (*e.g.*, point-charge or transition-density Coulomb integrals), this is implemented by dividing the interaction by the optical dielectric constant, *ε*_opt_ (often approximated as the square of the refractive index at high frequencies), of the medium141
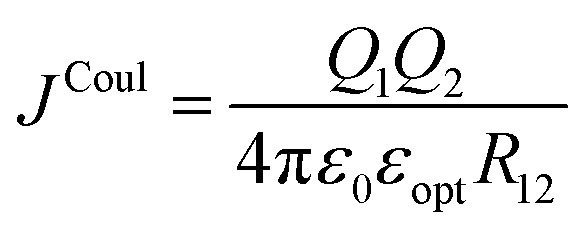


This treatment implicitly assumes the interacting transition densities are embedded in a homogeneous dielectric environment and the chromophores are taken as point-like sources (or fixed charge distributions) sitting in a continuum. Furthermore, the optical, rather than the static, dielectric constant is the appropriate screening parameter for transition-transition Coulomb coupling because the interaction involves oscillating electronic transition densities at high frequencies. Since nuclear reorientation is effectively frozen on the timescale of an electronic transition, only the electronic polarizability contributes to screening of the transition density-transition density interaction. However, the static dielectric constant can still matter indirectly, because it can shift excitation energies and CT-state energies (and thus the CT-neutral excitation energy difference), thereby affecting the degree of CT-neutral excitation mixing.

In solid-state aggregates, it is common to treat screening with an effective optical dielectric constant of the solid (sometimes as an adjustable parameter).^115^ This approach implicitly assumes an isotropic, spatially uniform environment. In real crystals the dielectric response can be anisotropic and even distance dependent at short range. In such cases, a spatially averaged optical dielectric constant may be estimated as shown in ref. 121 yielding an average *ε*_opt_ of approximately 3 for perylene crystals. Typical values for organic molecular solids are often in the range 2–4.

The “divide-by-*ε*_opt_” procedure treats the chromophores as if they experience the macroscopic field of a continuum. A common refinement is to include local-field corrections, which account for the fact that a molecule occupies a finite region that disrupts the dielectric continuum (“a hole” or “cavity” in the medium). Different expressions for these local-field corrections exist depending on the shape and properties of the region of space that the molecule occupies.^74,122^ For instance, the empty cavity model assumes that the molecule occupies a spherical vacuum region which gives the local field correction term: *η*_c_ = 3*ε*_opt_/(2*ε*_opt_ + 1). Another model is the virtual hole model which also assumes that a spherical part of the medium is missing. However, in this model, it is assumed that the molecule in the virtual hole has the same dielectric constant as the surroundings but does not contribute to the local field at its own position. In this case the correction is called the Lorentz field factor according to *η*_L_ = (*ε*_opt_ + 2)/3. These models modify the field produced by one transition dipole at the position of the other (and *vice versa*), so the interaction energy carries two factors of the field correction. Consequently, the overall correction to the interaction energy is142
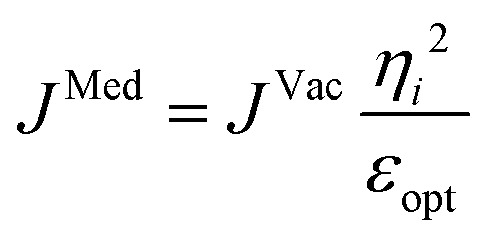
where *J*^Med^ is the effective coupling strength in the medium and *J*^Vac^ is the coupling strength in vacuum. More elaborate variants also exist to account for non-spherical cavities and more realistic molecular shapes.^123,124^

The local-field models above implicitly assume two separate cavities (each chromophore in its own cavity embedded in the dielectric medium). However, when chromophores become so close that there is little or no intervening medium, it can be more appropriate to treat them as if they are residing in a common (shared) cavity. In this regime, the dielectric does not act as a simple uniform screening factor. Instead, polarization at the shared cavity boundary results in an additional term whose sign depends on dipole orientation.^125,126^ As a result, increasing *ε*_opt_ does not necessarily imply weaker coupling. Depending on geometry, the effective coupling can become less screened than predicted by a simple 1/*ε*_opt_ scaling and may even be enhanced relative to the vacuum value for in-line (head-to-tail, J-like) arrangements, while being further suppressed for side-by-side (H-like) arrangements.^126^

As a final note, local-field corrections can be useful, but they should be applied with care. They rely on idealized cavity and continuum assumptions (shape, boundary definition, homogeneity) that are often not uniquely defined for real aggregates and are therefore difficult to validate quantitatively by experiment.

## 8. Applications and future directions

Having established how strong exciton coupling can be modelled and diagnosed throughout this review, it is useful to briefly outline where these concepts matter for applications, and which open questions appear most pressing.

Exciton-coupling models can be said to primarily serve two complementary roles. On the one hand they rationalise the photophysics of densely packed materials such as crystals, neat films, and supramolecular assemblies. On the other they offer design rules in which packing (or molecular architecture) is treated as an adjustable parameter rather than a fixed outcome. In both contexts, however, the central challenge is that the effective coupling is often more complex than the simplest classifications suggest. A recurring theme in this review is that the underlying coupling landscape can be more nuanced than a simple “H *versus* J” label. H-like and J-like interactions may coexist along different directions, and Coulombic and CT-mediated contributions can act together. Consequently, similar spectral shifts can correspond to very different optical and transport behaviour, particularly when interference redistributes oscillator strength or suppresses effective coupling along a specific pathway.

From an application standpoint, coupling motifs influence three connected outcomes. The first is how efficiently excitations move. The second is how strongly, how narrowly, and at which energy a material absorbs and emits light. The third is how relaxation is partitioned between radiative and non-radiative channels.

The final aspect is particularly relevant for low-energy (near-IR) emitters, where non-radiative decay often dominates. Excited state delocalisation can reduce the structural relaxation required after excitation because the electronic redistribution is shared over several chromophores. Lower effective reorganisation energy can then weaken energy-gap-law losses and help sustain emission efficiency at longer wavelengths.

For exciton transport, relevant applications are for instance light harvesting and charge generation (solar cells, photocatalysis). After photoexcitation, an exciton must typically reach a dissociating interface before recombination. In many planar heterojunction architectures, this length scale can exceed typical exciton migration lengths. Coupling motifs that favour efficient energy transport while suppressing radiative loss are therefore often desirable. In the expanded aggregate picture, this then points toward H-dominated coupling cases (*e.g.*, ideally HH, but also Hj, jH), where strong coupling can coexist with weak emission. Other requirements also matter for these applications, such as broadband absorption, but these features may also be tuneable through molecular and packing design.

For emission applications, such as OLEDs and related emitters, narrow and intense emission is often a central target. Here, J-dominated scenarios are natural candidates because the optically bright state can carry an enhanced oscillator strength and reduced vibronic sidebands (superradiant-like behaviour under favourable coherence conditions). In the expanded classification, this corresponds to JJ or Jh-type behaviour. However, a practical complication is that many OLED architectures rely on doped emissive layers, where enforcing a specific crystalline packing motif at low concentration can be challenging. Furthermore, although high mobility and high photoluminescence are often treated as competing goals, there is no fundamental requirement that they be mutually exclusive. Instead, they are sometimes anticorrelated because the same morphologies that enable transport can also activate non-radiative channels (traps, excimers). Additionally, a key practical message from the CT–Coulomb interference discussion is that not all coupling channels are equally sensitive to packing. CT-mediated contributions depend on orbital overlap and can change sign with sub-Å to Å-scale slips, while Coulombic contributions typically vary more smoothly with geometry. This extreme sensitivity implies that modest structural perturbations (side-chain substitution, pressure, strain, shear) can switch between constructive and destructive interference and thereby reshape both photophysics and transport.

Applying exciton-coupling design rules to devices requires acknowledging that thin films are rarely homogeneous. Inhomogeneous crystallinity and mixed packing motifs can produce spatially varying coupling patterns and therefore spatially varying photophysical and transport behaviour. Consequently, strategies that reduce morphological variance such as intramolecular exciton coupling could be a complementary route to packing engineering. Here, two or more chromophores are held at a defined geometry using a rigid (or semi-rigid) linker. The attraction of this approach is twofold. First, it can deliver strong and well-defined coupling without relying on crystallinity or long-range order. Second, the resulting coupled emitter/absorber can often be processed like a conventional dye, making it compatible with architectures that require dilute or host–guest conditions. The main cost is synthetic complexity and the need to manage additional constraints such as conformational freedom, solubility and aggregation propensity. We are currently focused on incorporating intramolecular strong exciton coupling into dyes commonly used for organic light emitting diodes. Although synthetically challenging to make such dyes, we hope to show that in principle all figure of merits (singlet–triplet energetics, emission rates, linewidths *etc.*) can improve by the coupling. This idea highlights a transition from where strong exciton coupling is used to understand the photophysics in crystalline materials, to a future where it is used as a design tool when constructing new organic semiconductors, that are not necessarily restricted by the same limitations as traditional dyes, opening pathways for more efficient photophysical processes.

## Author contributions

The manuscript was written with contributions from all authors.

## Conflicts of interest

There are no conflicts to declare.

## Supplementary Material

CS-055-D6CS00157B-s001

## Data Availability

Supplementary information: nomenclature and derivations. See DOI: https://doi.org/10.1039/d6cs00157b. Data for this article, including scripts and calculation files are available at Github† or the Swedish National Data Service.‡
