# Supplementary material for: Strong exciton coupling: a practical toolbox for computing interaction energies, wavefunctions, and optical spectra
Source: Chem Soc Rev. 2026 May 19;55(12):6462–99. doi: 10.1039/d6cs00157b (PMC13185484; doi:10.1039/d6cs00157b)
Supplement: CS-055-D6CS00157B-s001 [file CS-055-D6CS00157B-s001.pdf]

Supplementary Information (SI) for Chemical Society Reviews.
This journal is © The Royal Society of Chemistry 2026

Supplementary Information for

Strong Exciton Coupling: A Practical Toolbox for Computing Interaction Energies, Wavefunctions, and Optical Spectra

Rasmus Ringström,[a] S. Rasoul Hashemi,[a] Yuanxin Liang,[a] Nicholas J. Hestand,[b] and Karl Börjesson[a]†

[a] Department of Chemistry and Molecular Biology, University of Gothenburg, Box 462, 405 30 Gothenburg, Sweden

[b] Department of Natural and Applied Science, Evangel University, 1111 N. Glenstone Ave. Springfield, MO 65802, United States

Email: karl.borjesson@gu.se

Contents

1 Nomenclature 2

2 Point-dipole approximation derivation 7

3 Extended dipole approximation derivation 9

4 Derivation of Hamiltonian matrix entries for a dimer 10

5 References 11

1 Nomenclature

α	Hybrid state index
$\rho(\mathbf{r})$	Charge density
$\rho^{tr}(\mathbf{r})$	Transition charge density
ε_0	Vacuum permittivity
ϵ_s	Static dielectric constant
ϵ_{opt}	Optical dielectric constant
ϵ	Neutral excited state energy
ϵ_n	Neutral excited state energy of molecule *n*
σ	Row index
τ	Column index
λ^2	Huang-Rhys factor
Δ_{CT}	Charge transfer induced energy correction
η_c	Local field correction term
η_L	Lorentz field factor
λ_+^2	Huang-Rhys factor of cationic state
λ_-^2	Huang-Rhys factor of anionic state
λ^2	Huang-Rhys factor of neutral excited state
$\tilde{v}$	Vibrational quanta in the excited state potential
v	Vibrational quanta in the ground state potential
ν_{max}	Maximum total vibrational excitations considered
μ_i	Transition dipole moment vector
$\hat{\boldsymbol{\mu}}_n$	Transition dipole moment unit vector
$\hat{\boldsymbol{\mu}}$	Transition dipole moment operator
$\lvert\boldsymbol{\mu}_n\rvert$	Transition dipole moment magnitude
ω_{vib}	Angular frequency of a vibrational mode

ω_{em}	Angular frequency of the lowest energy hybrid state
κ	Dipole orientation factor
$\varphi_n^{e/g/+/-}$	Electronic wavefunctions of molecule *n* in the excited (e), ground (g), cationic (+) or anionic (-) state
ψ_α	Wavefunction of hybrid state alpha
$\lvert\psi_{em}\rangle$	Wavefunction of the lowest energy hybrid state
Ψ	Wavefunction
χ_0^e	Vibrational wavefunction in the excited state
χ_0^g	Vibrational wavefunction in the ground state
$A(\omega)$	Absorption as a function of the angular frequency
AO	Atomic orbital
b_n	Vibrational annihilation operator
$b_n^\dagger$	Vibrational creation operator
c_n^α	Hybrid state coefficient
C	Eigenvector matrix
CT	Charge transfer
**dₙ**	Displacement vector for opposite point charges in extended dipole approximation
e	Excited state
E	Eigenvalue matrix
E_{CT}	Charge transfer state energy
$E_{CT}(1)$ molecules	Charge transfer state energy with cation and anion on neighbouring
$Em(\omega)$	Emission as a function of the angular frequency
ESP	Electrostatic-potential
FFT	Fast Fourier transform
f_α	Oscillator strength of hybrid state α

$F_{\tilde{v},v}$	Overlap integral of vibronic ground state and vibronic excited state
g	Ground state
$\vert g\rangle = \varphi_1^g \varphi_2^g \dots \varphi_N^g$	electronic aggregate ground state in which all molecules are in their electronic ground state
$\vert G\rangle = \vert g; 0_1, 0_2, \dots, 0_N\rangle$	electronic aggregate ground state in which all molecules are in their electronic and vibrational ground state
$\mathrm{G}(\nu_t; \lambda^2)$	Vibrational function
$\hat{h}$	Single electron Hamiltonian operator
H	Hamiltonian matrix
H_{mn}	Hamiltonian matrix element
$H_{\boldsymbol{\sigma\tau}}$	Hamiltonian matrix element
$\hat{H}$	Hamiltonian operator
$\hat{H}_F$	Frenkel Hamiltonian operator
$\hat{H}_{FH}$	Frenkel-Holstein Hamiltonian operator
$\hat{H}_{F-CT}$	Frenkel-CT mixing Hamiltonian operator
$\hat{H}_{vib}$	Vibrational mode Hamiltonian operator
$\hat{H}_{F-vib}$	Exciton-vibration coupling Hamiltonian operator
$\hat{H}_{CT}$	CT Hamiltonian operator
$\hat{H}_M$	Merrifield Hamiltonian operator
$\hat{H}_{MH}$	Merrifield-Holstein Hamiltonian operator
I^{0-v_t}	Emission line strengths to vibrational level v_t
J^{Coul}	Coulombic coupling strength
J_{TDC}^{Coul}	Coulombic coupling strength from the TDC method
$\mathrm{J}_{\mathrm{TrC}}^{Coul}$	Coulombic coupling strength from the TrC method
$\mathrm{J}_{\mathrm{dipole-dipole}}^{Coul}$	Coulombic coupling strength from the point dipole approximation method

J_{ext}^{Coul}	Coulombic coupling strength from the extended dipole approximation method
J_{CT}	CT-mediated effective coupling term
l_n	Length of displacement vector for point charges in extended dipole approximation
n	Molecule index
$\lvert n\rangle$	Basis set with molecule n electronically excited and all others in their ground states
$\lvert n,\tilde{v}\rangle$	one particle state basis representing an electronic excitation localized on molecule *n* together with $\tilde{v} = 0{,}1{,}2$ ... vibrational quanta
$\lvert n,\tilde{v};n',v'\rangle$	two particle state basis representing an electronic excitation with $\tilde{v}$ vibrational quanta on molecule *n*, together with v' vibrational quanta on a different molecule n' in the ground electronic state
N	Total number of molecules in the system
NN	Nearest neighbour
NNN	Next nearest neighbour
N_{coh}	Coherence number
m	Molecule index
M_{eff}	Effective mass
$P_{\mathrm{k}\xi}^{tr}$	Atomic orbital basis
Q	Point charge
$\mathbf{q}_i$	Transition charge
r	Scalar distance between point charges
**r**	Position in 3D space
**R**	Displacement vector
$\lvert\boldsymbol{R}\rvert = \mathrm{R}_{12}$	Scalar separation
S = λ^2	Huang-Rhys factor of neutral excited state
S_h	HOMO-HOMO orbital overlap integral

S_e	LUMO-LUMO orbital overlap integral
$S_{\mathrm{k}\xi}$	Atomic orbital overlap matrix
S_0	Singlet ground state
S_1	First singlet excited state
T_1	First triplet excited state
t_e	One-electron hopping integral
t_h	One-hole hopping integral
TDC	Transition density cube
TDDFT	Time-dependent density functional theory
TDFI	Transition density fragment interaction
TDHF/CIS	Time-dependent Hartree–Fock/Configuration Interaction Singles
TrC	Transition charge
TrCAMM	Transition cumulative atomic multipole moments
TrESP-CDQ	Transition charge, dipole, and quadrupole from electrostatic potential
ΔV_i	Voxel volume
W_{LS}	Homogeneous line shape function
$\hat{\boldsymbol{x}}$	Unit vector along the x-axis
$\hat{\boldsymbol{y}}$	Unit vector along the y-axis

2 Point-dipole approximation derivation

Start with the full expression for the Coulomb interaction of two transition densities

$$J = \frac{1}{4\pi\epsilon_0}\iint \frac{\rho_1^{tr}(\boldsymbol{r_1})\rho_2^{tr}(\boldsymbol{r_2})}{|\boldsymbol{r_1}-\boldsymbol{r_2}|} d\boldsymbol{r_1}d\boldsymbol{r_2} \qquad (S1)$$

Prepare the expression for Taylor expansion and introduce the centre-centre separation, $\boldsymbol{R}$. Let $\boldsymbol{r_1} = \boldsymbol{center\ of\ 1} + \boldsymbol{\Delta r_1}$ $\quad \boldsymbol{r_2} = \boldsymbol{center\ of\ 2} + \boldsymbol{\Delta r_2}$. Where $\boldsymbol{\Delta r_1}$ and $\boldsymbol{\Delta r_2}$ are small displacements (relative to the centre of each distribution) of charge compared to $\boldsymbol{R}$. The denominator can now be rewritten: $|\boldsymbol{r_1}-\boldsymbol{r_2}| = |\boldsymbol{R} + (\boldsymbol{\Delta r_1} - \boldsymbol{\Delta r_2})| = |\boldsymbol{R}+\boldsymbol{s}|$ where $(\boldsymbol{\Delta r_1} - \boldsymbol{\Delta r_2}) \equiv \boldsymbol{s}$. The full expression now reads

$$J = \frac{1}{4\pi\epsilon_0}\iint \frac{\rho_1^{tr}(\boldsymbol{r_1})\rho_2^{tr}(\boldsymbol{r_2})}{|\boldsymbol{R}+\boldsymbol{s}|} d\boldsymbol{r_1}d\boldsymbol{r_2} \qquad (S2)$$

We want to evaluate

$$f(s) = \frac{1}{|\boldsymbol{R}+\boldsymbol{s}|} \qquad (S3)$$

When $\boldsymbol{R} >> \boldsymbol{s}$ around $\boldsymbol{s} = (s_x,s_y,s_z)$. Since $\boldsymbol{s}$ is a vector variable the Taylor expansion takes the form

$$\boldsymbol{f(s)} \approx \boldsymbol{f(0)} + \sum_{i=x,y,z} \left.\frac{\partial \boldsymbol{f}}{\partial \boldsymbol{s_i}}\right|_{\boldsymbol{s=0}} \boldsymbol{s_i} + \frac{\boldsymbol{1}}{\boldsymbol{2}} \sum_{i,j=x,y,z} \left.\frac{\partial^2 \boldsymbol{f}}{\partial \boldsymbol{s_i}\partial \boldsymbol{s_j}}\right|_{\boldsymbol{s=0}} \boldsymbol{s_i s_j} + \dots \qquad (\boldsymbol{S}4)$$

Resulting in

$$\frac{1}{|\boldsymbol{R}+\boldsymbol{s}|} \approx \frac{1}{R} - \frac{\boldsymbol{R}\cdot\boldsymbol{s}}{R^3} + \frac{3(\boldsymbol{R}\cdot\boldsymbol{s})^2 - R^2 s^2}{2R^5} + \cdots \qquad (S5)$$

Where the first term is the zeroth order, the next term is the first order and so forth. Inserted into the integral expression leads to

$$J = \frac{1}{4\pi\epsilon_0}\iint \rho_1^{tr}(\boldsymbol{\Delta r_1})\rho_2^{tr}(\boldsymbol{\Delta r_2}) \left[\frac{1}{R} - \frac{\boldsymbol{R}\cdot\boldsymbol{s}}{R^3} + \frac{3(\boldsymbol{R}\cdot\boldsymbol{s})^2 - R^2 s^2}{2R^5}\right] d\boldsymbol{\Delta r_1} d\boldsymbol{\Delta r_2} \qquad (S6)$$

Where each term can be evaluated separately. For the zeroth order (monopole-monopole) term

$$J_0 = \frac{1}{4\pi\epsilon_0}\frac{1}{R}\iint \rho_1^{tr}(\boldsymbol{\Delta r_1})\rho_2^{tr}(\boldsymbol{\Delta r_2}) d\boldsymbol{\Delta r_1} d\boldsymbol{\Delta r_2} \qquad (S7)$$

The monopole terms

$$Q_i = \int \rho_i^{tr}(\boldsymbol{\Delta r_i})\, d\boldsymbol{\Delta r_i} \qquad (S8)$$

Are zero since the total charge is unchanged in the transition. Thus, J_0=0. Next is the first order (monopole-dipole) term

$$J_1 = \frac{1}{4\pi\epsilon_0}\frac{1}{R^3}\iint (\boldsymbol{R}\cdot\boldsymbol{s})\,\rho_1^{tr}(\boldsymbol{\Delta r_1})\rho_2^{tr}(\boldsymbol{\Delta r_2})d\boldsymbol{\Delta r_1}d\boldsymbol{\Delta r_2} \qquad (S9)$$

Substitute back $\boldsymbol{s} = \boldsymbol{\Delta r_2} - \boldsymbol{\Delta r_1}$ so $(\boldsymbol{R}\cdot\boldsymbol{s}) = \boldsymbol{R}\cdot\boldsymbol{\Delta r_2} - \boldsymbol{R}\cdot\boldsymbol{\Delta r_1}$ and simplify the integral

$$J_1 = \frac{1}{4\pi\epsilon_0}\frac{1}{R^3}\left[\begin{array}{c}\left(\int \rho_1^{tr}(\boldsymbol{\Delta r_1})\,d\boldsymbol{\Delta r_1}\right)\left(\int \boldsymbol{R}\cdot\boldsymbol{\Delta r_2}\rho_2^{tr}(\boldsymbol{\Delta r_2})\,d\boldsymbol{\Delta r_2}\right) - \\ \left(\int \boldsymbol{R}\cdot\boldsymbol{\Delta r_1}\rho_1^{tr}(\boldsymbol{\Delta r_1})\,d\boldsymbol{\Delta r_1}\right)\left(\int \rho_2^{tr}(\boldsymbol{\Delta r_2})\,d\boldsymbol{\Delta r_2}\right)\end{array}\right] \qquad (S10)$$

With the definition of the transition dipole moment according to

$$\boldsymbol{\mu_i} = \int \boldsymbol{\Delta r_i}\rho_i^{tr}(\boldsymbol{\Delta r_i})\,d\boldsymbol{\Delta r_i} \qquad (S11)$$

The first order integral can be simplified to

$$J_1 = -\frac{1}{4\pi\epsilon_0}\frac{1}{R^3}[Q_1(\boldsymbol{R}\cdot\boldsymbol{\mu_2}) - Q_2(\boldsymbol{R}\cdot\boldsymbol{\mu_1})] \qquad (S12)$$

Which is also zero due to the multiplication with the monopole terms. The second order term, which represents the dipole-dipole interaction is

$$J_2 = \frac{1}{4\pi\epsilon_0}\frac{1}{2R^5}\iint [3(\boldsymbol{R}\cdot\boldsymbol{s})^{\boldsymbol{2}} - R^2s^2]\rho_1^{tr}(\boldsymbol{\Delta r_1})\rho_2^{tr}(\boldsymbol{\Delta r_2})d\boldsymbol{\Delta r_1}d\boldsymbol{\Delta r_2} \qquad (S13)$$

Substitute back $\boldsymbol{s} = \boldsymbol{\Delta r_2} - \boldsymbol{\Delta r_1}$ so $(\boldsymbol{R}\cdot\boldsymbol{s}) = \boldsymbol{R}\cdot\boldsymbol{\Delta r_2} - \boldsymbol{R}\cdot\boldsymbol{\Delta r_2}$ and $s^2 = |\boldsymbol{\Delta r_2} - \boldsymbol{\Delta r_1}|^2 = |\boldsymbol{\Delta r_2}|^2 + |\boldsymbol{\Delta r_1}|^2 - 2\boldsymbol{\Delta r_1}\cdot\boldsymbol{\Delta r_2}$ Which results in $3(\boldsymbol{R}\cdot\boldsymbol{s})^{\boldsymbol{2}} - R^2s^2 = (\boldsymbol{R}\cdot\boldsymbol{\Delta r_2} - \boldsymbol{R}\cdot\boldsymbol{\Delta r_2})^{\boldsymbol{2}} - R^2(|\boldsymbol{\Delta r_1}|^2 + |\boldsymbol{\Delta r_2}|^2 - 2\boldsymbol{\Delta r_1}\cdot\boldsymbol{\Delta r_2})$. After expanding the terms and evaluating the integral expression many terms will be zero as in previous steps. The surviving terms are $\boldsymbol{-6}(\boldsymbol{R}\cdot\boldsymbol{\Delta r_1})(\boldsymbol{R}\cdot\boldsymbol{\Delta r_2}) + \boldsymbol{2}R^2(\boldsymbol{\Delta r_2}\cdot\boldsymbol{\Delta r_1})$ which with the definition of the transition dipole moment results in

$$J_2 = \frac{1}{4\pi\epsilon_0}\frac{1}{2R^5}[2R^2(\mu_{\boldsymbol{1}}\cdot\mu_{\boldsymbol{2}}) - 6(\boldsymbol{R}\cdot\mu_{\boldsymbol{1}})(\boldsymbol{R}\cdot\mu_{\boldsymbol{2}})] = \frac{1}{4\pi\epsilon_0}\left[\frac{\boldsymbol{\mu_1}\cdot\boldsymbol{\mu_2}}{R^3} - \frac{3(\boldsymbol{R}\cdot\boldsymbol{\mu_1})(\boldsymbol{R}\cdot\boldsymbol{\mu_2})}{R^5}\right] \qquad (S14)$$

3 Extended dipole approximation derivation

Choose a centre $\mathbf{R_n}$ for molecule n. Represent its transition dipole moment as charges $\pm q_n$ separated by a vector $\mathbf{d_n}$ of length l_n. The separation vector is taken parallel to the dipole direction $\widehat{\boldsymbol{\mu}}_{\boldsymbol{n}}$ so that $\mathbf{d_n} = l_n\boldsymbol{\mu_n}$. Place the charges symmetrically about $\boldsymbol{R_n}$:

$$\mathbf{r}_\mathbf{n}^{\pm} = \boldsymbol{R_n} \pm \frac{1}{2}\boldsymbol{d_n} \qquad (S15)$$

With the transition density represented by $\pm q_n$ at $\mathbf{r}_\mathbf{n}^{\pm}$ the equation for the total interaction between two transition densities as described by

$$\mathrm{J} = \frac{1}{4\pi\epsilon_0}\iint \frac{\rho_1^{\mathrm{tr}}(\mathbf{r_1})\rho_2^{\mathrm{tr}}(\mathbf{r_2})}{|\mathbf{r_1}-\mathbf{r_2}|}\mathrm{d}\mathbf{r_1}\mathrm{d}\mathbf{r_2} \qquad (S16)$$

simplifies to the sum of four charge-charge interactions:

$$J_{ext} = \frac{1}{4\pi\epsilon_0}\left[\frac{(+q_1)(+q_2)}{|\boldsymbol{r}_\mathbf{1}^{+}-\boldsymbol{r}_\mathbf{2}^{+}|} + \frac{(-q_1)(-q_2)}{|\boldsymbol{r}_\mathbf{1}^{-}-\boldsymbol{r}_\mathbf{2}^{-}|} + \frac{(+q_1)(-q_2)}{|\boldsymbol{r}_\mathbf{1}^{+}-\boldsymbol{r}_\mathbf{2}^{-}|} + \frac{(-q_1)(+q_2)}{|\boldsymbol{r}_\mathbf{1}^{-}-\boldsymbol{r}_\mathbf{2}^{+}|}\right] \qquad (S17)$$

Now define the centre-centre vector $\mathbf{R} = \mathbf{R_2} - \mathbf{R_1}$ and with $\mathbf{r}_\mathbf{k}^{\pm} = \boldsymbol{R_k} \pm \frac{\mathbf{1}}{\mathbf{2}}\boldsymbol{d_n}$ rewrite each distance in terms of **R,** $\mathbf{d_1}$, and $\mathbf{d_2}$ which leads to

$$J_{ext} = \frac{q_1q_2}{4\pi\epsilon_0}\left[\frac{1}{\left|\boldsymbol{R}+\frac{d_2}{2}-\frac{d_1}{2}\right|} + \frac{1}{\left|\boldsymbol{R}-\frac{d_2}{2}+\frac{d_1}{2}\right|} - \frac{1}{\left|\boldsymbol{R}-\frac{d_2}{2}-\frac{d_1}{2}\right|} - \frac{1}{\left|\boldsymbol{R}+\frac{d_2}{2}+\frac{d_1}{2}\right|}\right] \qquad (S18)$$

With

$$\boldsymbol{\mu}_\mathbf{n} = \mathrm{q_n}\mathbf{d_n} \rightarrow \mathrm{q_n} = \frac{|\boldsymbol{\mu}_\mathbf{n}|}{\mathrm{l_n}} \qquad (S19)$$

The final expression is reached

$$J_{ext} = \frac{1}{4\pi\epsilon_0}\frac{|\boldsymbol{\mu}_\mathbf{1}||\boldsymbol{\mu}_\mathbf{2}|}{l_1l_2}\left[\begin{array}{c}\frac{1}{\left|\boldsymbol{R}+\frac{l_2}{2}\widehat{\boldsymbol{\mu}}_2-\frac{l_1}{2}\widehat{\boldsymbol{\mu}}_1\right|} + \frac{1}{\left|\boldsymbol{R}-\frac{l_2}{2}\widehat{\boldsymbol{\mu}}_2+\frac{l_1}{2}\widehat{\boldsymbol{\mu}}_1\right|} - \\ \frac{1}{\left|\boldsymbol{R}-\frac{l_2}{2}\widehat{\boldsymbol{\mu}}_2-\frac{l_1}{2}\widehat{\boldsymbol{\mu}}_1\right|} - \frac{1}{\left|\boldsymbol{R}+\frac{l_2}{2}\widehat{\boldsymbol{\mu}}_2+\frac{l_1}{2}\widehat{\boldsymbol{\mu}}_1\right|}\end{array}\right] \qquad (S20)$$

4 Derivation of Hamiltonian matrix entries for a dimer

The derivation of going from the Schrödinger equation with the input wavefunction being a linear combination of basis functions, to a matrix notation containing the already defined matrix elements is here given for a dimer. The wavefunction for the excited state is:

$$\Psi = c_1|1\rangle + c_2|2\rangle \tag{S21}$$

Where the number indicates which molecule that is excited in the two basis functions, $|n\rangle$. The Schrödinger equation for the system is:

$$\hat{H}_F(c_1|1\rangle + c_2|2\rangle) = (\hat{H}_1 + \hat{H}_2 + \hat{H}_{12})(c_1|1\rangle + c_2|2\rangle) = E(c_1|1\rangle + c_2|2\rangle) \tag{S22}$$

Which multiplication with each complex conjugate of the basis functions will be used to build up the matrix (equation 25 in the main manuscript). Multiplication of $\langle 1|$ on both sides' yields:

$$c_1\langle 1|\hat{H}_1|1\rangle + c_1\langle 1|\hat{H}_2|1\rangle + c_1\langle 1|\hat{H}_{12}|1\rangle + c_2\langle 1|\hat{H}_1|2\rangle + c_2\langle 1|\hat{H}_2|2\rangle + c_2\langle 1|\hat{H}_{12}|2\rangle = e_1c_1\langle 1|1\rangle + e_1c_2\langle 1|2\rangle \tag{S23}$$

The red terms are zero because the Hamiltonian for the individual molecular wavefunctions must operate only those wavefunctions, and on the right-hand side, the wavefunctions are orthonormal. Furthermore, the blue term is generally small (compared to the coupling strength). It describes the effect molecule two (in the ground state) has on the energy of the excited state of molecule one.[1] Rewriting this equation using matrix elements (equation 25 in the main manuscript) results in:

$$c_1H_{11} + c_2H_{12} = e_1c_1 \tag{S24}$$

The first row in the Hamiltonian matrix (equation 27 in the main manuscript, but for a 2x2 matrix in the example here) is now obtained. Note that the terms H_{11} and H_{12} correspond to the excitation energy of molecule 1 and the coupling energy between the two dyes, J, respectively. To get the second row, equation 22 needs to be multiplied with $\langle 2|$:

$$c_1\langle 2|\hat{H}_1|1\rangle + c_1\langle 2|\hat{H}_2|1\rangle + c_1\langle 2|\hat{H}_{12}|1\rangle + c_2\langle 2|\hat{H}_1|2\rangle + c_2\langle 2|\hat{H}_2|2\rangle + c_2\langle 2|\hat{H}_{12}|2\rangle = e_2c_1\langle 2|1\rangle + e_2c_2\langle 2|2\rangle \tag{S25}$$

Again, the red terms are zero, and the blue is assumed to be negligibly small, and rewriting using matrix elements yields:

$$c_1H_{21} + c_2H_{22} = e_2c_2 \tag{S26}$$

And the complete coupling matrix for the dimer can be obtained by combining equation 24 and equation 26 into matrix form:

$$HC = \begin{bmatrix} H_{11} & H_{12} \\ H_{21} & H_{22} \end{bmatrix}\begin{bmatrix} c_1 \\ c_2 \end{bmatrix} = \begin{bmatrix} e_1 & e_2 \end{bmatrix}\begin{bmatrix} c_1 \\ c_2 \end{bmatrix} = EC \tag{S27}$$

5 References

1. Parson, W. W. Modern optical spectroscopy: With exercises and examples from biophysics and biochemistry, second edition. *Modern Optical Spectroscopy: With Exercises and Examples from Biophysics and Biochemistry, Second Edition* 1–572 (2015) doi:10.1007/978-3-662-46777-0/COVER.
